# Supplementary material for: An NLR paralog Pit2 generated from tandem duplication of Pit1 fine-tunes Pit1 localization and function
Source: Nat Commun. 2024 May 30;15:4610. doi: 10.1038/s41467-024-48943-5 (PMC11139913; doi:10.1038/s41467-024-48943-5)
Supplement: Supplementary file 1 — Supplementary Information [file 41467_2024_48943_MOESM1_ESM.pdf]

Supplementary Information

An NLR paralog Pit2 generated from tandem duplication of *Pit1* fine-tunes Pit1 localization and function

Yuying Li[1, 2, $], Qiong Wang[2, 3, $], Huimin Jia[4, $], Kazuya Ishikawa[2, 5], Ken-ichi Kosami[2, 6], Takahiro Ueba[7], Atsumi Tsujimoto[7], Miki Yamanaka[7], Yasuyuki Yabumoto[7], Daisuke Miki[2], Eriko Sasaki[8], Yoichiro Fukao[9], Masayuki Fujiwara[10], Takako Kaneko-Kawano[11], Li Tan[2], Chojiro Kojima[12], Rod A. Wing[13], Alfino Sebastian[14], Hideki Nishimura[14], Fumi Fukada[14], Qingfeng Niu[15], Motoki Shimizu[16], Kentaro Yoshida[17], Ryohei Terauchi[16, 17], Ko Shimamoto[7], and Yoji Kawano[2, 14, 18, *]

Li *et al.*, Supplementary Figure 1

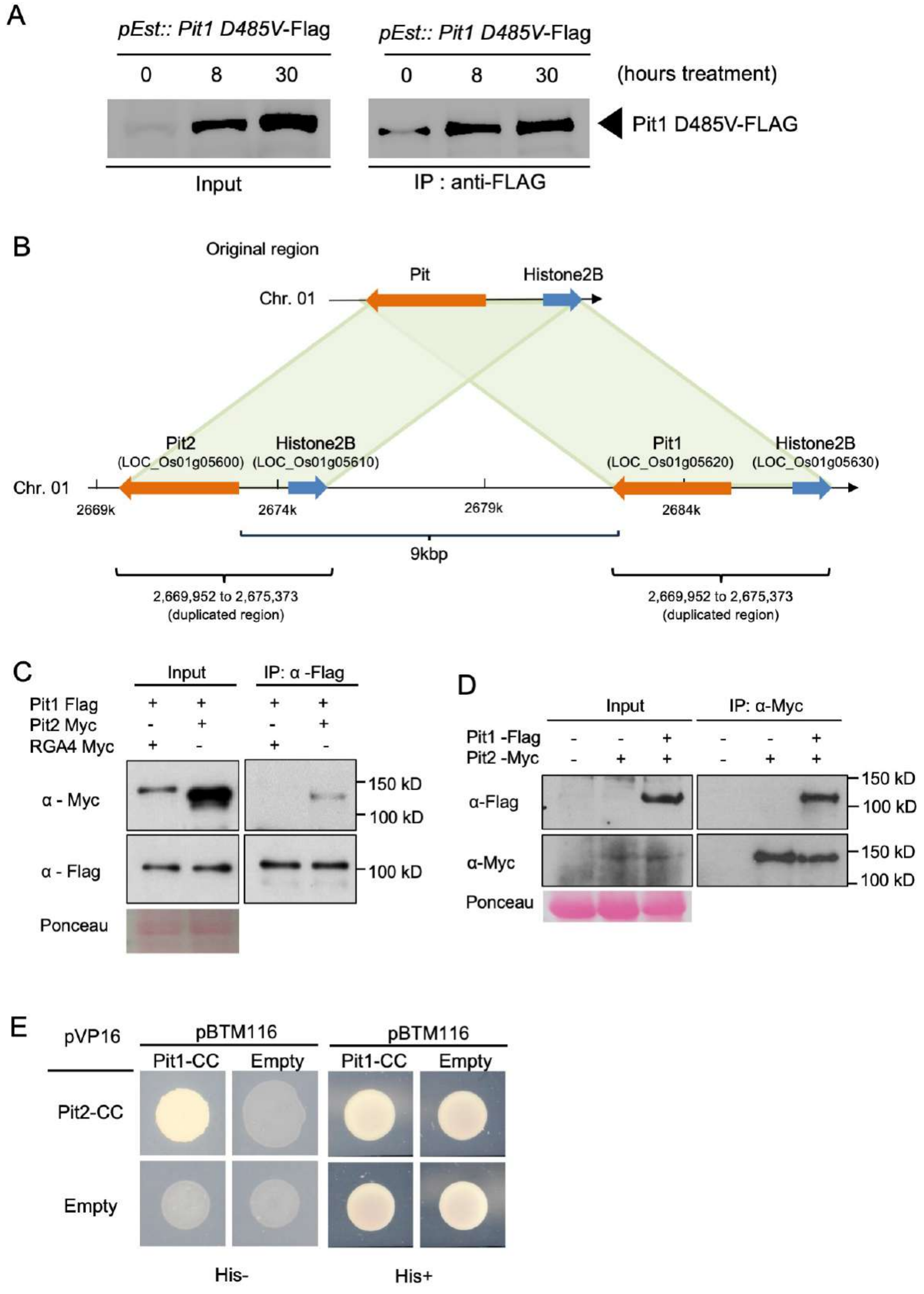

Supplementary Figure 1 Heteromer formation of Pit1 and Pit2

(A) Expression of Pit1 D485V-FLAG driven by an estradiol-inducible promoter in rice suspension cells. Proteins were collected at 0, 8 and 30 h after estradiol treatment and detected by Western blot with anti-FLAG antibody.

(B) The genomic location of *Pit1* and *Pit2* genes.

(C) *In vivo* interaction between full-length Pit1 and Pit2. Pit1-FLAG was transiently co-expressed with Pit2-Myc or the control RGA4-Myc in rice protoplasts. Protoplasts were collected 16 h after transfection, and proteins extracted. Co-IP was performed using anti-FLAG agarose beads, and the proteins were detected by Western blot with anti-Myc and anti-FLAG antibodies. Ponceau staining of Rubisco served as a loading control.

(D) *In vivo* interaction of full-length Pit1 and Pit2. Pit1-Flag and Pit2-Myc were expressed by their own promoters in rice plants. Co-IP was performed using anti-Myc agarose beads, and the proteins were detected by immunoblot with anti-Myc and anti-Flag antibodies. Ponceau staining of Rubisco served as a loading control.

(E) Interaction between the CC domains of Pit1 and Pit2. Yeast growth on selective plates without histidine [His (−)] indicates a specific interaction. All images are representative of results repeated three times with similar results. Source data are provided as a Source Data file.

Li *et al.,* Supplementary Figure 2

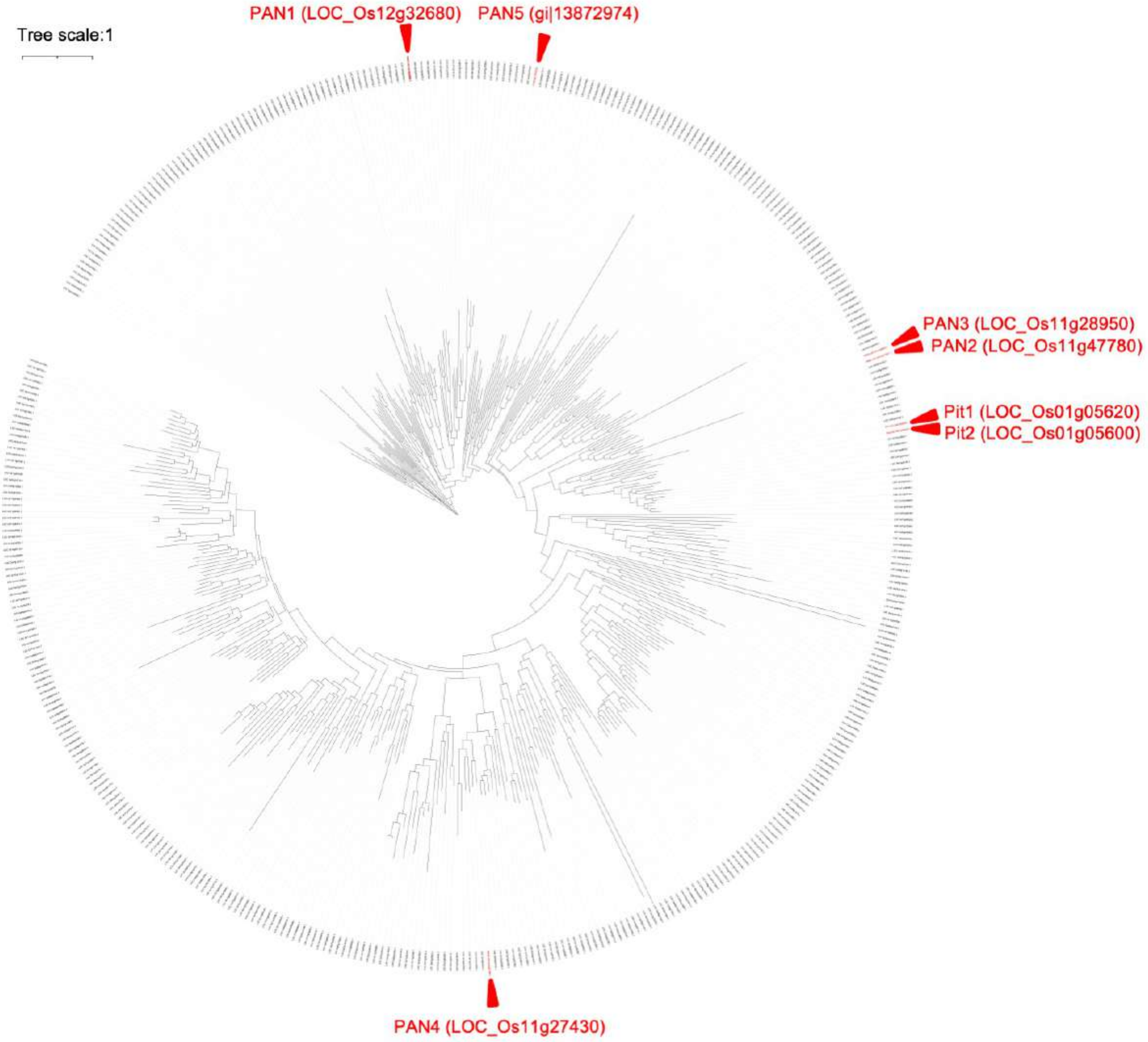

Supplementary Figure 2 Phylogenetic tree of rice NLR proteins with Pit1, Pit2, and PAN1-5

The sequence of 430 NLR proteins was downloaded from the Rice Genome Annotation Project (http://rice.uga.edu/) (Ding, L. *et al. Physiological and Molecular Plant Pathology* **111**, 101488 (2020)). The protein sequences of Pit1, Pit2, PAN1-5, and the other NLR proteins were aligned using MUSCLE, and FastTreeMP was used to construct the phylogenetic tree using the maximum likelihood (ML) method.

Li *et al.*, Supplementary Figure 3

A

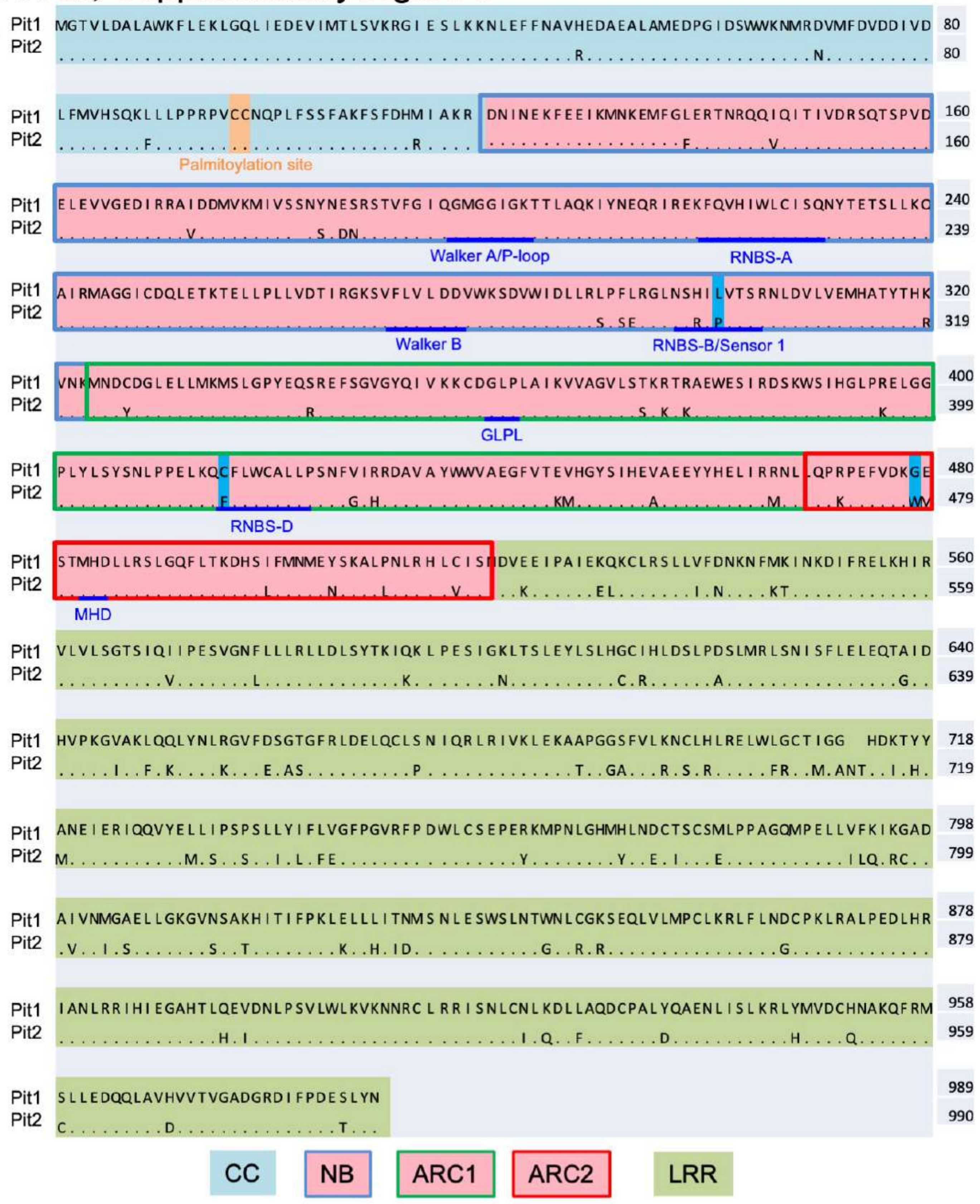

B

Pit2

MGTVLDALAWKFLEKLGQLIEDEVIMTLSVKRGIESLKKNLEFFNAVREDAEALAMEDPGIDSWWKNMRNVMFDVDDI
VDLFMVHSQKFLLPPRPVCCNQPLFSSFAKFSFDHRIAKRIDNINEKFEEIKMNKEMFGFERTNRQQVQITIVDRSQTSP
VDELEVVGEDIRRAVDDMVKMIVSNSNDNRSTVFGIQGMGGIGKTTLAQKIYNEQRIREKFQVHIWLCISQNYTETSLLK
QAIRMAGGICDQLETKTELLPLLVDTIRGKSVFLVLDDVWKSDVWIDLLRSPSERGLNSRIPVTSRNLDVLVEMHATYTH
RVNKMNDYDGLELLMKMSLGPYEQRREFSGVGYQIVKKCDGLPLAIKVVAGVLSSKKTKAEWESIRDSKWSIHGLPKE
LGGPLYLSYSNLPPELKQFFLWCALLPSNFGIHRDAVAYWWVAEGFVTKMHGYSIHEAAEEYYHELIRMNLLQPKPEF
VDKWMSTMHDLLRSLGQFLTKDHSLFMNMENSKALLNLRHLVISNDVKEIPAIEELKCLRSLLIFNNKNFKTINKDIFREL
KHIRVLVLSGTSIQVIPESVGNLLLLRLLDLSYTKIKKLPESIGNLTSLEYLSLHCCRHLDSLPASLMRLSNISFLELEQTGI
DHVPKGIAKFQKLYNLKGVFESASGFRLDELQCLPNIQRLRIVKLEKATPGGAFVLRNSLRLRELWFRCTMGANTHDIT
HYQMNEIERIQQVYEMLSPSSSLIYLFFEGFPGVRFPDWLCSEPEYKMPNLGHMYLNECISCSELPPAGQMPELLILQI
RCADAVVNIGSELLGKGVSSATHITIFPKLKLLHIIDMSNLESWSLNTGNLRGRSEQLVLMPCLKRLFLNGCPKLRALPE
DLHRIANLRRIHIEGAHTLHEIDNLPSVLWLKVKNNRCLRRISNLCILQDLFAQDCPALDQAENLISLKRLHMVDCQNAKQ
FRMCLLEDQQLAVDVVTVGADGRDIFPDETLYN

Supplementary Figure 3 Sequence of Pit1 and Pit2 proteins

(A) Sequence alignment of Pit1 and Pit2 proteins. The dark blue background marks the three important residues found in this study.

(B) Pit2 peptides identified in the elution fraction of Pit D485V by MS analysis. Blue and red characters indicate Pit2 peptides detected by MS analysis. Blue character shows the conserved peptides between Pit1 and Pit2. Red character represents Pit2-specific peptide.

Li *et al.*, Supplementary Figure 4

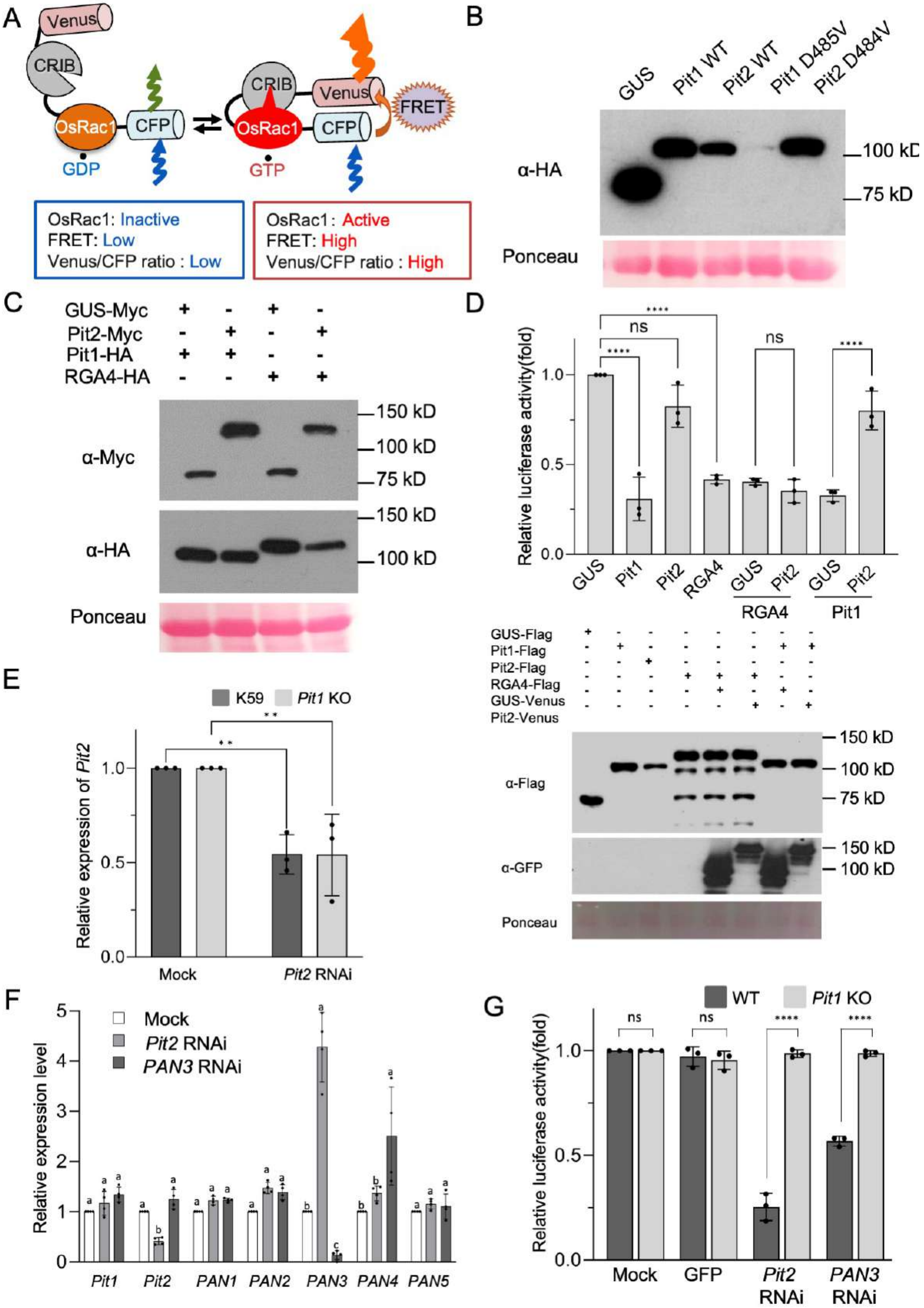

Supplementary Figure 4 Pit2 suppresses Pit1-mediated cell death

(A) Schematic representation of the Raichu–OsRac1 system used to monitor the activation level of OsRac1 *in vivo.*

(B) Expression of HA-tagged Pit1 WT, Pit2 WT and corresponding MHD mutant proteins. The indicated HA-tagged proteins were transiently expressed in *N. benthamiana*. Proteins were detected by Western blot with anti-HA antibody. Ponceau staining of Rubisco served as a loading control.

(C) The indicated combinations of proteins were transiently expressed in *N. benthamiana* leaves. Proteins were detected by Western blot with anti-HA and anti-Myc antibodies. Ponceau staining of Rubisco served as a loading control.

(D) Cell death activity and protein expression of the indicated constructs in rice protoplasts. Relative luciferase activity (GUS = 1) is shown in the upper panel. Bars represent the mean ± s.d. ($n = 3$ biological replicates). The asterisks indicate significant differences as assessed by one-way ANOVA (with Tukey's test) (****$P < 0.0001$, ns indicates no significant difference). The lower panel shows that the tagged proteins were properly expressed in rice protoplasts and detected by Western blot with anti-FLAG and anti-GFP antibodies. Ponceau staining of Rubisco served as a loading control.

(E) Transcript levels of *Pit2* were measured by qRT-PCR and normalized with endogenous *OsUbq* expression at 40 h after transfection. Bars represent the mean ± s.d. ($n = 3$ biological replicates). The asterisks indicate significant differences as assessed by two-way ANOVA (with Šídák's test) (**$P < 0.01$).

(F)The expression level of *Pit1*, *Pit2*, and *PAN1-5* were measured by qRT-PCR. Relative mRNA expression (Mock = 1) is shown. Bars represent the mean ± s.d. ($n = 4$ biological replicates). Different letters above bars indicate a significant difference determined by two-way ANOVA (with Tukey's test) ($P < 0.05$).

(G) Rice protoplasts from the cultivar K59 WT or *Pit1* KO were co-transfected with RNAi constructs against *PAN3* and the *luciferase* reporter vector. *Luciferase* activity was measured 40 h after transfection. Relative luciferase activity (Mock = 1) is shown. Bars represent the mean ± s.d. ($n = 3$ biological replicates). The asterisks indicate significant differences as assessed by two-way ANOVA (with Šídák's test) (****$P < 0.0001$, ns indicates no significant difference). All images are representative of results repeated three times with similar results. Source data are provided as a Source Data file.

Li *et al.*, Supplementary Figure 5

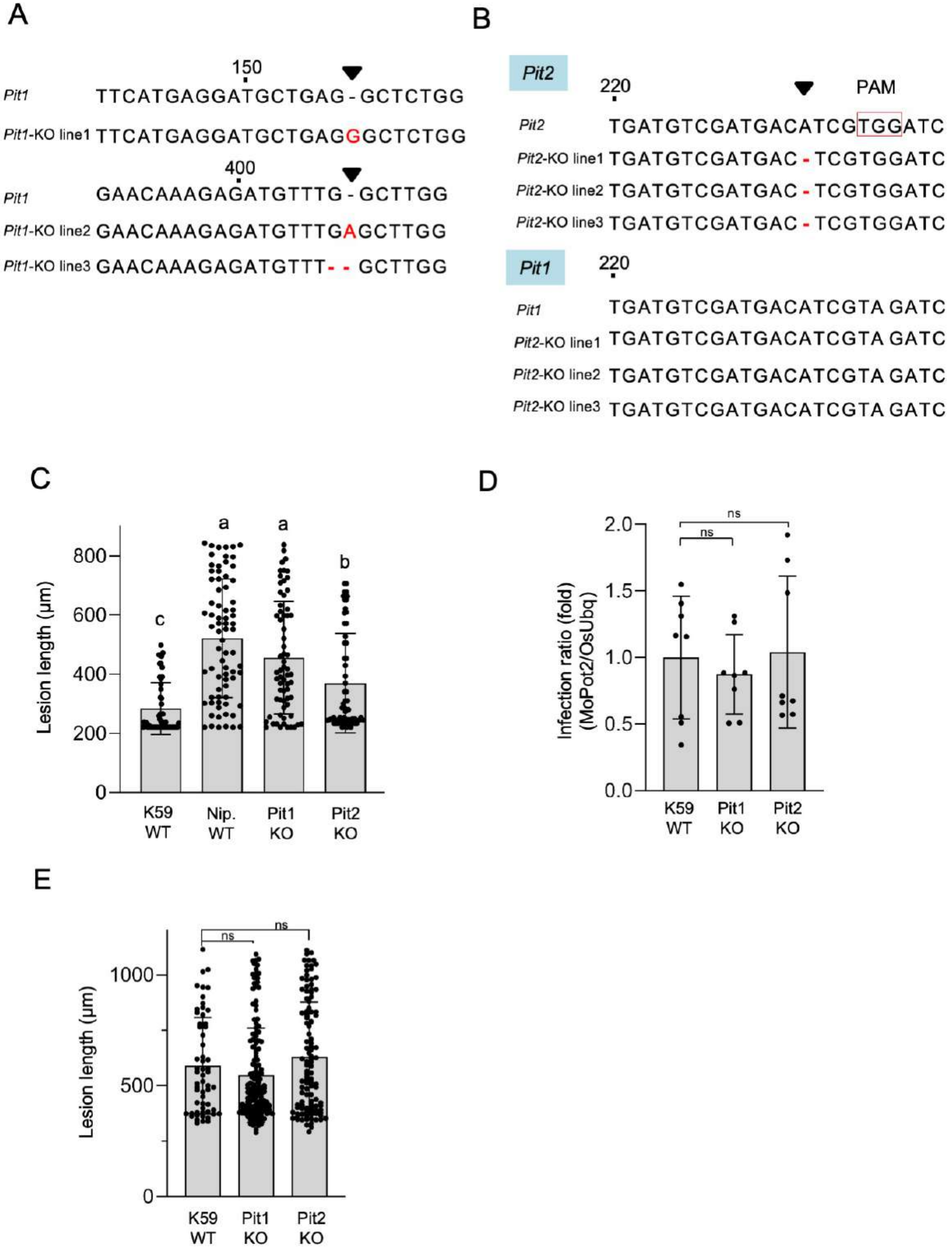

Supplementary Figure 5 Infection assay of *Pit1* or *Pit2* KO plants.

(A and B) Sequence alignment showing CRISPR/Cas9 mediated mutations (black arrow) to generate *Pit1* (A)and *Pit2* (B) KO plants.

(C) Quantitative analysis of lesions induced by the incompatible *M. oryzae* race 007.0 at 7 dpi. Bars represent the mean ± s.d. (n = 53, 74, 66, 62 infected sites in K59 WT, Nip.WT, Pit1 KO, Pit2 KO, respectively). Different letters above bars indicate a significant difference determined by one-way ANOVA (with Tukey's test) ($P < 0.05$).

(D) Growth of the compatible *M. oryzae* (IB14-1K-1) was measured by qPCR and normalized with endogenous *OsUbq*. Relative infection ratio (K59 WT = 1) is shown. Bars represent the mean ± s.d. (n = 8 independent plants). ns indicates no significant difference.

(E) Quantitative analysis of lesions induced by the compatible *M. oryzae* (IB14-1K-1) at 7 dpi. Bars represent the mean ± s.d. (n = 58, 149, 110 infected sites in K59 WT, Pit1 KO, Pit2 KO, respectively). ns indicates no significant difference. Source data are provided as a Source Data file.

Li *et al.*, Supplementary Figure 6

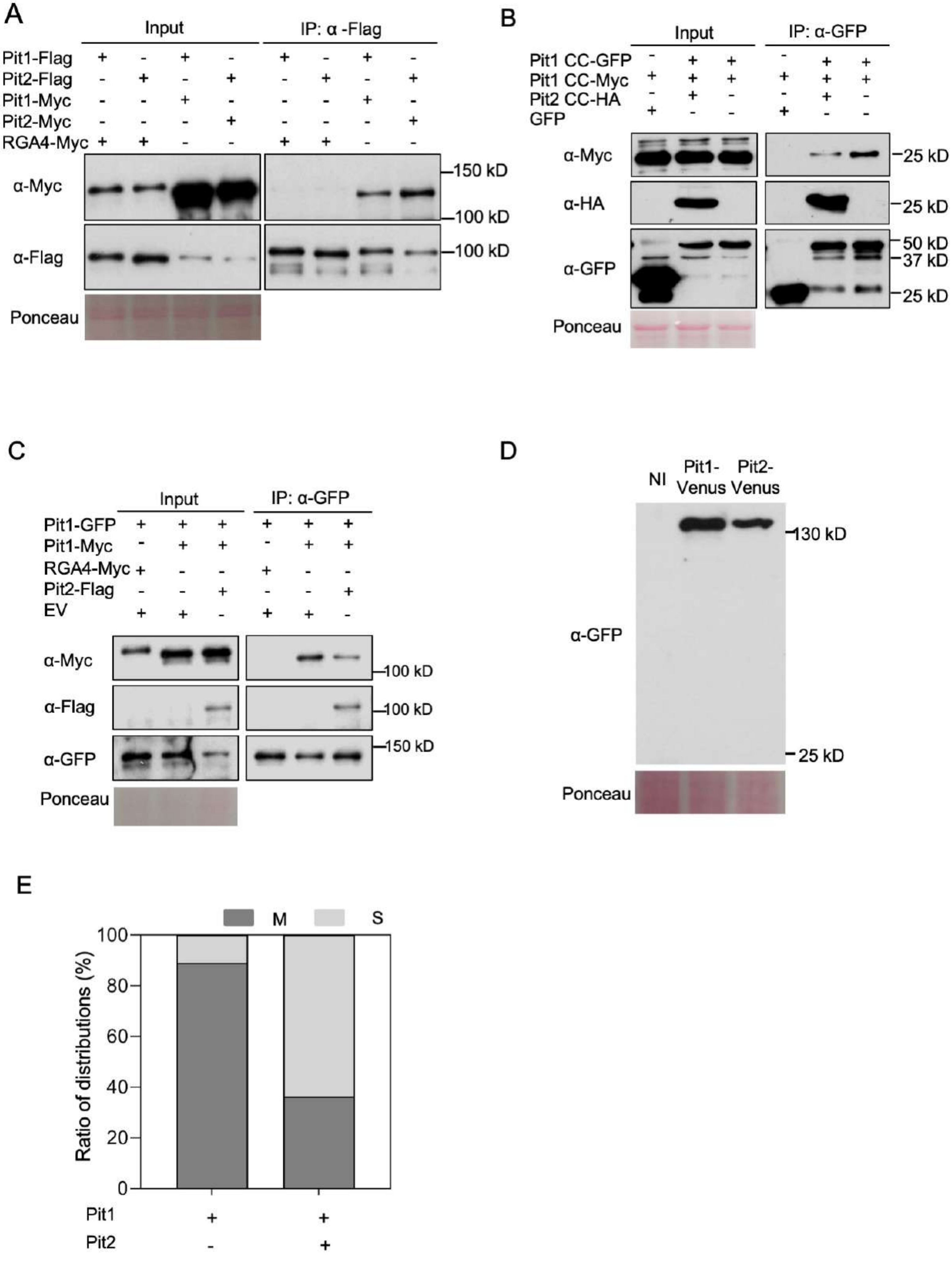

Supplementary Figure 6 Pit2 competes with Pit1 for binding to Pit1

(A) *In vivo* self-association of full-length of Pit1 and Pit2. The indicated protein combinations were transiently co-expressed in rice protoplasts. Co-IP was performed using anti-FLAG agarose beads. The proteins were detected by Western blot with anti-Myc and anti-FLAG antibodies. Ponceau staining of Rubisco served as a loading control.

(B) Pit2 CC competes with Pit1 CC to form heteromers *in vivo.* Transiently co-expression of Pit1 CC-GFP and Pit1 CC-Myc in the presence or absence of Pit2 CC-HA in *N. benthamiana*. Co-IP was performed using anti-GFP agarose beads, and the proteins were detected by Western blot with relevant antibodies. Ponceau staining of Rubisco served as a loading control.

(C) Pit2 competes with Pit1 to form heteromers *in vivo.* Pit1-GFP was transiently expressed with Pit1-Myc in the presence or absence of Pit2-FLAG in rice protoplasts. Co-IP was performed using anti-GFP agarose beads, and the proteins were detected by Western blot with relevant antibodies. Ponceau staining of Rubisco served as a loading control.

(D) Western blot shows protein expression of Pit1-Venus and Pit2-Venus with anti-GFP antibody. Pit1-Venus and Pit2-Venus were transiently expressed in rice protoplasts. NI indicates non-infiltrated protoplasts as a negative control. Ponceau staining of Rubisco served as a loading control.

(E) Statistical analysis of the cell fractionation assay related to Figure 2G. S and M indicate soluble fraction and microsomal fraction, respectively. All images are representative of results repeated three times with similar results. Source data are provided as a Source Data file.

Li *et al.,* Supplementary Figure 7

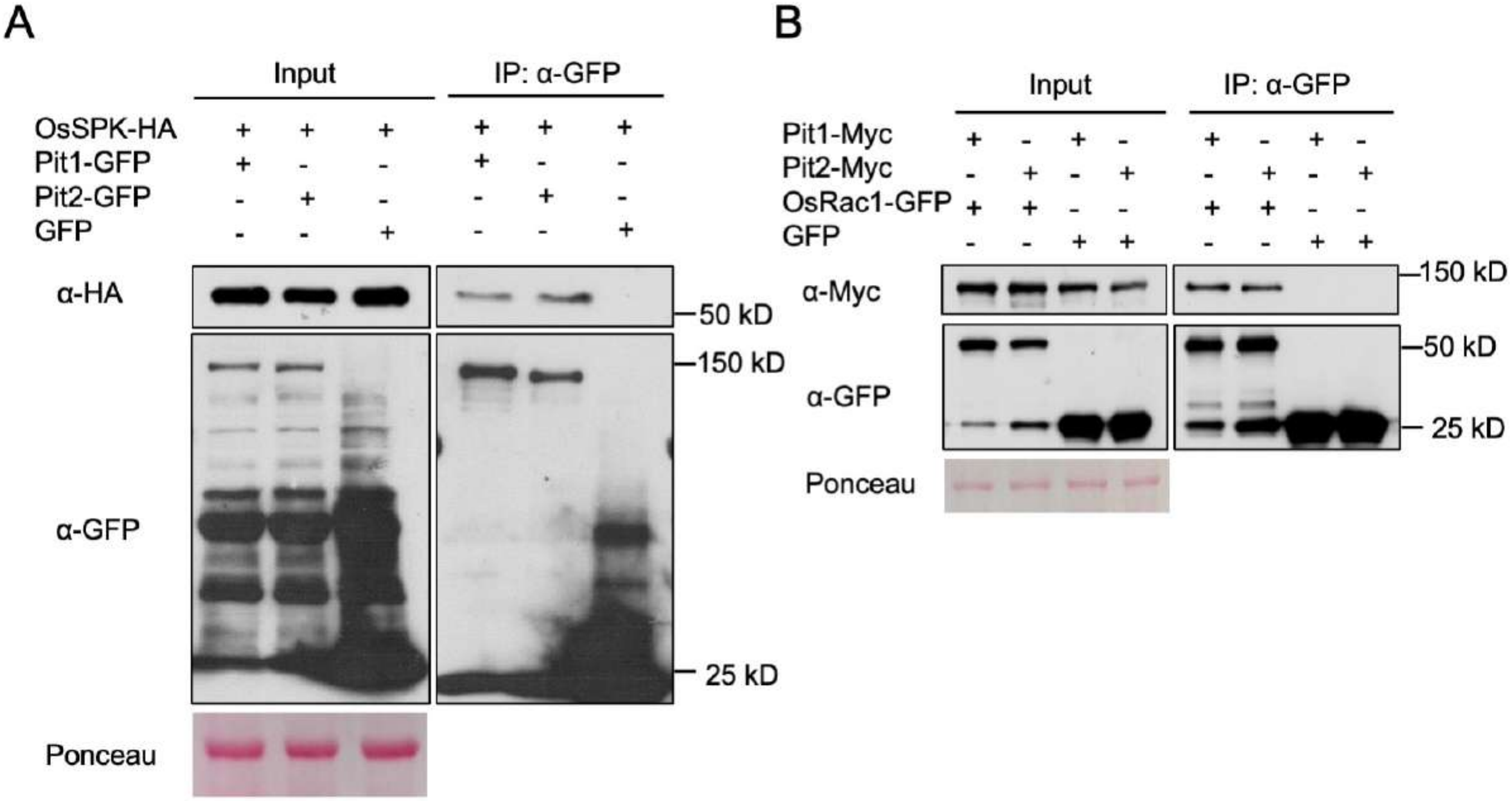

Supplementary Figure 7 Both Pit1 and Pit2 interact with OsSPK1 and OsRac1

(A) *In vivo* interaction between Pit1 or Pit2 and OsSPK1 (amino acids 1334–1835). Pit1-GFP or Pit2-GFP was transiently expressed with OsSPK1-HA (amino acids 1334–1835) in *N. benthamiana*. Co-IP was performed using anti-GFP agarose beads, and the proteins were detected by Western blot with anti-HA and anti-GFP antibodies. Ponceau staining of Rubisco served as a loading control.

(B) *In vivo* interaction between Pit1 or Pit2 and OsRac1. Co-IP and protein detection were tested as described in (A). Ponceau staining of Rubisco served as a loading control. All images are representative of results repeated three times with similar results. Source data are provided as a Source Data file.

Li *et al.*, Supplementary Figure 8

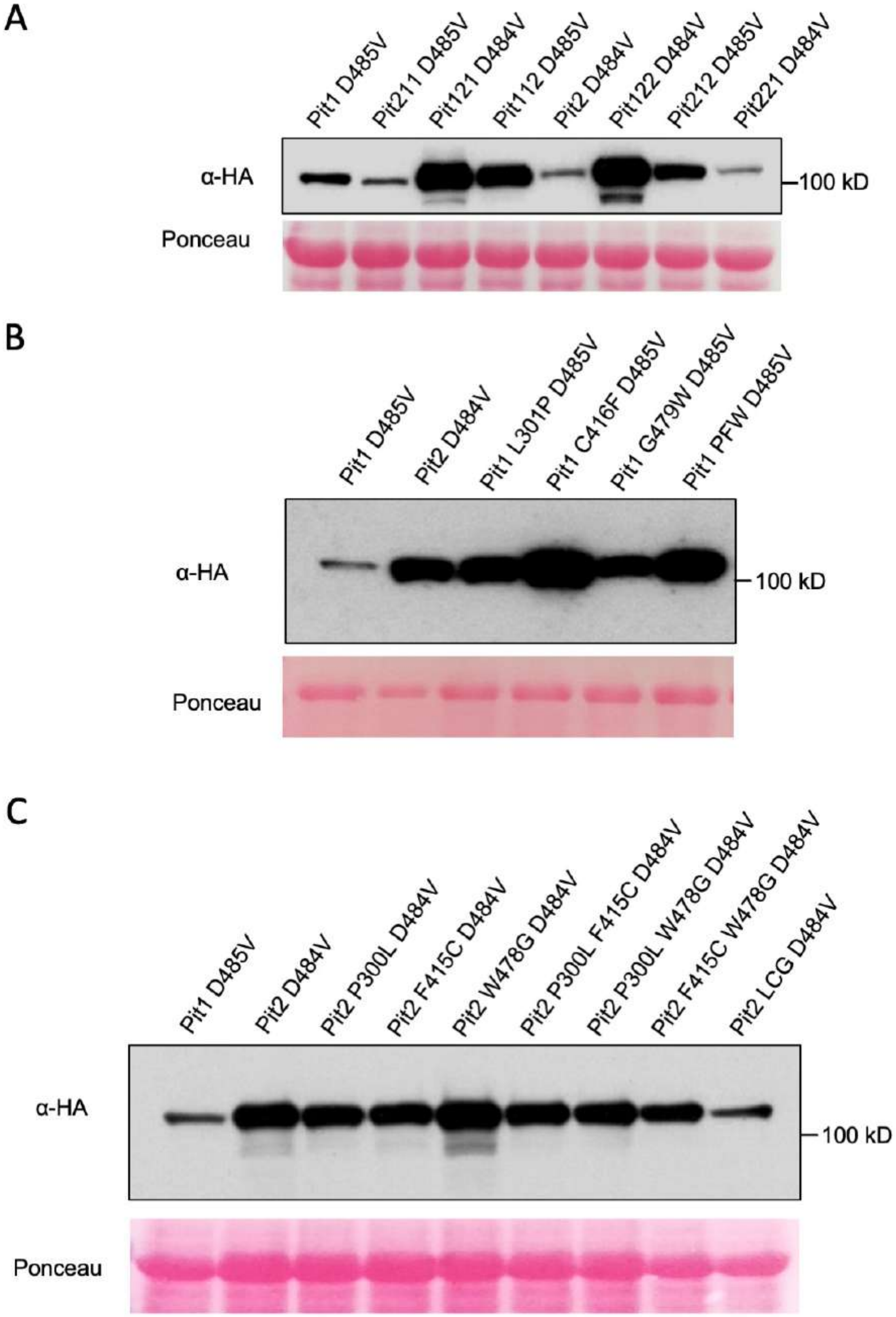

Supplementary Figure 8 Expression of domain-swapping proteins and substitution mutation proteins in *N. benthamiana*

(A) Expression of HA-tagged Pit1 D485V, Pit2 D484V and domain-swapping mutants. The indicated HA-tagged proteins were transiently expressed in *N. benthamiana*. Proteins were detected by Western blot with anti-HA antibody. Ponceau staining of Rubisco served as a loading control.

(B and C) Expression levels of HA-tagged Pit1 (B) and Pit2 (C) mutants. The indicated proteins were tested as described in (A). Ponceau staining of Rubisco served as a loading control. All images are representative of results repeated three times with similar results. Source data are provided as a Source Data file.

Li *et al.*, Supplementary Figure 9

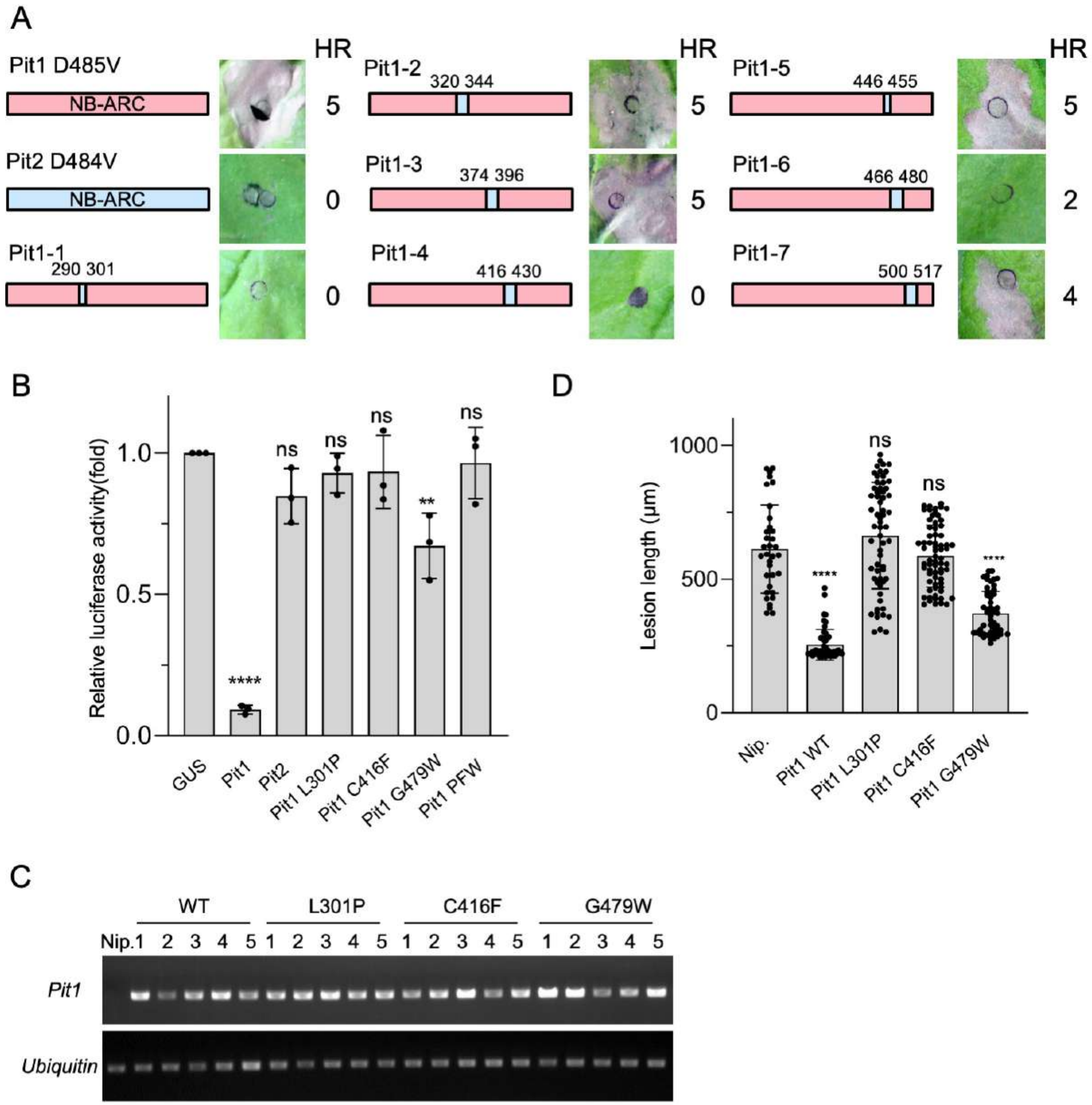

Supplementary Figure 9 Effects of domain-swapping mutants between Pit1 and Pit2 on cell death and immunity

(A) Cell death phenotype of Pit1 D485V and the NB-ARC domain-swapping mutants in *N. benthamiana*, photographed at 3 dpi.

(B) Cell death activity of the Pit1 amino acid substitution mutants in rice protoplasts. Relative luciferase activity (GUS = 1) is shown. Bars represent the mean ± s.d. ($n = 3$ biological replicates). The asterisks indicate significant differences (versus GUS) as assessed by one-way ANOVA (with Tukey's test) ($^{**}P < 0.01$, $^{****}P < 0.0001$, ns indicates no significant difference).

(C) RT-PCR shows the transcript levels of exogenous *Pit1* WT and *Pit1* substitution mutants. Numbers indicate independent transgenic lines. *OsUbiquitin* served as an internal control.

(D) Quantitative analysis of lesions induced by the incompatible *M. oryzae* race 007.0 at 7 dpi. Bars represent the mean ± s.d. (n = 34, 53, 62, 68, 53 infected sites in Nip., Pit1 WT, Pit1 L301P, Pit1 C416F, Pit1 G479W, respectively). The asterisks indicate significant differences (versus Nip.) as assessed by one-way ANOVA (with Tukey's test) ($^{****}P < 0.0001$, ns indicates no significant difference). Source data are provided as a Source Data file.

Li *et al.,* Supplementary Figure 10

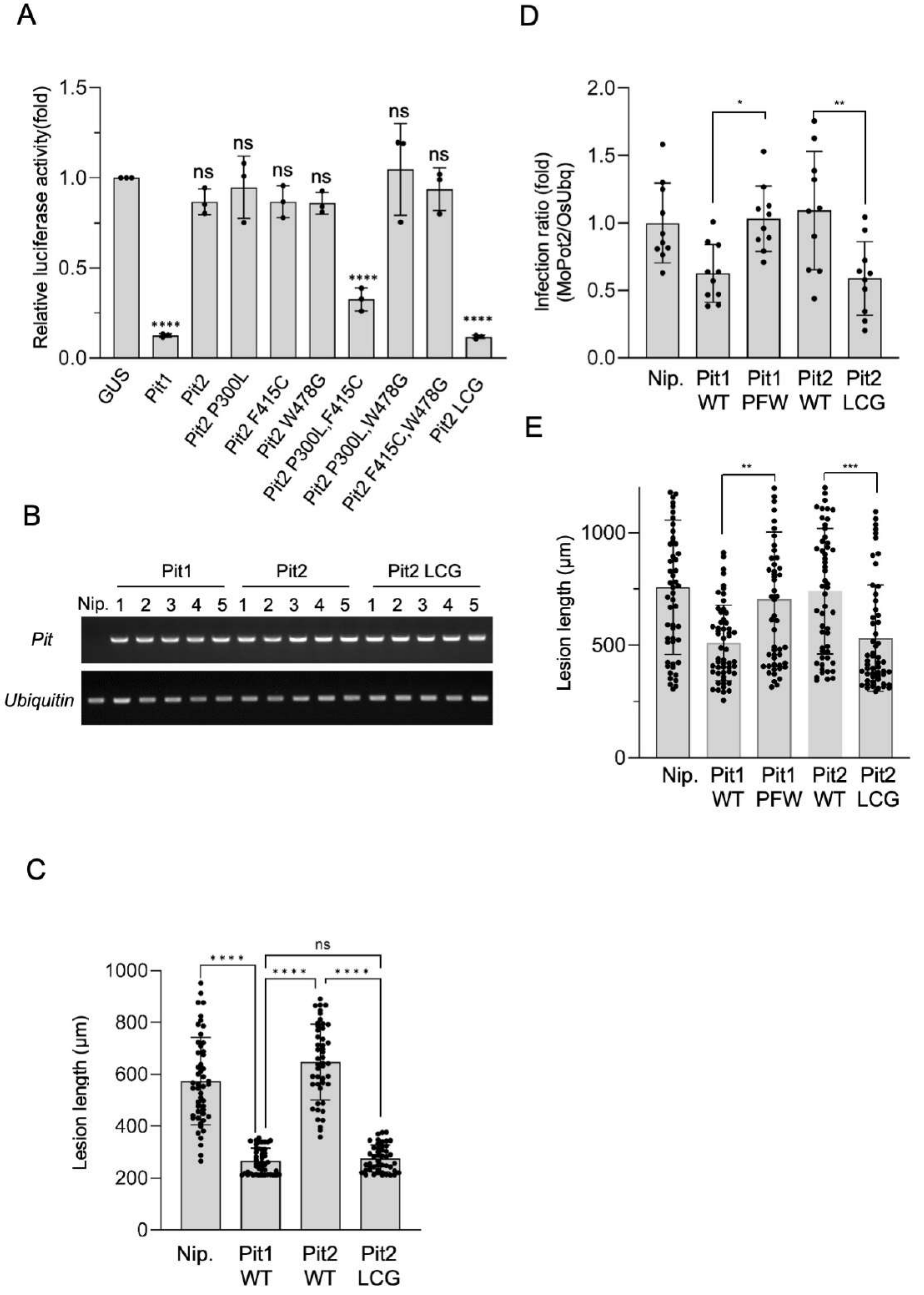

Supplementary Figure 10 Effects of Pit2 mutants on cell death and immunity

(A) Cell death activity of Pit2 mutants in rice protoplasts. Relative luciferase activity (GUS = 1) is shown. Bars represent the mean ± s.d. ($n = 3$ biological replicates). The asterisks indicate significant differences (versus GUS) as assessed by one-way ANOVA (with Tukey's test) (****$P <$ 0.0001, ns indicates no significant difference).

(B) RT-PCR shows the transcript levels of exogenous *Pit1 WT*, *Pit2 WT* and *Pit2 LCG* mutant. Numbers indicate independent transgenic lines. *OsUbiquitin* served as an internal control.

(C) Quantitative analysis of lesions induced by the incompatible *M. oryzae* race 007.0 at 7 dpi. Relative lesion length of the *Pit2* mutants (Nip. = 1) is shown. Bars represent the mean ± s.d. (n = 50, 46, 47, 46 infected sites in Nip., Pit1 WT, Pit2 WT, Pit2 LCG, respectively). The asterisks indicate significant differences as assessed by one-way ANOVA (with Tukey's test) (****$P < 0.0001$, ns indicates no significant difference).

(D) Growth of the incompatible *M. oryzae* race (Race 101.1: MAFF101524) was measured by qPCR and normalized with endogenous *OsUbq*. Relative infection ratio (Nip. = 1) is shown. Bars represent the mean ± s.d. ($n = 10$ independent plants). The asterisks indicate significant differences as assessed by one-way ANOVA (with Tukey's test) (*$P < 0.05$, **$P < 0.01$).

(E) Quantitative analysis of lesions induced by the incompatible *M. oryzae* (Race 101.1: MAFF101524) at 7 dpi. Bars represent the mean ± s.d. (n = 54, 55, 53, 54, 55 infected sites in Nip., Pit1 WT, Pit1 PFW, Pit2 WT, Pit2 LCG, respectively). The asterisks indicate significant differences as assessed by one-way ANOVA (with Tukey's test) (**$P < 0.01$, ***$P < 0.001$). Source data are provided as a Source Data file.

Li *et al.*, Supplementary Figure 11

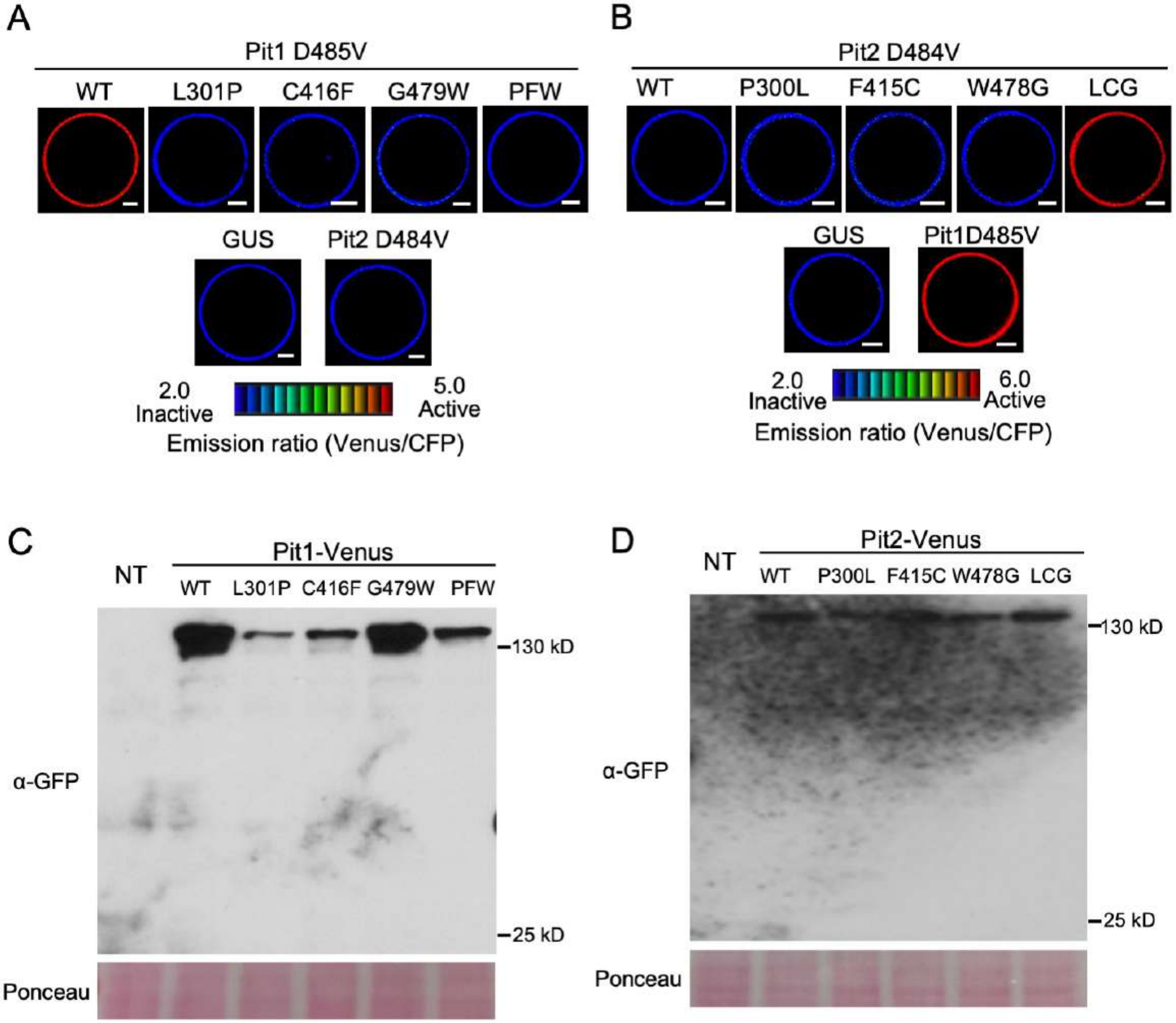

Supplementary Figure 11 Emission ratio images of OsRac1 activation and protein expression of Pit1/2 WT and their mutants

(A and B) Monitoring OsRac1 activation by Pit1, Pit2 and amino acid substitution Pit1 (A) and Pit2 (B) mutants using Raichu-OsRac1 FRET *in vivo.* Emission ratio images of confocal laser-scanning micrographs of rice protoplasts co-expressing Raichu-OsRac1 and the indicated constructs. The colour scale from blue to red indicates low to high activation levels of OsRac1. Scale bars, 5 μm.

(C-D) Western blot shows the transient expression of the inactive form of Pit1-Venus (C) and the active form of Pit2-Venus (D) in rice protoplasts, detected by anti-GFP antibody. NI indicates non-infiltrated protoplasts as a negative control. Ponceau staining of Rubisco served as a loading control. All images are representative of results repeated three times with similar results. Source data are provided as a Source Data file.

Li *et al.*, Supplementary Figure 12

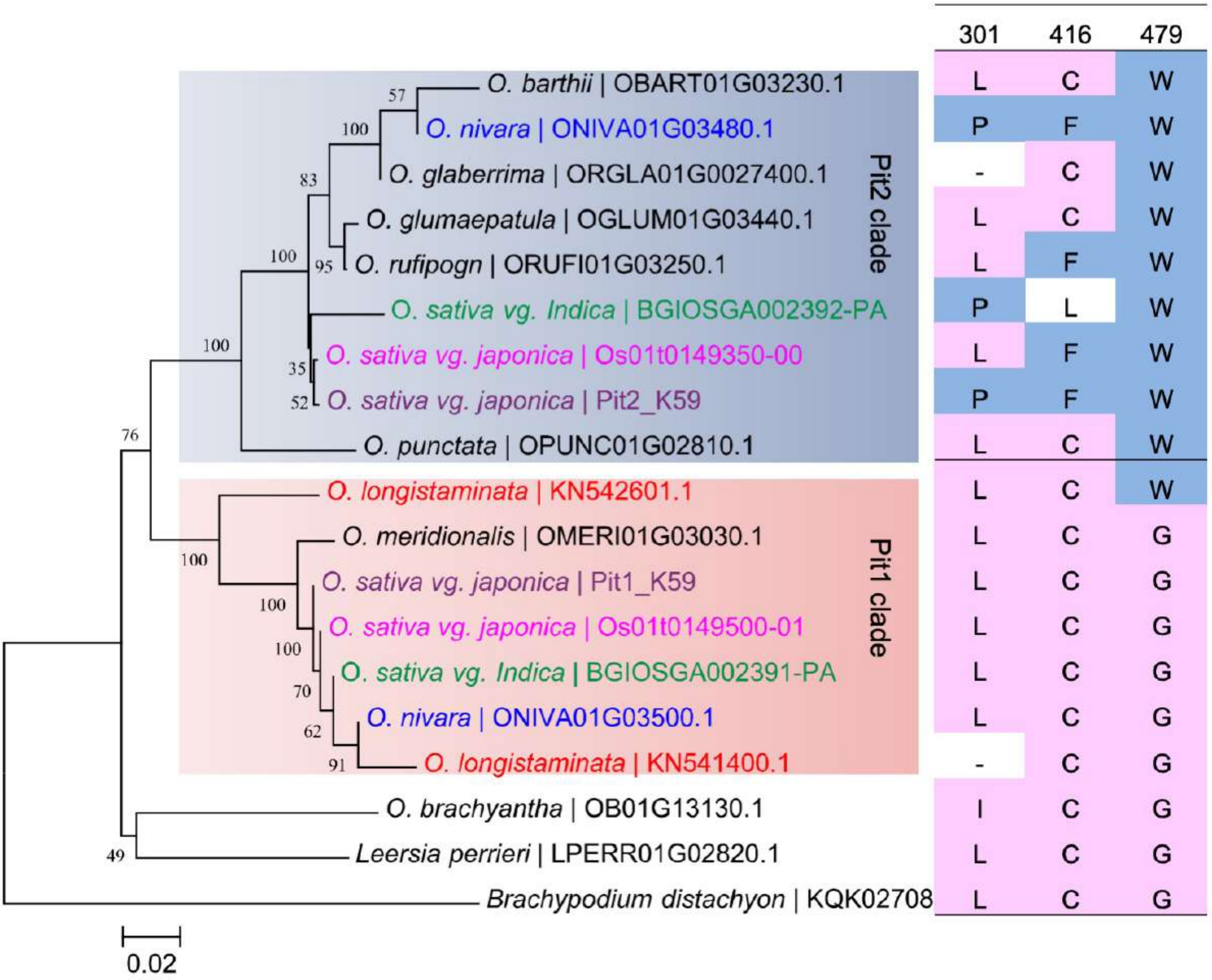

Supplementary Figure 12 Phylogenetic tree of *Pit* genes in 13 *Oryza* species

Species with two *Pit* genes are highlighted in the same colour: red, *O. longistaminata*; blue, *O. nivara*; pink, *O. sativa vg. Japonica*; green, *O. sativa vg. Indica*; purple, *O. sativa vg. Japonica* cultivar K59. The three residues corresponding to L301, C416 and G479 of all proteins are shown to the right.

Li *et al.,* Supplementary Figure 13

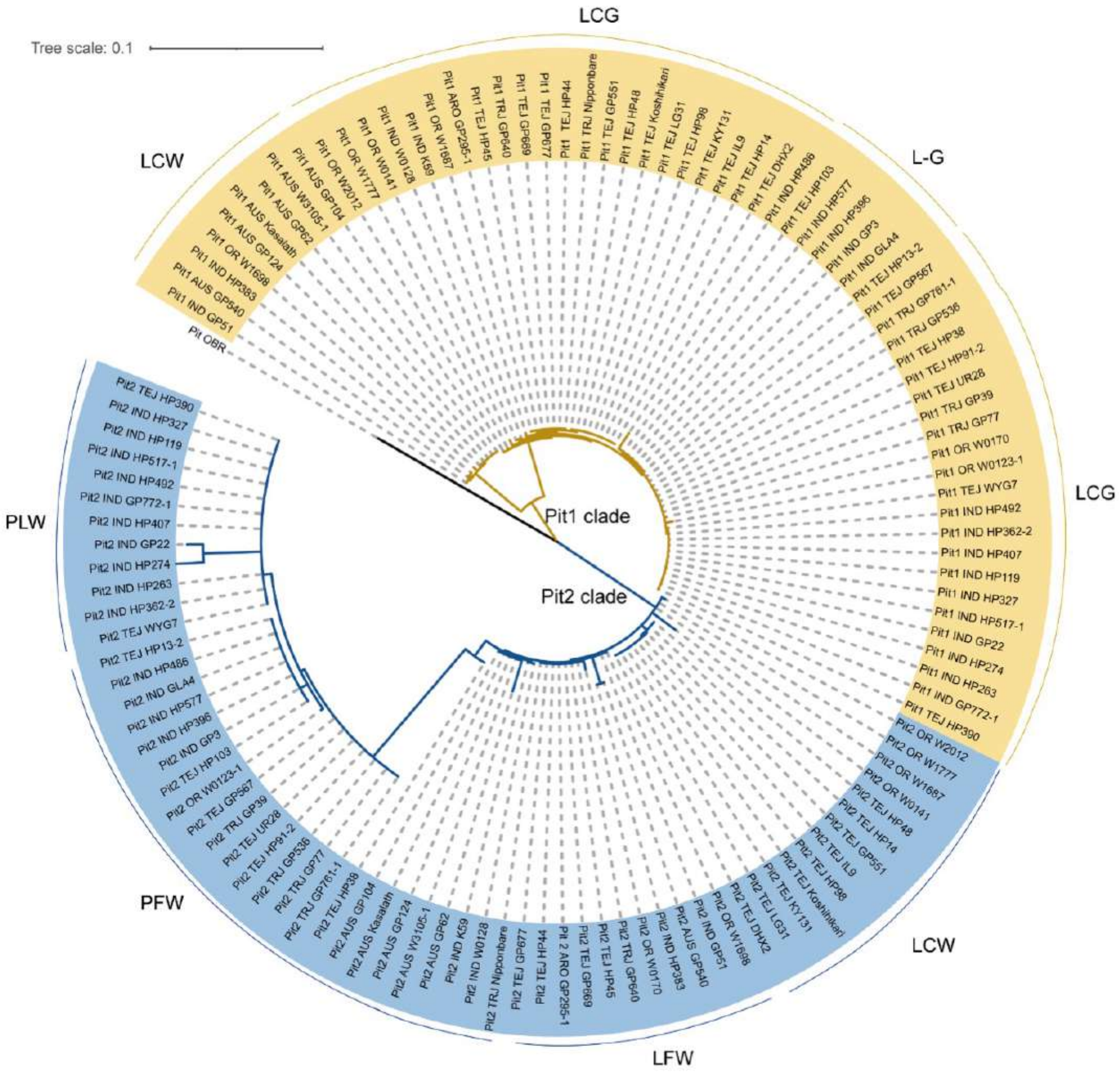

TEJ: *O. sativa* (temperate japonica)

TRJ: *O. sativa* (tropical japonica)

ARO: *O. sativa* (aromatic japonica)

IND: *O. sativa* indica

AUS: *O. sativa* aus

OR: *O. rufipogon*

Supplementary Figure 13 Phylogenetic tree of *Pit1* and *Pit2* alleles in pan-genome rice

The composition of the three important residues (LCG) is shown.

Li *et al.*, Supplementary Figure 14

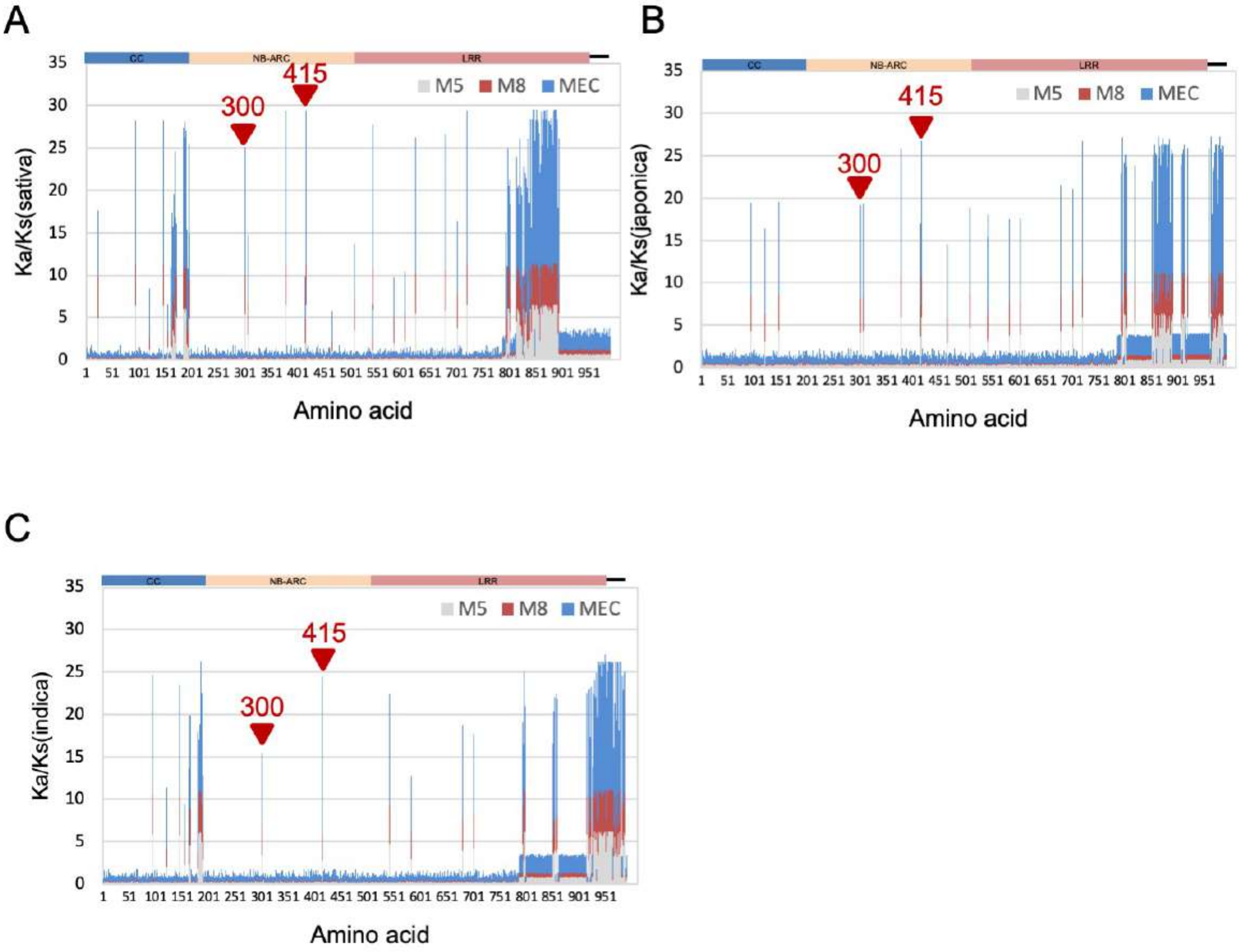

Supplementary Figure 14 Sliding windows of positive selection sites of the *Pit2* alleles under M5, M8 and MEC models

The X-axes indicate the position of the Pit2 amino acids in the site; the Y-axes indicate the ratio of the rate of nonsynonymous substitution (Ka) to the rate of synonymous substitution (Ka) (Ka/Ks). (A) Cultivated rice *O. sativa*; (B) cultivated rice *O. sativa vg. Japonica*; (C) cultivated rice *O. sativa* vg. *Indica*. The important amino acid sites (P300, F415) are highlighted.

Li *et al.*, SupplementaryFigure 15

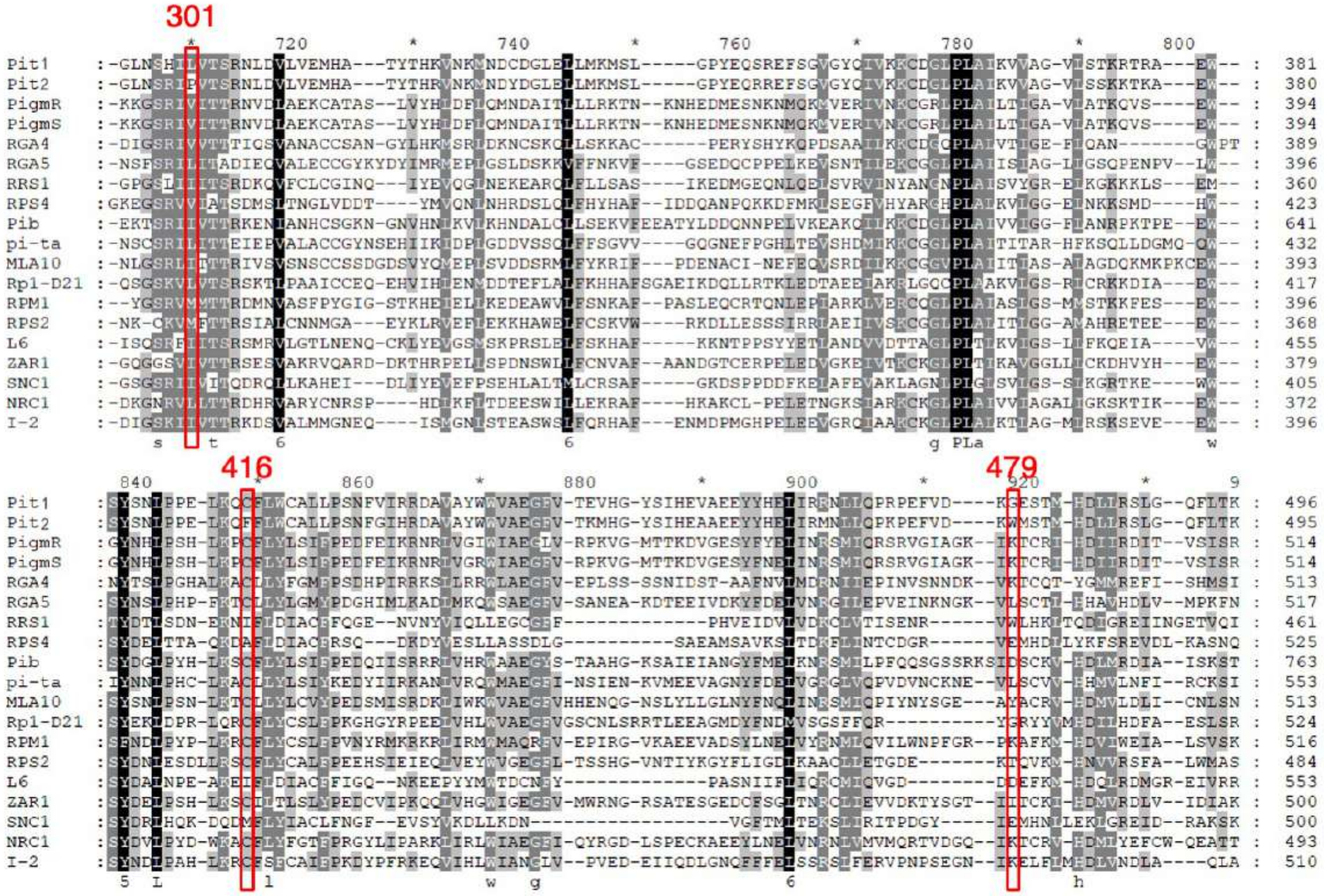

Supplementary Figure 15 Multiple sequence alignments of NLR proteins in various species

The three important Pit1 residues are highlighted with red frames.

Li *et al.,* Supplementary Figure 16

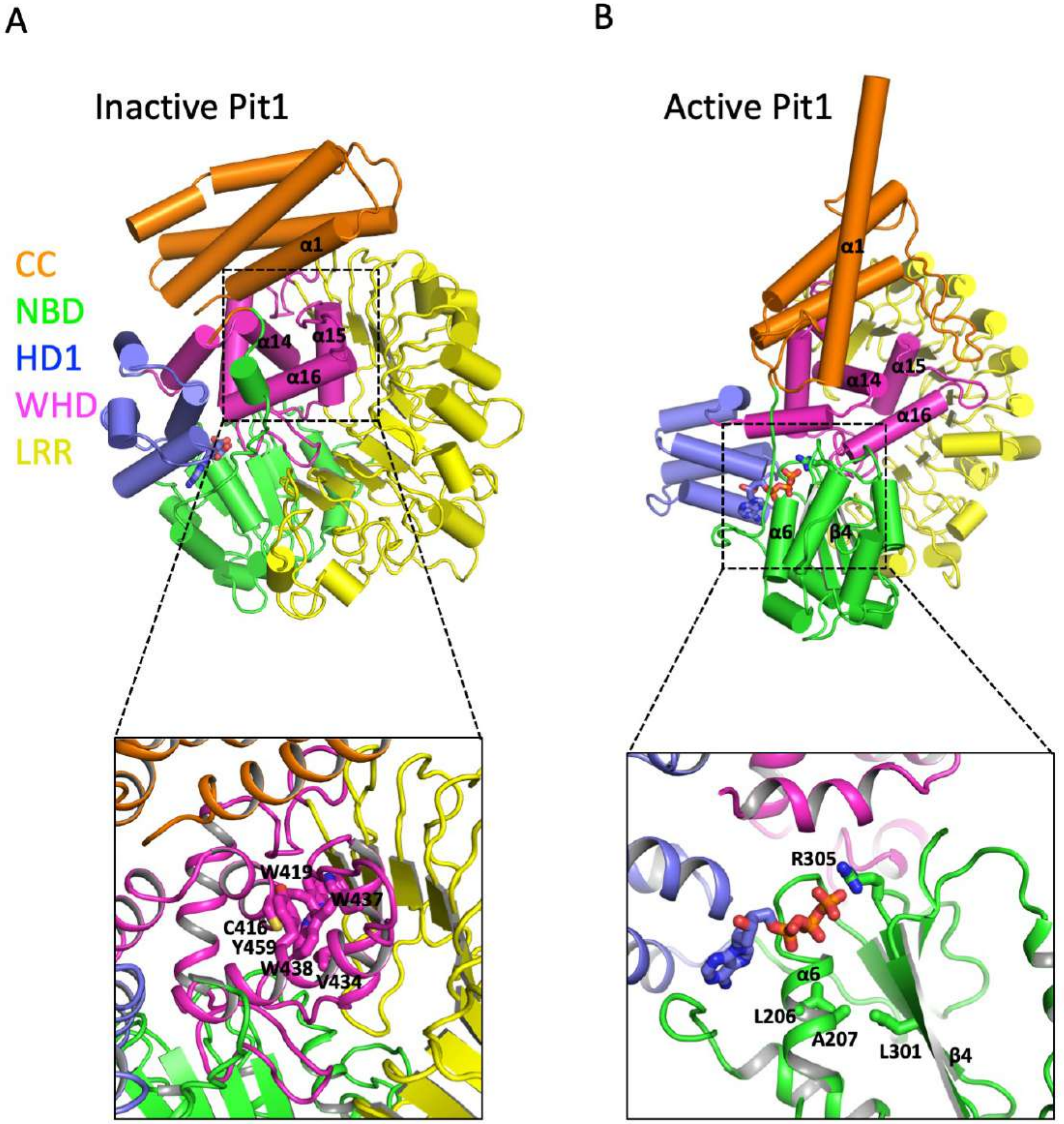

C

Pit1	1	M	G	T	V	L	D	A	L	A	W	K	F	L	E	K	L	G	Q	L	I	20
Pit2	1	M	G	T	V	L	D	A	L	A	W	K	F	L	E	K	L	G	Q	L	I	20
ZAR1	1	M	-	-	V	-	D	A	V	V	T	V	F	L	E	K	T	L	N	I	L	17

Supplementary Figure 16 Model structures of Pit1

(A) Ribbon representation of the model structure of Pit1 in its inactive (ADP-bound) form. The side chains of hydrophobic residues (C416, W419, V434, W437, W438 and Y459) which may be involved in stability among α14, α15 and α16 of WHD are shown in stick representation. ADP is shown as a stick model (red, oxygen; blue, nitrogen; orange, phosphorus).

(B) Ribbon representation of the model structure of Pit1 in its active (dATP-bound) form. The side chains of three key residues (A207, L206 and L301) which are thought to be involved in stability between α6 and β4 and the key residue for ATP binding (R305) are shown in stick representation. dATP is shown as a stick model (red, oxygen; blue, nitrogen; orange, phosphorus). These models of the Pit1 structures were produced by PyMOL based on the structures of ZAR1 (PDB: 6J5W and 6J6I).

(C) Multiple sequence alignment of α-helix 1 (α1) region among Pit1, Pit2 and ZAR1. The conserved hydrophobic and charged residues are highlighted in gray and green, respectively.

Supplementary Table 1

Function	Protein ID	Sample	MASCOT	emPAI	Identity to Pit1
NLR protein (Pit2)	LOC_Os01g05600.1	8h	565	0.4	88%
NLR protein [Pit1-associated NLR1 (PAN1)]	LOC_Os12g32680.1	8h/30h	31/35	0.03/0.03	23%
NLR protein [Pit1-associated NLR2 (PAN2)]	LOC_Os11g47780.1	8h	32	0.03	33%
NLR protein [Pit1-associated NLR3 (PAN3)]	LOC_Os11g28950.1	8h	31	0.03	32%
NLR protein [Pit1-associated NLR4 (PAN4)]	LOC_Os11g27430.1	30h	25	0.03	22%
NLR protein [Pit1-associated NLR5 (PAN5)]	gi|13872974	30h	30	0.13	30%
small GTPaseOsRac1	LOC_Os01g12900.1	30h	45	0.34	
small GTPase OsRac2	LOC_Os05g43820.1	30h	44	0.16	

Supplementary Table 2. The 60 pan-genome accessions used in this study

		Pit1			Pit2			Exon number		Length of protein (aa)		Length of genomic (bp)	
Subgroup	Accession	301	416	479	300	415	478	Pit1	Pit2	Pit1	Pit2	Pit1	Pit2
tropical japonica	GP39	L	C	G	P	F	W	1	4	989	875	2970	4275
	GP77	L	C	G	P	F	W	1	3	989	847	2970	4124
	GP536	L	C	G	P	F	W	1	3	989	847	2970	4124
	GP640	L	C	G	L	F	W	1	1	989	990	2970	2973
	GP761-1	L	C	G	P	F	W	1	3	989	847	2970	4124
temperate japonica	DHX2	L	C	G	L	C	W	1	1	989	990	2970	2973
	GP551	L	C	G	L	C	W	1	1	989	990	2970	2973
	GP567	L	C	G	P	F	W	1	4	989	875	2970	4275
	GP669	L	C	G	L	F	W	1	1	989	990	2970	2973
	GP677	L	C	G	L	F	W	1	1	989	990	2970	2973
	HP103	L	-	G	P	F	W	2	4	917	875	2969	4275
	HP13-2	L	-	G	P	F	W	2	4	917	875	2969	4275
	HP14	L	C	G	L	C	W	1	1	989	990	2970	2973
	HP38	L	-	G	P	F	W	1	1	989	883	2970	2652
	HP390	L	C	G	P	L	W	1	4	989	875	2970	4275
	HP44	L	C	G	L	F	W	1	1	989	989	2970	2970
	HP45	L	C	G	L	F	W	2	1	979	990	2970	2973
	HP48	L	C	G	L	C	W	1	1	989	990	2970	2973
	HP91-2	L	C	G	P	F	W	1	4	989	875	2970	4275
	HP98	L	C	G	L	C	W	1	1	989	990	2970	2973
	IL9	L	C	G	L	C	W	1	1	989	990	2970	2973
	Koshihikari	L	C	G	L	C	W	1	1	989	990	2970	2973
	KY131	L	C	G	L	C	W	1	1	989	990	2970	2973
	LG31	L	C	G	L	C	W	1	1	989	990	2970	2973
	UR28	L	C	G	P	F	W	1	4	989	875	2970	4275
	WYG7	L	C	G	P	L	W	1	4	989	875	2970	4275
	Nipponbare	L	C	G	L	F	W	1	1	989	990	2970	2973
O. sativa aus	GP62	L	C	W	P	F	W	1	2	988	836	2967	2511
	GP104	L	C	W	P	F	W	1	1	988	990	2967	2973
	GP124	L	C	W	P	F	W	1	1	988	990	2967	2973
	GP540	L	C	W	L	F	W	1	2	988	978	2967	2972
	Kasalath	L	C	W	P	F	W	1	1	988	990	2967	2973
	W3105-1	L	C	W	P	F	W	1	2	988	971	2967	2971
indica	GLA4	L	-	G	P	F	W	2	4	917	875	2969	4275
	GP22	L	C	G	P	L	W	1	3	989	866	2970	3474
	GP3	L	-	G	P	F	W	2	4	917	875	2969	4274
	GP51	L	C	W	L	F	W	1	2	988	890	2967	2972
	GP772-1	L	C	G	P	L	W	1	4	989	875	2970	4275
	HP119	L	C	G	P	L	W	1	4	989	875	2970	4275
	HP263	L	C	G	P	L	W	1	3	989	847	2970	4124
	HP274	L	C	G	P	L	W	1	3	989	866	2970	3474
	HP327	L	C	G	P	L	W	1	4	989	875	2970	4275

	HP362-2	L	C	G	P	L	W	1	3	989	847	2970	4124
	HP383	L	C	W	L	F	W	1	2	988	978	2967	2972
	HP396	L	-	G	P	F	W	2	4	917	875	2969	4275
	HP407	L	C	G	P	L	W	1	4	989	875	2970	4275
	HP486	L	C	G	P	F	W	2	4	960	875	2969	4275
	HP492	L	C	G	P	L	W	1	4	989	875	2970	4275
	HP517-1	L	C	G	P	L	W	1	4	989	875	2970	4275
	HP577	L	-	G	P	F	W	2	4	917	875	2969	4275
	W0128	L	C	G	P	F	W	1	1	989	990	2970	2973
	K59	L	C	G	P	F	W	1	1	989	990	2970	2973
aromatic	GP295	L	C	G	L	F	W	2	1	928	990	2968	2973
	W0123-1	L	C	G	P	F	W	2	4	951	875	2953	4275
O.rufupogon	W0141	L	C	G	L	C	W	1	3	989	799	2970	2970
	W0170	L	C	G	L	-	W	1	3	989	796	2970	2949
	W1687	L	C	G	L	C	W	1	1	989	990	2970	2973
	W1698	L	C	W	L	F	W	2	2	969	890	2965	2972
	W1777	L	C	G	L	C	W	1	2	989	979	2970	2973
	W2012	L	C	G	L	C	W	1	1	989	990	2970	2973

Pit1 and Pit2 sequences are provided as a Source Data file.

Supplementary Table 3. Nucleotide polymorphism of *Pit1* and *Pit2* alleles in different rice groups

cDNA	Subgroup	Region	location (nt)	*n*	*s*	*π*	*θ*	Tajima's *D*	D*	F*
Pit1	*O. sativa*	Total	1-2970	53	122	0.01269	0.01082	0.61138	2.1685	1.88788*
		CC	1-420	53	4	0.00308	0.0021	1.04657	0.99874	1.18412
		NB-ARC	541-1560	53	30	0.01359	0.01238	0.32321	1.84187	1.54492**
		LRR	1561-2970	53	84	0.01544	0.01313	0.61773	2.10926**	1.85212*
	O. sativa japonica	Total	1-2970	29	9	0.00106	0.00085	0.80985	1.37016	1.4014
		CC	1-420	29	1	0.00122	0.00062	1.57677	0.60727	1.00242
		NB-ARC	541-1560	29	0	0	0	not detected	0	0
		LRR	1561-2970	29	6	0.00099	0.0011	-0.31318	1.22274	0.89582
	O. sativa indica	Total	1-2970	19	118	0.00917	0.01265	-1.14508	1.72245**	1.0094
		CC	1-420	19	4	0.00226	0.00272	-0.50708	1.11537	0.77044
		NB-ARC	541-1560	19	30	0.00817	0.01194	-1.25306	1.46578*	0.77596
		LRR	1561-2970	19	82	0.01224	0.01664	-1.09197	1.75173**	1.05412
Pit2	*O. sativa*	Total	1-2973	53	54	0.01048	0.00577	2.81241**	2.01466**	2.75697**
		CC	1-420	53	1	0.00164	0.00101	0.82093	0.53819	0.71741
		NB-ARC	541-1560	53	11	0.00325	0.00248	0.88684	1.43912	1.48182
		LRR	1561-2973	53	42	0.02083	0.01065	3.2381***	1.94751**	2.88437**
	O. sativa japonica	Total	1-2973	29	117	0.02164	0.01195	3.10493***	1.88622**	2.69313**
		CC	1-420	29	2	0.00068	0.00124	-0.95426	0.82034	0.37431
		NB-ARC	541-1560	29	10	0.00351	0.00254	1.23017	1.40659*	1.5795
		LRR	1561-2973	29	104	0.05196	0.0275	3.39003***	1.87765**	2.79462**
	O. sativa indica	Total	1-2973	19	98	0.01582	0.01262	0.5698	1.76781**	1.64284*
		CC	1-420	19	1	0.00185	0.00101	1.54705	0.65779	1.01906
		NB-ARC	541-1560	19	5	0.00191	0.00147	0.93278	1.19239	1.29084
		LRR	1561-2973	19	92	0.03408	0.02739	0.50738	1.76479**	1.61877*

n: number of samples; *S*: number of polymorphic or segregating sites; π: nucleotide diversity; *θ*: Watterson's nucleotide diversity estimator based on silent site; *, ** and *** for Tajima's *D*, *D** and *F** indicate $P < 0.05$, $P < 0.01$ and $P < 0.001$, respectively.

Supplementary Table 4. Primers used in this study

Primer name	Sequence (5'-3')
**Primers for Cloning & Sequence**	
Pit1 CC-F	CACCATGGGCACAGTTTTGGATGC
Pit1 CC-R	TCTCTCCAAGCCAAACATCT
Pit2 CC-F	CACCATGGGCACAGTTTTGGATGC
Pit2 CC-R	TCTCTCGAAGCCAAACATCT
Pit1 NB-ARC-F	CACCATGACCAACCGGCAACAAAT
Pit1 NB-ARC-R	ATTGCTGATACATAAGTGGC
Pit2 NB-ARC-F	CACCATGACCAACCGGCAACAAGT
Pit2 NB-ARC-R	ATTGCTTATTACTAAGTGAC
Pit1 LRR-F	CACCATGGATGTAGAAGAAATACC
Pit1 LRR-R	ATTATAGAGAGATTCATCTG
Pit2 LRR-F	CACCATGGATGTAAAAGAAATACC
Pit2 LRR-R	ATTATAGAGAGTTTCATCTG
Pit1-promoter^{K59}-F	CACCAGAAGAGATCAAATGCTTAA
Pit2-promoter^{K59}-F	CACCTGACGGCCTTGGTGCCCTCG
Pit2 Transient-RNAi UTR-F	CACCACTTGGACTTTGCAACAAATG
Pit2 Transient-RNAi UTR-R	TCACCAGAGTGCAGACAGCAG
**Primers for CRISPR-KO-plants**	
KO Pit1 CRISPR-1 F	GTTGGAACAAAGAGATGTTTGGCT
KO Pit1 CRISPR-1 R	AAACAGCCAAACATCTCTTTGTTC
KO Pit1 CRISPR-2 F	GTTGTCCTGTAGATGAACTTGAGG
KO Pit1 CRISPR-2 R	AAACCCTCAAGTTCATCTACAGGA
KO Pit2 CRISPR-1 F	TGTGTGTTTGATGTCGATGACATCG
KO Pit2 CRISPR-1 R	AAACCGATGTCATCGACATCAAACA
KO Pit2 CRISPR-2 F	TGTGTGAAAGTTTTCATTTGATCAC
KO Pit2 CRISPR-2 R	AAACGTGATCAAATGAAAACTTTCA
**Primers for RT-qPCR and RT-PCR**	
M. g. Pot2-qPCR-F	ACGACCCGTCTTTACTTATTTGG
M. g. Pot2-qPCR-R	AAGTAGCGTTGGTTTTGTTGGAT
Ubiquitin-qPCR-F	AACCAGCTGAGGCCCAAGA
Ubiquitin-qPCR-R	ACGATTGATTTAACCAGTCCATGA
Pit OX-F	CTGCACTTTGAATACCATTGGC
Pit OX-R	GGAGAATTTCCAATCTCTGTAATCTAA
Ubiquitin-F	CCAGGACAAGATGATCTGCC
Ubiquitin-R	AAGAAGCTGAAGCATCCAGC
Pit1-qPCR-F	GACTGCACTTCATGTTCAAT
Pit1-qPCR-R	ACTATTGCATCTGCGCCTTTA
Pit2-qPCR-F	GAGTGCATTTCATGCTCTGA
Pit2-qPCR-R	ACTACTGCATCTGCACATCTG
**Primers for mutagenesis**	
Pit1DT(p2type 860-910)1F	AGAGGTTTGAATTCTCGTATCCCTGTGACCTCAAGAAACCTGGATGTTTTGGTTG

Pit1DT(p2type 860-910)1R	TCACAGGGATACGAGAATTCAAACCTCTCTCGGAAGGCGATCGAAGAAGATCAATC
Pit1DT(p2type 960-1032)2F	GGCCTAGAACTGCTGATGAAGATGTCATTAGGGCCATACGAGCAAAGAAGAGAATTCAG
Pit1DT(p2type 960-1032)2R	CTTCATCAGCAGTTCTAGGCCATCGTAATCATTCATTTTGTTTACCCTGTGGGTATAAG
Pit1DT(p2type 1122-1188)3F	AGAGCATCCGAGATAGCAAATGGTCTATTCATGGACTTCCCAAAGAACTAGGAG
Pit1DT(p2type 1122-1188)3R	CCATTTGCTATCTCGGATGCTCTCCCATTCTGCCTTTGTTTTCTTACTAGATAG
Pit1DT(p2type 1248-1290)4F	GTGCTTTGTTACCATCAAATTTTGGGATCCATCGTGATGCTGTCGCTTACTGGTGG
Pit1DT(p2type 1248-1290)4R	CACGATGGATCCCAAAATTTGATGGTAACAAAGCACACCAGAGAAAGAATTGTTTAAG
Pit1DT(p2type 1336-1398)5F	TACATGAAGCCGCAGAAGAATACTACCATGAGCTAATTAGAATGAATCTTCTGCAGCC
Pit1DT(p2type 1336-1398)5R	TAGTATTCTTCTGCGGCTTCATGTATTGAGTACCCATGCATTTTCGTCACGAAACCTTC
Pit1DT(p2type 1398-1440)6F	GCAGCCAAAACCAGAGTTTGTTGACAAATGGATGTCAACAATGCATGTTCTATTGAG
Pit1DT(p2type 1398-1440)6R	GTTGACATCCATTTGTCAACAAACTCTGGTTTTGGCTGCAGAAGATTCATTCTGATTAG
Pit1DT(p2type 1500-1551)7F	GAAAATAGCAAAGCCTTACTGAATCTACGTCACTTAGTAATCAGCAATGATGTA
Pit1DT(p2type 1500-1551)7R	GTGACGTAGATTCAGTAAGGCTTTGCTATTTTCCATATTCATGAATAAGGAGTGATC
Pit2DT(p1type 860-910)1F	CTTCGTTTGCCTTTCTTGAGAGGTTTGAATTCTCATATCCTTGTGACCTCAAGAAACCT
Pit2DT(p1type 860-910)1R	GTCACAAGGATATGAGAATTCAAACCTCTCAAGAAAGGCAAACGAAGAAGATCAATCCA
Pit2DT(p1type 960-1032)2F	GGCCTAGAACTGCTGATGAAGATGTCATTAGGGCCATACGAGCAAAGCAGAGAATTCAG
Pit2DT(p1type 960-1032)2R	CTTCATCAGCAGTTCTAGGCCATCGCAATCATTCATTTTGTTTACCTTGTGGGTATAAG
Pit2DT(p1type 1122-1188)3F	AGAGTATCCGAGATAGCAAGTGGTCCATTCATGGACTTCCCAGAGAACTAGGAG
Pit2DT(p1type 1122-1188)3R	CCACTTGCTATCTCGGATACTCTCCCATTCTGCCCTTGTTCTTTTAGTAGATAG
Pit2DT(p1type 1248-1290)4F	GTGCTTTGTTGCCGTCAAATTTTGTGATACGTCGTGATGCTGTCGCTTACTGGTGG
Pit2DT(p1type 1248-1290)4F	TCACGACGTATCACAAAATTTGACGGCAACAAAGCACACCAGAGAAAGCATTGCTTAAG
Pit2DT(p1type 1336-1398)5F	ATACATGAGGTTGCTGAAGAGTACTACCATGAGCTAATCAGAAGGAATCTTCTGCAGC
Pit2DT(p1type 1336-1398)5R	ATGGTAGTACTCTTCAGCAACCTCATGTATTGAGTACCCATGCACTTCCGTCACAAAGC
Pit2DT(p1type 1398-1440)6F	GCAGCCACGACCAGAGTTTGTAGACAAAGGCGAGTCAACGATGCATGTTCTTTTGAG
Pit2DT(p1type 1398-1440)6R	GTTGACTCGCCTTTGTCTACAAACTCTGGTCGTGGCTGCAGAAGATTCCTTCTAATTAG
Pit2DT(p1type 1500-1551)7F	GAATATAGCAAAGCCTTACCAAATCTACGCCACTTATGTATAAGCAATGATGTA
Pit2DT(p1type 1500-1551)7R	GTGGCGTAGATTTGGTAAGGCTTTGCTATATTCCATGTTCATGAATATGGAATGATC
Pit1- L290S F	ATCTTCTTCGTTCGCCTTTCTTGAGAGGTTTG
Pit1- L290S R	TCTCAAGAAAGGCGAACGAAGAAGATCAATCC
Pit1- F292S F	TCGTTTGCCTTCCTTGAGAGGTTTGAATTCTC
Pit1- F292S R	AACCTCTCAAGGAAGGCAAACGAAGAAGATC
Pit1- L293E F	GTTTGCCTTTCGAGAGAGGTTTGAATTCTC
Pit1- L293E R	TCAAACCTCTCTCGAAAGGCAAACGAAGAAG
Pit1- H299R F	AGGTTTGAATTCTCGTATCCTTGTGACCTC
Pit1- H299R R	TCACAAGGATACGAGAATTCAAACCTCTC
Pit1 L301P F	GAATTCTCATATCCCTGTGACCTCAAGAAACC
Pit1 L301P R	GTTTCTTGAGGTCACAGGGATATGAGAATTCAAACC
Pit1 C416F F	CTGAACTTAAACAATTCTTTCTCTGGTGTGCTTTGTTG
Pit1 C416F R	AGCACACCAGAGAAAGAATTGTTTAAGTTCAGGGGGTA
Pit1 V428G F	TGCCGTCAAATTTTGGGATACGTCGTGATGCTGTCGC
Pit1 V428G R	AGCATCACGACGTATCCCAAAATTTGACGGCAACAAAG
Pit1 R430H F	TCAAATTTTGTGATACATCGTGATGCTGTCGCTTACTG
Pit1 R430H R	AAGCGACAGCATCACGATGTATCACAAAATTTGACGGC
Pit1 V455A F	TACTCAATACATGAGGCCGCTGAAGAGTACTACCATGAGCTAATC
Pit1 V455A R	TGGTAGTACTCTTCAGCGGCCTCATGTATTGAGTACCCATGCACT

Pit1 E446K F	AGGTTTCGTGACGAAAGTGCATGGGTACTCAATACATG
Pit1 E446K R	AGTACCCATGCACTTTCGTCACGAAACCTTCAGCAACC
Pit1 V447M F	AGGTTTCGTGACGGAAATGCATGGGTACTCAATACATGAG
Pit1 V447M R	TATTGAGTACCCATGCATTTCCGTCACGAAACCTTCAGCAAC
Pit1 R466M F	ATGAGCTAATCAGAATGAATCTTCTGCAGCCACGAC
Pit1 R466M R	TGGCTGCAGAAGATTCATTCTGATTAGCTCATGGTAG
Pit1 R472K F	AGGAATCTTCTGCAGCCAAAACCAGAGTTTGTAGACAAAGGCGAG
Pit1 R472K R	TGTCTACAAACTCTGGTTTTGGCTGCAGAAGATTCCTTCTGATTAG
Pit1 G479W F	AGTTTGTAGACAAATGGGAGTCAACAATGCATGTTCTATTGAGGTC
Pit1 G479W R	AACATGCATTGTTGACTCCCATTTGTCTACAAACTCTGGTCGTGGC
Pit1 E480M F	AGAGTTTGTAGACAAAGGCATGTCAACAATGCATGTTCTATTGAGGTC
Pit1 E480M R	ATGCATTGTTGACATGCCTTTGTCTACAAACTCTGGTCGTGGCTGCAG
Pit2 P300L F	GGCTTGAATTCTCGTATCCTTGTGACCTCAAGAAACCT
Pit2 P300L R	TTTCTTGAGGTCACAAGGATACGAGAATTCAAGCCTCT
Pit2 F415C F	TGAACTTAAGCAATGCTTTCTCTGGTGTGCTTTGTTAC
Pit2 F415C R	AGCACACCAGAGAAAGCATTGCTTAAGTTCAGGAGGTA
Pit2 W478G F	AGAGTTTGTTGACAAAGGCATGTCAACGATGCATGTTCTTTTGAG
Pit2 W478G R	ATGCATCGTTGACATGCCTTTGTCAACAAACTCTGGTTTTGGCTGC

Supplementary Table 5: The Ka/Ks ratios for Pit1 genes in O.sativa.

Gene 1	Gene 2	Ks	Ka	Ka/Ks
K59_Pit1	Nipponbare_Pit1	0.0036	0.002	0.555556
K59_Pit1	GP39_Pit1	0.0036	0.001	0.277778
K59_Pit1	GP77_Pit1	0.0036	0.001	0.277778
K59_Pit1	GP536_Pit1	0.0036	0.001	0.277778
K59_Pit1	GP640_Pit1	0.0036	0.002	0.555556
K59_Pit1	GP761-1_Pit1	0.0036	0.001	0.277778
K59_Pit1	DHX2_Pit1	0.0036	0.002	0.555556
K59_Pit1	GP551_Pit1	0.0036	0.002	0.555556
K59_Pit1	GP567_Pit1	0.0036	0.001	0.277778
K59_Pit1	GP669_Pit1	0.0036	0.002	0.555556
K59_Pit1	GP677_Pit1	0.0036	0.002	0.555556
K59_Pit1	HP13-2_Pit1	0.0073	0.0015	0.205479
K59_Pit1	HP14_Pit1	0.0036	0.002	0.555556
K59_Pit1	HP38_Pit1	0.0036	0.001	0.277778
K59_Pit1	HP44_Pit1	0.0036	0.002	0.555556
K59_Pit1	HP45_Pit1	0.0036	0.002	0.555556
K59_Pit1	HP48_Pit1	0.0036	0.002	0.555556
K59_Pit1	HP91-2_Pit1	0.0036	0.001	0.277778
K59_Pit1	HP98_Pit1	0.0036	0.002	0.555556
K59_Pit1	HP103_Pit1	0.0073	0.0015	0.205479
K59_Pit1	HP390_Pit1	0.0036	0.0015	0.416667
K59_Pit1	IL9_Pit1	0.0036	0.002	0.555556
K59_Pit1	Koshihikar_Pit1	0.0036	0.002	0.555556
K59_Pit1	KY131_Pit1	0.0036	0.002	0.555556
K59_Pit1	LG31_Pit1	0.0036	0.002	0.555556
K59_Pit1	UR28_Pit1	0.0036	0.001	0.277778
K59_Pit1	WYG7_Pit1	0.0036	0.0015	0.416667
K59_Pit1	GLA4_Pit1	0.0073	0.0015	0.205479
K59_Pit1	GP3_Pit1	0.0073	0.0015	0.205479
K59_Pit1	GP22_Pit1	0.0036	0.0015	0.416667
K59_Pit1	GP51_Pit1	0.0992	0.0336	0.33871
K59_Pit1	GP772-1_Pit1	0.0036	0.0015	0.416667
K59_Pit1	HP119_Pit1	0.0036	0.0015	0.416667
K59_Pit1	HP263_Pit1	0.0036	0.0015	0.416667
K59_Pit1	HP274_Pit1	0.0036	0.0015	0.416667
K59_Pit1	HP327_Pit1	0.0036	0.0015	0.416667
K59_Pit1	HP362-2_Pit1	0.0036	0.0015	0.416667
K59_Pit1	HP383_Pit1	0.0992	0.0336	0.33871
K59_Pit1	HP396_Pit1	0.0073	0.0015	0.205479
K59_Pit1	HP407_Pit1	0.0036	0.0015	0.416667
K59_Pit1	HP492_Pit1	0.0036	0.0015	0.416667
K59_Pit1	HP517-1_Pit1	0.0036	0.0015	0.416667
K59_Pit1	HP577_Pit1	0.0073	0.0015	0.205479
K59_Pit1	W0128_Pit1	0	0	
K59_Pit1	GP104_Pit1	0.0972	0.0336	0.345679
K59_Pit1	GP124_Pit1	0.0972	0.0336	0.345679
K59_Pit1	GP540_Pit1	0.0992	0.0336	0.33871
K59_Pit1	Kasalath_Pit1	0.0972	0.0336	0.345679
K59_Pit1	W3105-1_Pit1	0.0972	0.0336	0.345679
K59_Pit1	GP295-1_Pit1	0.0036	0.002	0.555556
K59_Pit1	HP486_Pit1	0.0178	0.0104	0.58427
K59_Pit1	GP62_Pit1	0.0972	0.0336	0.345679
Nipponbare_Pit1	GP39_Pit1	0	0.002	
Nipponbare_Pit1	GP77_Pit1	0	0.002	

Nipponbare_Pit1	GP536_Pit1	0	0.002	
Nipponbare_Pit1	GP640_Pit1	0	0	
Nipponbare_Pit1	GP761-1_Pit1	0	0.002	
Nipponbare_Pit1	DHX2_Pit1	0	0.001	
Nipponbare_Pit1	GP551_Pit1	0	0.001	
Nipponbare_Pit1	GP567_Pit1	0	0.002	
Nipponbare_Pit1	GP669_Pit1	0	0	
Nipponbare_Pit1	GP677_Pit1	0	0	
Nipponbare_Pit1	HP13-2_Pit1	0.0036	0.0026	0.722222
Nipponbare_Pit1	HP14_Pit1	0	0.001	
Nipponbare_Pit1	HP38_Pit1	0	0.002	
Nipponbare_Pit1	HP44_Pit1	0	0	
Nipponbare_Pit1	HP45_Pit1	0	0	
Nipponbare_Pit1	HP48_Pit1	0	0.001	
Nipponbare_Pit1	HP91-2_Pit1	0	0.002	
Nipponbare_Pit1	HP98_Pit1	0	0.001	
Nipponbare_Pit1	HP103_Pit1	0.0036	0.0026	0.722222
Nipponbare_Pit1	HP390_Pit1	0	0.0026	
Nipponbare_Pit1	IL9_Pit1	0	0.001	
Nipponbare_Pit1	Koshihikar_Pit1	0	0.001	
Nipponbare_Pit1	KY131_Pit1	0	0.001	
Nipponbare_Pit1	LG31_Pit1	0	0.001	
Nipponbare_Pit1	UR28_Pit1	0	0.002	
Nipponbare_Pit1	WYG7_Pit1	0	0.0026	
Nipponbare_Pit1	GLA4_Pit1	0.0036	0.0026	0.722222
Nipponbare_Pit1	GP3_Pit1	0.0036	0.0026	0.722222
Nipponbare_Pit1	GP22_Pit1	0	0.0026	
Nipponbare_Pit1	GP51_Pit1	0.095	0.0347	0.365263
Nipponbare_Pit1	GP772-1_Pit1	0	0.0026	
Nipponbare_Pit1	HP119_Pit1	0	0.0026	
Nipponbare_Pit1	HP263_Pit1	0	0.0026	
Nipponbare_Pit1	HP274_Pit1	0	0.0026	
Nipponbare_Pit1	HP327_Pit1	0	0.0026	
Nipponbare_Pit1	HP362-2_Pit1	0	0.0026	
Nipponbare_Pit1	HP383_Pit1	0.095	0.0347	0.365263
Nipponbare_Pit1	HP396_Pit1	0.0036	0.0026	0.722222
Nipponbare_Pit1	HP407_Pit1	0	0.0026	
Nipponbare_Pit1	HP492_Pit1	0	0.0026	
Nipponbare_Pit1	HP517-1_Pit1	0	0.0026	
Nipponbare_Pit1	HP577_Pit1	0.0036	0.0026	0.722222
Nipponbare_Pit1	W0128_Pit1	0.0036	0.002	0.555556
Nipponbare_Pit1	GP104_Pit1	0.0929	0.0347	0.37352
Nipponbare_Pit1	GP124_Pit1	0.0929	0.0347	0.37352
Nipponbare_Pit1	GP540_Pit1	0.095	0.0347	0.365263
Nipponbare_Pit1	Kasalath_Pit1	0.0929	0.0347	0.37352
Nipponbare_Pit1	W3105-1_Pit1	0.0929	0.0347	0.37352
Nipponbare_Pit1	GP295-1_Pit1	0	0	
Nipponbare_Pit1	HP486_Pit1	0.0141	0.0114	0.808511
Nipponbare_Pit1	GP62_Pit1	0.0929	0.0347	0.37352
GP39_Pit1	GP77_Pit1	0	0	
GP39_Pit1	GP536_Pit1	0	0	
GP39_Pit1	GP640_Pit1	0	0.002	
GP39_Pit1	GP761-1_Pit1	0	0	
GP39_Pit1	DHX2_Pit1	0	0.002	
GP39_Pit1	GP551_Pit1	0	0.002	
GP39_Pit1	GP567_Pit1	0	0	

GP39_Pit1	GP669_Pit1	0	0.002	
GP39_Pit1	GP677_Pit1	0	0.002	
GP39_Pit1	HP13-2_Pit1	0.0036	0.0005	0.138889
GP39_Pit1	HP14_Pit1	0	0.002	
GP39_Pit1	HP38_Pit1	0	0	
GP39_Pit1	HP44_Pit1	0	0.002	
GP39_Pit1	HP45_Pit1	0	0.002	
GP39_Pit1	HP48_Pit1	0	0.002	
GP39_Pit1	HP91-2_Pit1	0	0	
GP39_Pit1	HP98_Pit1	0	0.002	
GP39_Pit1	HP103_Pit1	0.0036	0.0005	0.138889
GP39_Pit1	HP390_Pit1	0	0.0005	
GP39_Pit1	IL9_Pit1	0	0.002	
GP39_Pit1	Koshihikar_Pit1	0	0.002	
GP39_Pit1	KY131_Pit1	0	0.002	
GP39_Pit1	LG31_Pit1	0	0.002	
GP39_Pit1	UR28_Pit1	0	0	
GP39_Pit1	WYG7_Pit1	0	0.0005	
GP39_Pit1	GLA4_Pit1	0.0036	0.0005	0.138889
GP39_Pit1	GP3_Pit1	0.0036	0.0005	0.138889
GP39_Pit1	GP22_Pit1	0	0.0005	
GP39_Pit1	GP51_Pit1	0.095	0.0336	0.353684
GP39_Pit1	GP772-1_Pit1	0	0.0005	
GP39_Pit1	HP119_Pit1	0	0.0005	
GP39_Pit1	HP263_Pit1	0	0.0005	
GP39_Pit1	HP274_Pit1	0	0.0005	
GP39_Pit1	HP327_Pit1	0	0.0005	
GP39_Pit1	HP362-2_Pit1	0	0.0005	
GP39_Pit1	HP383_Pit1	0.095	0.0336	0.353684
GP39_Pit1	HP396_Pit1	0.0036	0.0005	0.138889
GP39_Pit1	HP407_Pit1	0	0.0005	
GP39_Pit1	HP492_Pit1	0	0.0005	
GP39_Pit1	HP517-1_Pit1	0	0.0005	
GP39_Pit1	HP577_Pit1	0.0036	0.0005	0.138889
GP39_Pit1	W0128_Pit1	0.0036	0.001	0.277778
GP39_Pit1	GP104_Pit1	0.0929	0.0336	0.361679
GP39_Pit1	GP124_Pit1	0.0929	0.0336	0.361679
GP39_Pit1	GP540_Pit1	0.095	0.0336	0.353684
GP39_Pit1	Kasalath_Pit1	0.0929	0.0336	0.361679
GP39_Pit1	W3105-1_Pit1	0.0929	0.0336	0.361679
GP39_Pit1	GP295-1_Pit1	0	0.002	
GP39_Pit1	HP486_Pit1	0.0141	0.0094	0.666667
GP39_Pit1	GP62_Pit1	0.0929	0.0336	0.361679
GP77_Pit1	GP536_Pit1	0	0	
GP77_Pit1	GP640_Pit1	0	0.002	
GP77_Pit1	GP761-1_Pit1	0	0	
GP77_Pit1	DHX2_Pit1	0	0.002	
GP77_Pit1	GP551_Pit1	0	0.002	
GP77_Pit1	GP567_Pit1	0	0	
GP77_Pit1	GP669_Pit1	0	0.002	
GP77_Pit1	GP677_Pit1	0	0.002	
GP77_Pit1	HP13-2_Pit1	0.0036	0.0005	0.138889
GP77_Pit1	HP14_Pit1	0	0.002	
GP77_Pit1	HP38_Pit1	0	0	
GP77_Pit1	HP44_Pit1	0	0.002	
GP77_Pit1	HP45_Pit1	0	0.002	

GP77_Pit1	HP48_Pit1	0	0.002	
GP77_Pit1	HP91-2_Pit1	0	0	
GP77_Pit1	HP98_Pit1	0	0.002	
GP77_Pit1	HP103_Pit1	0.0036	0.0005	0.138889
GP77_Pit1	HP390_Pit1	0	0.0005	
GP77_Pit1	IL9_Pit1	0	0.002	
GP77_Pit1	Koshihikar_Pit1	0	0.002	
GP77_Pit1	KY131_Pit1	0	0.002	
GP77_Pit1	LG31_Pit1	0	0.002	
GP77_Pit1	UR28_Pit1	0	0	
GP77_Pit1	WYG7_Pit1	0	0.0005	
GP77_Pit1	GLA4_Pit1	0.0036	0.0005	0.138889
GP77_Pit1	GP3_Pit1	0.0036	0.0005	0.138889
GP77_Pit1	GP22_Pit1	0	0.0005	
GP77_Pit1	GP51_Pit1	0.095	0.0336	0.353684
GP77_Pit1	GP772-1_Pit1	0	0.0005	
GP77_Pit1	HP119_Pit1	0	0.0005	
GP77_Pit1	HP263_Pit1	0	0.0005	
GP77_Pit1	HP274_Pit1	0	0.0005	
GP77_Pit1	HP327_Pit1	0	0.0005	
GP77_Pit1	HP362-2_Pit1	0	0.0005	
GP77_Pit1	HP383_Pit1	0.095	0.0336	0.353684
GP77_Pit1	HP396_Pit1	0.0036	0.0005	0.138889
GP77_Pit1	HP407_Pit1	0	0.0005	
GP77_Pit1	HP492_Pit1	0	0.0005	
GP77_Pit1	HP517-1_Pit1	0	0.0005	
GP77_Pit1	HP577_Pit1	0.0036	0.0005	0.138889
GP77_Pit1	W0128_Pit1	0.0036	0.001	0.277778
GP77_Pit1	GP104_Pit1	0.0929	0.0336	0.361679
GP77_Pit1	GP124_Pit1	0.0929	0.0336	0.361679
GP77_Pit1	GP540_Pit1	0.095	0.0336	0.353684
GP77_Pit1	Kasalath_Pit1	0.0929	0.0336	0.361679
GP77_Pit1	W3105-1_Pit1	0.0929	0.0336	0.361679
GP77_Pit1	GP295-1_Pit1	0	0.002	
GP77_Pit1	HP486_Pit1	0.0141	0.0094	0.666667
GP77_Pit1	GP62_Pit1	0.0929	0.0336	0.361679
GP536_Pit1	GP640_Pit1	0	0.002	
GP536_Pit1	GP761-1_Pit1	0	0	
GP536_Pit1	DHX2_Pit1	0	0.002	
GP536_Pit1	GP551_Pit1	0	0.002	
GP536_Pit1	GP567_Pit1	0	0	
GP536_Pit1	GP669_Pit1	0	0.002	
GP536_Pit1	GP677_Pit1	0	0.002	
GP536_Pit1	HP13-2_Pit1	0.0036	0.0005	0.138889
GP536_Pit1	HP14_Pit1	0	0.002	
GP536_Pit1	HP38_Pit1	0	0	
GP536_Pit1	HP44_Pit1	0	0.002	
GP536_Pit1	HP45_Pit1	0	0.002	
GP536_Pit1	HP48_Pit1	0	0.002	
GP536_Pit1	HP91-2_Pit1	0	0	
GP536_Pit1	HP98_Pit1	0	0.002	
GP536_Pit1	HP103_Pit1	0.0036	0.0005	0.138889
GP536_Pit1	HP390_Pit1	0	0.0005	
GP536_Pit1	IL9_Pit1	0	0.002	
GP536_Pit1	Koshihikar_Pit1	0	0.002	
GP536_Pit1	KY131_Pit1	0	0.002	

GP536_Pit1	LG31_Pit1	0	0.002	
GP536_Pit1	UR28_Pit1	0	0	
GP536_Pit1	WYG7_Pit1	0	0.0005	
GP536_Pit1	GLA4_Pit1	0.0036	0.0005	0.138889
GP536_Pit1	GP3_Pit1	0.0036	0.0005	0.138889
GP536_Pit1	GP22_Pit1	0	0.0005	
GP536_Pit1	GP51_Pit1	0.095	0.0336	0.353684
GP536_Pit1	GP772-1_Pit1	0	0.0005	
GP536_Pit1	HP119_Pit1	0	0.0005	
GP536_Pit1	HP263_Pit1	0	0.0005	
GP536_Pit1	HP274_Pit1	0	0.0005	
GP536_Pit1	HP327_Pit1	0	0.0005	
GP536_Pit1	HP362-2_Pit1	0	0.0005	
GP536_Pit1	HP383_Pit1	0.095	0.0336	0.353684
GP536_Pit1	HP396_Pit1	0.0036	0.0005	0.138889
GP536_Pit1	HP407_Pit1	0	0.0005	
GP536_Pit1	HP492_Pit1	0	0.0005	
GP536_Pit1	HP517-1_Pit1	0	0.0005	
GP536_Pit1	HP577_Pit1	0.0036	0.0005	0.138889
GP536_Pit1	W0128_Pit1	0.0036	0.001	0.277778
GP536_Pit1	GP104_Pit1	0.0929	0.0336	0.361679
GP536_Pit1	GP124_Pit1	0.0929	0.0336	0.361679
GP536_Pit1	GP540_Pit1	0.095	0.0336	0.353684
GP536_Pit1	Kasalath_Pit1	0.0929	0.0336	0.361679
GP536_Pit1	W3105-1_Pit1	0.0929	0.0336	0.361679
GP536_Pit1	GP295-1_Pit1	0	0.002	
GP536_Pit1	HP486_Pit1	0.0141	0.0094	0.666667
GP536_Pit1	GP62_Pit1	0.0929	0.0336	0.361679
GP640_Pit1	GP761-1_Pit1	0	0.002	
GP640_Pit1	DHX2_Pit1	0	0.001	
GP640_Pit1	GP551_Pit1	0	0.001	
GP640_Pit1	GP567_Pit1	0	0.002	
GP640_Pit1	GP669_Pit1	0	0	
GP640_Pit1	GP677_Pit1	0	0	
GP640_Pit1	HP13-2_Pit1	0.0036	0.0026	0.722222
GP640_Pit1	HP14_Pit1	0	0.001	
GP640_Pit1	HP38_Pit1	0	0.002	
GP640_Pit1	HP44_Pit1	0	0	
GP640_Pit1	HP45_Pit1	0	0	
GP640_Pit1	HP48_Pit1	0	0.001	
GP640_Pit1	HP91-2_Pit1	0	0.002	
GP640_Pit1	HP98_Pit1	0	0.001	
GP640_Pit1	HP103_Pit1	0.0036	0.0026	0.722222
GP640_Pit1	HP390_Pit1	0	0.0026	
GP640_Pit1	IL9_Pit1	0	0.001	
GP640_Pit1	Koshihikar_Pit1	0	0.001	
GP640_Pit1	KY131_Pit1	0	0.001	
GP640_Pit1	LG31_Pit1	0	0.001	
GP640_Pit1	UR28_Pit1	0	0.002	
GP640_Pit1	WYG7_Pit1	0	0.0026	
GP640_Pit1	GLA4_Pit1	0.0036	0.0026	0.722222
GP640_Pit1	GP3_Pit1	0.0036	0.0026	0.722222
GP640_Pit1	GP22_Pit1	0	0.0026	
GP640_Pit1	GP51_Pit1	0.095	0.0347	0.365263
GP640_Pit1	GP772-1_Pit1	0	0.0026	
GP640_Pit1	HP119_Pit1	0	0.0026	

GP640_Pit1	HP263_Pit1	0	0.0026	
GP640_Pit1	HP274_Pit1	0	0.0026	
GP640_Pit1	HP327_Pit1	0	0.0026	
GP640_Pit1	HP362-2_Pit1	0	0.0026	
GP640_Pit1	HP383_Pit1	0.095	0.0347	0.365263
GP640_Pit1	HP396_Pit1	0.0036	0.0026	0.722222
GP640_Pit1	HP407_Pit1	0	0.0026	
GP640_Pit1	HP492_Pit1	0	0.0026	
GP640_Pit1	HP517-1_Pit1	0	0.0026	
GP640_Pit1	HP577_Pit1	0.0036	0.0026	0.722222
GP640_Pit1	W0128_Pit1	0.0036	0.002	0.555556
GP640_Pit1	GP104_Pit1	0.0929	0.0347	0.37352
GP640_Pit1	GP124_Pit1	0.0929	0.0347	0.37352
GP640_Pit1	GP540_Pit1	0.095	0.0347	0.365263
GP640_Pit1	Kasalath_Pit1	0.0929	0.0347	0.37352
GP640_Pit1	W3105-1_Pit1	0.0929	0.0347	0.37352
GP640_Pit1	GP295-1_Pit1	0	0	
GP640_Pit1	HP486_Pit1	0.0141	0.0114	0.808511
GP640_Pit1	GP62_Pit1	0.0929	0.0347	0.37352
GP761-1_Pit1	DHX2_Pit1	0	0.002	
GP761-1_Pit1	GP551_Pit1	0	0.002	
GP761-1_Pit1	GP567_Pit1	0	0	
GP761-1_Pit1	GP669_Pit1	0	0.002	
GP761-1_Pit1	GP677_Pit1	0	0.002	
GP761-1_Pit1	HP13-2_Pit1	0.0036	0.0005	0.138889
GP761-1_Pit1	HP14_Pit1	0	0.002	
GP761-1_Pit1	HP38_Pit1	0	0	
GP761-1_Pit1	HP44_Pit1	0	0.002	
GP761-1_Pit1	HP45_Pit1	0	0.002	
GP761-1_Pit1	HP48_Pit1	0	0.002	
GP761-1_Pit1	HP91-2_Pit1	0	0	
GP761-1_Pit1	HP98_Pit1	0	0.002	
GP761-1_Pit1	HP103_Pit1	0.0036	0.0005	0.138889
GP761-1_Pit1	HP390_Pit1	0	0.0005	
GP761-1_Pit1	IL9_Pit1	0	0.002	
GP761-1_Pit1	Koshihikar_Pit1	0	0.002	
GP761-1_Pit1	KY131_Pit1	0	0.002	
GP761-1_Pit1	LG31_Pit1	0	0.002	
GP761-1_Pit1	UR28_Pit1	0	0	
GP761-1_Pit1	WYG7_Pit1	0	0.0005	
GP761-1_Pit1	GLA4_Pit1	0.0036	0.0005	0.138889
GP761-1_Pit1	GP3_Pit1	0.0036	0.0005	0.138889
GP761-1_Pit1	GP22_Pit1	0	0.0005	
GP761-1_Pit1	GP51_Pit1	0.095	0.0336	0.353684
GP761-1_Pit1	GP772-1_Pit1	0	0.0005	
GP761-1_Pit1	HP119_Pit1	0	0.0005	
GP761-1_Pit1	HP263_Pit1	0	0.0005	
GP761-1_Pit1	HP274_Pit1	0	0.0005	
GP761-1_Pit1	HP327_Pit1	0	0.0005	
GP761-1_Pit1	HP362-2_Pit1	0	0.0005	
GP761-1_Pit1	HP383_Pit1	0.095	0.0336	0.353684
GP761-1_Pit1	HP396_Pit1	0.0036	0.0005	0.138889
GP761-1_Pit1	HP407_Pit1	0	0.0005	
GP761-1_Pit1	HP492_Pit1	0	0.0005	
GP761-1_Pit1	HP517-1_Pit1	0	0.0005	
GP761-1_Pit1	HP577_Pit1	0.0036	0.0005	0.138889

GP761-1_Pit1	W0128_Pit1	0.0036	0.001	0.277778
GP761-1_Pit1	GP104_Pit1	0.0929	0.0336	0.361679
GP761-1_Pit1	GP124_Pit1	0.0929	0.0336	0.361679
GP761-1_Pit1	GP540_Pit1	0.095	0.0336	0.353684
GP761-1_Pit1	Kasalath_Pit1	0.0929	0.0336	0.361679
GP761-1_Pit1	W3105-1_Pit1	0.0929	0.0336	0.361679
GP761-1_Pit1	GP295-1_Pit1	0	0.002	
GP761-1_Pit1	HP486_Pit1	0.0141	0.0094	0.666667
GP761-1_Pit1	GP62_Pit1	0.0929	0.0336	0.361679
DHX2_Pit1	GP551_Pit1	0	0	
DHX2_Pit1	GP567_Pit1	0	0.002	
DHX2_Pit1	GP669_Pit1	0	0.001	
DHX2_Pit1	GP677_Pit1	0	0.001	
DHX2_Pit1	HP13-2_Pit1	0.0036	0.0026	0.722222
DHX2_Pit1	HP14_Pit1	0	0	
DHX2_Pit1	HP38_Pit1	0	0.002	
DHX2_Pit1	HP44_Pit1	0	0.001	
DHX2_Pit1	HP45_Pit1	0	0.001	
DHX2_Pit1	HP48_Pit1	0	0	
DHX2_Pit1	HP91-2_Pit1	0	0.002	
DHX2_Pit1	HP98_Pit1	0	0	
DHX2_Pit1	HP103_Pit1	0.0036	0.0026	0.722222
DHX2_Pit1	HP390_Pit1	0	0.0026	
DHX2_Pit1	IL9_Pit1	0	0	
DHX2_Pit1	Koshihikar_Pit1	0	0	
DHX2_Pit1	KY131_Pit1	0	0	
DHX2_Pit1	LG31_Pit1	0	0	
DHX2_Pit1	UR28_Pit1	0	0.002	
DHX2_Pit1	WYG7_Pit1	0	0.0026	
DHX2_Pit1	GLA4_Pit1	0.0036	0.0026	0.722222
DHX2_Pit1	GP3_Pit1	0.0036	0.0026	0.722222
DHX2_Pit1	GP22_Pit1	0	0.0026	
DHX2_Pit1	GP51_Pit1	0.0953	0.0346	0.363064
DHX2_Pit1	GP772-1_Pit1	0	0.0026	
DHX2_Pit1	HP119_Pit1	0	0.0026	
DHX2_Pit1	HP263_Pit1	0	0.0026	
DHX2_Pit1	HP274_Pit1	0	0.0026	
DHX2_Pit1	HP327_Pit1	0	0.0026	
DHX2_Pit1	HP362-2_Pit1	0	0.0026	
DHX2_Pit1	HP383_Pit1	0.0953	0.0346	0.363064
DHX2_Pit1	HP396_Pit1	0.0036	0.0026	0.722222
DHX2_Pit1	HP407_Pit1	0	0.0026	
DHX2_Pit1	HP492_Pit1	0	0.0026	
DHX2_Pit1	HP517-1_Pit1	0	0.0026	
DHX2_Pit1	HP577_Pit1	0.0036	0.0026	0.722222
DHX2_Pit1	W0128_Pit1	0.0036	0.002	0.555556
DHX2_Pit1	GP104_Pit1	0.0933	0.0346	0.370847
DHX2_Pit1	GP124_Pit1	0.0933	0.0346	0.370847
DHX2_Pit1	GP540_Pit1	0.0953	0.0346	0.363064
DHX2_Pit1	Kasalath_Pit1	0.0933	0.0346	0.370847
DHX2_Pit1	W3105-1_Pit1	0.0933	0.0346	0.370847
DHX2_Pit1	GP295-1_Pit1	0	0.001	
DHX2_Pit1	HP486_Pit1	0.0141	0.0114	0.808511
DHX2_Pit1	GP62_Pit1	0.0933	0.0346	0.370847
GP551_Pit1	GP567_Pit1	0	0.002	
GP551_Pit1	GP669_Pit1	0	0.001	

GP551_Pit1	GP677_Pit1	0	0.001	
GP551_Pit1	HP13-2_Pit1	0.0036	0.0026	0.722222
GP551_Pit1	HP14_Pit1	0	0	
GP551_Pit1	HP38_Pit1	0	0.002	
GP551_Pit1	HP44_Pit1	0	0.001	
GP551_Pit1	HP45_Pit1	0	0.001	
GP551_Pit1	HP48_Pit1	0	0	
GP551_Pit1	HP91-2_Pit1	0	0.002	
GP551_Pit1	HP98_Pit1	0	0	
GP551_Pit1	HP103_Pit1	0.0036	0.0026	0.722222
GP551_Pit1	HP390_Pit1	0	0.0026	
GP551_Pit1	IL9_Pit1	0	0	
GP551_Pit1	Koshihikar_Pit1	0	0	
GP551_Pit1	KY131_Pit1	0	0	
GP551_Pit1	LG31_Pit1	0	0	
GP551_Pit1	UR28_Pit1	0	0.002	
GP551_Pit1	WYG7_Pit1	0	0.0026	
GP551_Pit1	GLA4_Pit1	0.0036	0.0026	0.722222
GP551_Pit1	GP3_Pit1	0.0036	0.0026	0.722222
GP551_Pit1	GP22_Pit1	0	0.0026	
GP551_Pit1	GP51_Pit1	0.0953	0.0346	0.363064
GP551_Pit1	GP772-1_Pit1	0	0.0026	
GP551_Pit1	HP119_Pit1	0	0.0026	
GP551_Pit1	HP263_Pit1	0	0.0026	
GP551_Pit1	HP274_Pit1	0	0.0026	
GP551_Pit1	HP327_Pit1	0	0.0026	
GP551_Pit1	HP362-2_Pit1	0	0.0026	
GP551_Pit1	HP383_Pit1	0.0953	0.0346	0.363064
GP551_Pit1	HP396_Pit1	0.0036	0.0026	0.722222
GP551_Pit1	HP407_Pit1	0	0.0026	
GP551_Pit1	HP492_Pit1	0	0.0026	
GP551_Pit1	HP517-1_Pit1	0	0.0026	
GP551_Pit1	HP577_Pit1	0.0036	0.0026	0.722222
GP551_Pit1	W0128_Pit1	0.0036	0.002	0.555556
GP551_Pit1	GP104_Pit1	0.0933	0.0346	0.370847
GP551_Pit1	GP124_Pit1	0.0933	0.0346	0.370847
GP551_Pit1	GP540_Pit1	0.0953	0.0346	0.363064
GP551_Pit1	Kasalath_Pit1	0.0933	0.0346	0.370847
GP551_Pit1	W3105-1_Pit1	0.0933	0.0346	0.370847
GP551_Pit1	GP295-1_Pit1	0	0.001	
GP551_Pit1	HP486_Pit1	0.0141	0.0114	0.808511
GP551_Pit1	GP62_Pit1	0.0933	0.0346	0.370847
GP567_Pit1	GP669_Pit1	0	0.002	
GP567_Pit1	GP677_Pit1	0	0.002	
GP567_Pit1	HP13-2_Pit1	0.0036	0.0005	0.138889
GP567_Pit1	HP14_Pit1	0	0.002	
GP567_Pit1	HP38_Pit1	0	0	
GP567_Pit1	HP44_Pit1	0	0.002	
GP567_Pit1	HP45_Pit1	0	0.002	
GP567_Pit1	HP48_Pit1	0	0.002	
GP567_Pit1	HP91-2_Pit1	0	0	
GP567_Pit1	HP98_Pit1	0	0.002	
GP567_Pit1	HP103_Pit1	0.0036	0.0005	0.138889
GP567_Pit1	HP390_Pit1	0	0.0005	
GP567_Pit1	IL9_Pit1	0	0.002	
GP567_Pit1	Koshihikar_Pit1	0	0.002	

GP567_Pit1	KY131_Pit1	0	0.002	
GP567_Pit1	LG31_Pit1	0	0.002	
GP567_Pit1	UR28_Pit1	0	0	
GP567_Pit1	WYG7_Pit1	0	0.0005	
GP567_Pit1	GLA4_Pit1	0.0036	0.0005	0.138889
GP567_Pit1	GP3_Pit1	0.0036	0.0005	0.138889
GP567_Pit1	GP22_Pit1	0	0.0005	
GP567_Pit1	GP51_Pit1	0.095	0.0336	0.353684
GP567_Pit1	GP772-1_Pit1	0	0.0005	
GP567_Pit1	HP119_Pit1	0	0.0005	
GP567_Pit1	HP263_Pit1	0	0.0005	
GP567_Pit1	HP274_Pit1	0	0.0005	
GP567_Pit1	HP327_Pit1	0	0.0005	
GP567_Pit1	HP362-2_Pit1	0	0.0005	
GP567_Pit1	HP383_Pit1	0.095	0.0336	0.353684
GP567_Pit1	HP396_Pit1	0.0036	0.0005	0.138889
GP567_Pit1	HP407_Pit1	0	0.0005	
GP567_Pit1	HP492_Pit1	0	0.0005	
GP567_Pit1	HP517-1_Pit1	0	0.0005	
GP567_Pit1	HP577_Pit1	0.0036	0.0005	0.138889
GP567_Pit1	W0128_Pit1	0.0036	0.001	0.277778
GP567_Pit1	GP104_Pit1	0.0929	0.0336	0.361679
GP567_Pit1	GP124_Pit1	0.0929	0.0336	0.361679
GP567_Pit1	GP540_Pit1	0.095	0.0336	0.353684
GP567_Pit1	Kasalath_Pit1	0.0929	0.0336	0.361679
GP567_Pit1	W3105-1_Pit1	0.0929	0.0336	0.361679
GP567_Pit1	GP295-1_Pit1	0	0.002	
GP567_Pit1	HP486_Pit1	0.0141	0.0094	0.666667
GP567_Pit1	GP62_Pit1	0.0929	0.0336	0.361679
GP669_Pit1	GP677_Pit1	0	0	
GP669_Pit1	HP13-2_Pit1	0.0036	0.0026	0.722222
GP669_Pit1	HP14_Pit1	0	0.001	
GP669_Pit1	HP38_Pit1	0	0.002	
GP669_Pit1	HP44_Pit1	0	0	
GP669_Pit1	HP45_Pit1	0	0	
GP669_Pit1	HP48_Pit1	0	0.001	
GP669_Pit1	HP91-2_Pit1	0	0.002	
GP669_Pit1	HP98_Pit1	0	0.001	
GP669_Pit1	HP103_Pit1	0.0036	0.0026	0.722222
GP669_Pit1	HP390_Pit1	0	0.0026	
GP669_Pit1	IL9_Pit1	0	0.001	
GP669_Pit1	Koshihikar_Pit1	0	0.001	
GP669_Pit1	KY131_Pit1	0	0.001	
GP669_Pit1	LG31_Pit1	0	0.001	
GP669_Pit1	UR28_Pit1	0	0.002	
GP669_Pit1	WYG7_Pit1	0	0.0026	
GP669_Pit1	GLA4_Pit1	0.0036	0.0026	0.722222
GP669_Pit1	GP3_Pit1	0.0036	0.0026	0.722222
GP669_Pit1	GP22_Pit1	0	0.0026	
GP669_Pit1	GP51_Pit1	0.095	0.0347	0.365263
GP669_Pit1	GP772-1_Pit1	0	0.0026	
GP669_Pit1	HP119_Pit1	0	0.0026	
GP669_Pit1	HP263_Pit1	0	0.0026	
GP669_Pit1	HP274_Pit1	0	0.0026	
GP669_Pit1	HP327_Pit1	0	0.0026	
GP669_Pit1	HP362-2_Pit1	0	0.0026	

GP669_Pit1	HP383_Pit1	0.095	0.0347	0.365263
GP669_Pit1	HP396_Pit1	0.0036	0.0026	0.722222
GP669_Pit1	HP407_Pit1	0	0.0026	
GP669_Pit1	HP492_Pit1	0	0.0026	
GP669_Pit1	HP517-1_Pit1	0	0.0026	
GP669_Pit1	HP577_Pit1	0.0036	0.0026	0.722222
GP669_Pit1	W0128_Pit1	0.0036	0.002	0.555556
GP669_Pit1	GP104_Pit1	0.0929	0.0347	0.37352
GP669_Pit1	GP124_Pit1	0.0929	0.0347	0.37352
GP669_Pit1	GP540_Pit1	0.095	0.0347	0.365263
GP669_Pit1	Kasalath_Pit1	0.0929	0.0347	0.37352
GP669_Pit1	W3105-1_Pit1	0.0929	0.0347	0.37352
GP669_Pit1	GP295-1_Pit1	0	0	
GP669_Pit1	HP486_Pit1	0.0141	0.0114	0.808511
GP669_Pit1	GP62_Pit1	0.0929	0.0347	0.37352
GP677_Pit1	HP13-2_Pit1	0.0036	0.0026	0.722222
GP677_Pit1	HP14_Pit1	0	0.001	
GP677_Pit1	HP38_Pit1	0	0.002	
GP677_Pit1	HP44_Pit1	0	0	
GP677_Pit1	HP45_Pit1	0	0	
GP677_Pit1	HP48_Pit1	0	0.001	
GP677_Pit1	HP91-2_Pit1	0	0.002	
GP677_Pit1	HP98_Pit1	0	0.001	
GP677_Pit1	HP103_Pit1	0.0036	0.0026	0.722222
GP677_Pit1	HP390_Pit1	0	0.0026	
GP677_Pit1	IL9_Pit1	0	0.001	
GP677_Pit1	Koshihikar_Pit1	0	0.001	
GP677_Pit1	KY131_Pit1	0	0.001	
GP677_Pit1	LG31_Pit1	0	0.001	
GP677_Pit1	UR28_Pit1	0	0.002	
GP677_Pit1	WYG7_Pit1	0	0.0026	
GP677_Pit1	GLA4_Pit1	0.0036	0.0026	0.722222
GP677_Pit1	GP3_Pit1	0.0036	0.0026	0.722222
GP677_Pit1	GP22_Pit1	0	0.0026	
GP677_Pit1	GP51_Pit1	0.095	0.0347	0.365263
GP677_Pit1	GP772-1_Pit1	0	0.0026	
GP677_Pit1	HP119_Pit1	0	0.0026	
GP677_Pit1	HP263_Pit1	0	0.0026	
GP677_Pit1	HP274_Pit1	0	0.0026	
GP677_Pit1	HP327_Pit1	0	0.0026	
GP677_Pit1	HP362-2_Pit1	0	0.0026	
GP677_Pit1	HP383_Pit1	0.095	0.0347	0.365263
GP677_Pit1	HP396_Pit1	0.0036	0.0026	0.722222
GP677_Pit1	HP407_Pit1	0	0.0026	
GP677_Pit1	HP492_Pit1	0	0.0026	
GP677_Pit1	HP517-1_Pit1	0	0.0026	
GP677_Pit1	HP577_Pit1	0.0036	0.0026	0.722222
GP677_Pit1	W0128_Pit1	0.0036	0.002	0.555556
GP677_Pit1	GP104_Pit1	0.0929	0.0347	0.37352
GP677_Pit1	GP124_Pit1	0.0929	0.0347	0.37352
GP677_Pit1	GP540_Pit1	0.095	0.0347	0.365263
GP677_Pit1	Kasalath_Pit1	0.0929	0.0347	0.37352
GP677_Pit1	W3105-1_Pit1	0.0929	0.0347	0.37352
GP677_Pit1	GP295-1_Pit1	0	0	
GP677_Pit1	HP486_Pit1	0.0141	0.0114	0.808511
GP677_Pit1	GP62_Pit1	0.0929	0.0347	0.37352

HP13-2_Pit1	HP14_Pit1	0.0036	0.0026	0.722222
HP13-2_Pit1	HP38_Pit1	0.0036	0.0005	0.138889
HP13-2_Pit1	HP44_Pit1	0.0036	0.0026	0.722222
HP13-2_Pit1	HP45_Pit1	0.0036	0.0026	0.722222
HP13-2_Pit1	HP48_Pit1	0.0036	0.0026	0.722222
HP13-2_Pit1	HP91-2_Pit1	0.0036	0.0005	0.138889
HP13-2_Pit1	HP98_Pit1	0.0036	0.0026	0.722222
HP13-2_Pit1	HP103_Pit1	0	0	
HP13-2_Pit1	HP390_Pit1	0.0036	0.001	0.277778
HP13-2_Pit1	IL9_Pit1	0.0036	0.0026	0.722222
HP13-2_Pit1	Koshihikar_Pit1	0.0036	0.0026	0.722222
HP13-2_Pit1	KY131_Pit1	0.0036	0.0026	0.722222
HP13-2_Pit1	LG31_Pit1	0.0036	0.0026	0.722222
HP13-2_Pit1	UR28_Pit1	0.0036	0.0005	0.138889
HP13-2_Pit1	WYG7_Pit1	0.0036	0.001	0.277778
HP13-2_Pit1	GLA4_Pit1	0	0	
HP13-2_Pit1	GP3_Pit1	0	0	
HP13-2_Pit1	GP22_Pit1	0.0036	0.001	0.277778
HP13-2_Pit1	GP51_Pit1	0.0991	0.0342	0.345106
HP13-2_Pit1	GP772-1_Pit1	0.0036	0.001	0.277778
HP13-2_Pit1	HP119_Pit1	0.0036	0.001	0.277778
HP13-2_Pit1	HP263_Pit1	0.0036	0.001	0.277778
HP13-2_Pit1	HP274_Pit1	0.0036	0.001	0.277778
HP13-2_Pit1	HP327_Pit1	0.0036	0.001	0.277778
HP13-2_Pit1	HP362-2_Pit1	0.0036	0.001	0.277778
HP13-2_Pit1	HP383_Pit1	0.0991	0.0342	0.345106
HP13-2_Pit1	HP396_Pit1	0	0	
HP13-2_Pit1	HP407_Pit1	0.0036	0.001	0.277778
HP13-2_Pit1	HP492_Pit1	0.0036	0.001	0.277778
HP13-2_Pit1	HP517-1_Pit1	0.0036	0.001	0.277778
HP13-2_Pit1	HP577_Pit1	0	0	
HP13-2_Pit1	W0128_Pit1	0.0073	0.0015	0.205479
HP13-2_Pit1	GP104_Pit1	0.097	0.0342	0.352577
HP13-2_Pit1	GP124_Pit1	0.097	0.0342	0.352577
HP13-2_Pit1	GP540_Pit1	0.0991	0.0342	0.345106
HP13-2_Pit1	Kasalath_Pit1	0.097	0.0342	0.352577
HP13-2_Pit1	W3105-1_Pit1	0.097	0.0342	0.352577
HP13-2_Pit1	GP295-1_Pit1	0.0036	0.0026	0.722222
HP13-2_Pit1	HP486_Pit1	0.0104	0.0088	0.846154
HP13-2_Pit1	GP62_Pit1	0.097	0.0342	0.352577
HP14_Pit1	HP38_Pit1	0	0.002	
HP14_Pit1	HP44_Pit1	0	0.001	
HP14_Pit1	HP45_Pit1	0	0.001	
HP14_Pit1	HP48_Pit1	0	0	
HP14_Pit1	HP91-2_Pit1	0	0.002	
HP14_Pit1	HP98_Pit1	0	0	
HP14_Pit1	HP103_Pit1	0.0036	0.0026	0.722222
HP14_Pit1	HP390_Pit1	0	0.0026	
HP14_Pit1	IL9_Pit1	0	0	
HP14_Pit1	Koshihikar_Pit1	0	0	
HP14_Pit1	KY131_Pit1	0	0	
HP14_Pit1	LG31_Pit1	0	0	
HP14_Pit1	UR28_Pit1	0	0.002	
HP14_Pit1	WYG7_Pit1	0	0.0026	
HP14_Pit1	GLA4_Pit1	0.0036	0.0026	0.722222
HP14_Pit1	GP3_Pit1	0.0036	0.0026	0.722222

HP14_Pit1	GP22_Pit1	0	0.0026	
HP14_Pit1	GP51_Pit1	0.0953	0.0346	0.363064
HP14_Pit1	GP772-1_Pit1	0	0.0026	
HP14_Pit1	HP119_Pit1	0	0.0026	
HP14_Pit1	HP263_Pit1	0	0.0026	
HP14_Pit1	HP274_Pit1	0	0.0026	
HP14_Pit1	HP327_Pit1	0	0.0026	
HP14_Pit1	HP362-2_Pit1	0	0.0026	
HP14_Pit1	HP383_Pit1	0.0953	0.0346	0.363064
HP14_Pit1	HP396_Pit1	0.0036	0.0026	0.722222
HP14_Pit1	HP407_Pit1	0	0.0026	
HP14_Pit1	HP492_Pit1	0	0.0026	
HP14_Pit1	HP517-1_Pit1	0	0.0026	
HP14_Pit1	HP577_Pit1	0.0036	0.0026	0.722222
HP14_Pit1	W0128_Pit1	0.0036	0.002	0.555556
HP14_Pit1	GP104_Pit1	0.0933	0.0346	0.370847
HP14_Pit1	GP124_Pit1	0.0933	0.0346	0.370847
HP14_Pit1	GP540_Pit1	0.0953	0.0346	0.363064
HP14_Pit1	Kasalath_Pit1	0.0933	0.0346	0.370847
HP14_Pit1	W3105-1_Pit1	0.0933	0.0346	0.370847
HP14_Pit1	GP295-1_Pit1	0	0.001	
HP14_Pit1	HP486_Pit1	0.0141	0.0114	0.808511
HP14_Pit1	GP62_Pit1	0.0933	0.0346	0.370847
HP38_Pit1	HP44_Pit1	0	0.002	
HP38_Pit1	HP45_Pit1	0	0.002	
HP38_Pit1	HP48_Pit1	0	0.002	
HP38_Pit1	HP91-2_Pit1	0	0	
HP38_Pit1	HP98_Pit1	0	0.002	
HP38_Pit1	HP103_Pit1	0.0036	0.0005	0.138889
HP38_Pit1	HP390_Pit1	0	0.0005	
HP38_Pit1	IL9_Pit1	0	0.002	
HP38_Pit1	Koshihikar_Pit1	0	0.002	
HP38_Pit1	KY131_Pit1	0	0.002	
HP38_Pit1	LG31_Pit1	0	0.002	
HP38_Pit1	UR28_Pit1	0	0	
HP38_Pit1	WYG7_Pit1	0	0.0005	
HP38_Pit1	GLA4_Pit1	0.0036	0.0005	0.138889
HP38_Pit1	GP3_Pit1	0.0036	0.0005	0.138889
HP38_Pit1	GP22_Pit1	0	0.0005	
HP38_Pit1	GP51_Pit1	0.095	0.0336	0.353684
HP38_Pit1	GP772-1_Pit1	0	0.0005	
HP38_Pit1	HP119_Pit1	0	0.0005	
HP38_Pit1	HP263_Pit1	0	0.0005	
HP38_Pit1	HP274_Pit1	0	0.0005	
HP38_Pit1	HP327_Pit1	0	0.0005	
HP38_Pit1	HP362-2_Pit1	0	0.0005	
HP38_Pit1	HP383_Pit1	0.095	0.0336	0.353684
HP38_Pit1	HP396_Pit1	0.0036	0.0005	0.138889
HP38_Pit1	HP407_Pit1	0	0.0005	
HP38_Pit1	HP492_Pit1	0	0.0005	
HP38_Pit1	HP517-1_Pit1	0	0.0005	
HP38_Pit1	HP577_Pit1	0.0036	0.0005	0.138889
HP38_Pit1	W0128_Pit1	0.0036	0.001	0.277778
HP38_Pit1	GP104_Pit1	0.0929	0.0336	0.361679
HP38_Pit1	GP124_Pit1	0.0929	0.0336	0.361679
HP38_Pit1	GP540_Pit1	0.095	0.0336	0.353684

HP38_Pit1	Kasalath_Pit1	0.0929	0.0336	0.361679
HP38_Pit1	W3105-1_Pit1	0.0929	0.0336	0.361679
HP38_Pit1	GP295-1_Pit1	0	0.002	
HP38_Pit1	HP486_Pit1	0.0141	0.0094	0.666667
HP38_Pit1	GP62_Pit1	0.0929	0.0336	0.361679
HP44_Pit1	HP45_Pit1	0	0	
HP44_Pit1	HP48_Pit1	0	0.001	
HP44_Pit1	HP91-2_Pit1	0	0.002	
HP44_Pit1	HP98_Pit1	0	0.001	
HP44_Pit1	HP103_Pit1	0.0036	0.0026	0.722222
HP44_Pit1	HP390_Pit1	0	0.0026	
HP44_Pit1	IL9_Pit1	0	0.001	
HP44_Pit1	Koshihikar_Pit1	0	0.001	
HP44_Pit1	KY131_Pit1	0	0.001	
HP44_Pit1	LG31_Pit1	0	0.001	
HP44_Pit1	UR28_Pit1	0	0.002	
HP44_Pit1	WYG7_Pit1	0	0.0026	
HP44_Pit1	GLA4_Pit1	0.0036	0.0026	0.722222
HP44_Pit1	GP3_Pit1	0.0036	0.0026	0.722222
HP44_Pit1	GP22_Pit1	0	0.0026	
HP44_Pit1	GP51_Pit1	0.095	0.0347	0.365263
HP44_Pit1	GP772-1_Pit1	0	0.0026	
HP44_Pit1	HP119_Pit1	0	0.0026	
HP44_Pit1	HP263_Pit1	0	0.0026	
HP44_Pit1	HP274_Pit1	0	0.0026	
HP44_Pit1	HP327_Pit1	0	0.0026	
HP44_Pit1	HP362-2_Pit1	0	0.0026	
HP44_Pit1	HP383_Pit1	0.095	0.0347	0.365263
HP44_Pit1	HP396_Pit1	0.0036	0.0026	0.722222
HP44_Pit1	HP407_Pit1	0	0.0026	
HP44_Pit1	HP492_Pit1	0	0.0026	
HP44_Pit1	HP517-1_Pit1	0	0.0026	
HP44_Pit1	HP577_Pit1	0.0036	0.0026	0.722222
HP44_Pit1	W0128_Pit1	0.0036	0.002	0.555556
HP44_Pit1	GP104_Pit1	0.0929	0.0347	0.37352
HP44_Pit1	GP124_Pit1	0.0929	0.0347	0.37352
HP44_Pit1	GP540_Pit1	0.095	0.0347	0.365263
HP44_Pit1	Kasalath_Pit1	0.0929	0.0347	0.37352
HP44_Pit1	W3105-1_Pit1	0.0929	0.0347	0.37352
HP44_Pit1	GP295-1_Pit1	0	0	
HP44_Pit1	HP486_Pit1	0.0141	0.0114	0.808511
HP44_Pit1	GP62_Pit1	0.0929	0.0347	0.37352
HP45_Pit1	HP48_Pit1	0	0.001	
HP45_Pit1	HP91-2_Pit1	0	0.002	
HP45_Pit1	HP98_Pit1	0	0.001	
HP45_Pit1	HP103_Pit1	0.0036	0.0026	0.722222
HP45_Pit1	HP390_Pit1	0	0.0026	
HP45_Pit1	IL9_Pit1	0	0.001	
HP45_Pit1	Koshihikar_Pit1	0	0.001	
HP45_Pit1	KY131_Pit1	0	0.001	
HP45_Pit1	LG31_Pit1	0	0.001	
HP45_Pit1	UR28_Pit1	0	0.002	
HP45_Pit1	WYG7_Pit1	0	0.0026	
HP45_Pit1	GLA4_Pit1	0.0036	0.0026	0.722222
HP45_Pit1	GP3_Pit1	0.0036	0.0026	0.722222
HP45_Pit1	GP22_Pit1	0	0.0026	

HP45_Pit1	GP51_Pit1	0.095	0.0347	0.365263
HP45_Pit1	GP772-1_Pit1	0	0.0026	
HP45_Pit1	HP119_Pit1	0	0.0026	
HP45_Pit1	HP263_Pit1	0	0.0026	
HP45_Pit1	HP274_Pit1	0	0.0026	
HP45_Pit1	HP327_Pit1	0	0.0026	
HP45_Pit1	HP362-2_Pit1	0	0.0026	
HP45_Pit1	HP383_Pit1	0.095	0.0347	0.365263
HP45_Pit1	HP396_Pit1	0.0036	0.0026	0.722222
HP45_Pit1	HP407_Pit1	0	0.0026	
HP45_Pit1	HP492_Pit1	0	0.0026	
HP45_Pit1	HP517-1_Pit1	0	0.0026	
HP45_Pit1	HP577_Pit1	0.0036	0.0026	0.722222
HP45_Pit1	W0128_Pit1	0.0036	0.002	0.555556
HP45_Pit1	GP104_Pit1	0.0929	0.0347	0.37352
HP45_Pit1	GP124_Pit1	0.0929	0.0347	0.37352
HP45_Pit1	GP540_Pit1	0.095	0.0347	0.365263
HP45_Pit1	Kasalath_Pit1	0.0929	0.0347	0.37352
HP45_Pit1	W3105-1_Pit1	0.0929	0.0347	0.37352
HP45_Pit1	GP295-1_Pit1	0	0	
HP45_Pit1	HP486_Pit1	0.0141	0.0114	0.808511
HP45_Pit1	GP62_Pit1	0.0929	0.0347	0.37352
HP48_Pit1	HP91-2_Pit1	0	0.002	
HP48_Pit1	HP98_Pit1	0	0	
HP48_Pit1	HP103_Pit1	0.0036	0.0026	0.722222
HP48_Pit1	HP390_Pit1	0	0.0026	
HP48_Pit1	IL9_Pit1	0	0	
HP48_Pit1	Koshihikar_Pit1	0	0	
HP48_Pit1	KY131_Pit1	0	0	
HP48_Pit1	LG31_Pit1	0	0	
HP48_Pit1	UR28_Pit1	0	0.002	
HP48_Pit1	WYG7_Pit1	0	0.0026	
HP48_Pit1	GLA4_Pit1	0.0036	0.0026	0.722222
HP48_Pit1	GP3_Pit1	0.0036	0.0026	0.722222
HP48_Pit1	GP22_Pit1	0	0.0026	
HP48_Pit1	GP51_Pit1	0.0953	0.0346	0.363064
HP48_Pit1	GP772-1_Pit1	0	0.0026	
HP48_Pit1	HP119_Pit1	0	0.0026	
HP48_Pit1	HP263_Pit1	0	0.0026	
HP48_Pit1	HP274_Pit1	0	0.0026	
HP48_Pit1	HP327_Pit1	0	0.0026	
HP48_Pit1	HP362-2_Pit1	0	0.0026	
HP48_Pit1	HP383_Pit1	0.0953	0.0346	0.363064
HP48_Pit1	HP396_Pit1	0.0036	0.0026	0.722222
HP48_Pit1	HP407_Pit1	0	0.0026	
HP48_Pit1	HP492_Pit1	0	0.0026	
HP48_Pit1	HP517-1_Pit1	0	0.0026	
HP48_Pit1	HP577_Pit1	0.0036	0.0026	0.722222
HP48_Pit1	W0128_Pit1	0.0036	0.002	0.555556
HP48_Pit1	GP104_Pit1	0.0933	0.0346	0.370847
HP48_Pit1	GP124_Pit1	0.0933	0.0346	0.370847
HP48_Pit1	GP540_Pit1	0.0953	0.0346	0.363064
HP48_Pit1	Kasalath_Pit1	0.0933	0.0346	0.370847
HP48_Pit1	W3105-1_Pit1	0.0933	0.0346	0.370847
HP48_Pit1	GP295-1_Pit1	0	0.001	
HP48_Pit1	HP486_Pit1	0.0141	0.0114	0.808511

HP48_Pit1	GP62_Pit1	0.0933	0.0346	0.370847
HP91-2_Pit1	HP98_Pit1	0	0.002	
HP91-2_Pit1	HP103_Pit1	0.0036	0.0005	0.138889
HP91-2_Pit1	HP390_Pit1	0	0.0005	
HP91-2_Pit1	IL9_Pit1	0	0.002	
HP91-2_Pit1	Koshihikar_Pit1	0	0.002	
HP91-2_Pit1	KY131_Pit1	0	0.002	
HP91-2_Pit1	LG31_Pit1	0	0.002	
HP91-2_Pit1	UR28_Pit1	0	0	
HP91-2_Pit1	WYG7_Pit1	0	0.0005	
HP91-2_Pit1	GLA4_Pit1	0.0036	0.0005	0.138889
HP91-2_Pit1	GP3_Pit1	0.0036	0.0005	0.138889
HP91-2_Pit1	GP22_Pit1	0	0.0005	
HP91-2_Pit1	GP51_Pit1	0.095	0.0336	0.353684
HP91-2_Pit1	GP772-1_Pit1	0	0.0005	
HP91-2_Pit1	HP119_Pit1	0	0.0005	
HP91-2_Pit1	HP263_Pit1	0	0.0005	
HP91-2_Pit1	HP274_Pit1	0	0.0005	
HP91-2_Pit1	HP327_Pit1	0	0.0005	
HP91-2_Pit1	HP362-2_Pit1	0	0.0005	
HP91-2_Pit1	HP383_Pit1	0.095	0.0336	0.353684
HP91-2_Pit1	HP396_Pit1	0.0036	0.0005	0.138889
HP91-2_Pit1	HP407_Pit1	0	0.0005	
HP91-2_Pit1	HP492_Pit1	0	0.0005	
HP91-2_Pit1	HP517-1_Pit1	0	0.0005	
HP91-2_Pit1	HP577_Pit1	0.0036	0.0005	0.138889
HP91-2_Pit1	W0128_Pit1	0.0036	0.001	0.277778
HP91-2_Pit1	GP104_Pit1	0.0929	0.0336	0.361679
HP91-2_Pit1	GP124_Pit1	0.0929	0.0336	0.361679
HP91-2_Pit1	GP540_Pit1	0.095	0.0336	0.353684
HP91-2_Pit1	Kasalath_Pit1	0.0929	0.0336	0.361679
HP91-2_Pit1	W3105-1_Pit1	0.0929	0.0336	0.361679
HP91-2_Pit1	GP295-1_Pit1	0	0.002	
HP91-2_Pit1	HP486_Pit1	0.0141	0.0094	0.666667
HP91-2_Pit1	GP62_Pit1	0.0929	0.0336	0.361679
HP98_Pit1	HP103_Pit1	0.0036	0.0026	0.722222
HP98_Pit1	HP390_Pit1	0	0.0026	
HP98_Pit1	IL9_Pit1	0	0	
HP98_Pit1	Koshihikar_Pit1	0	0	
HP98_Pit1	KY131_Pit1	0	0	
HP98_Pit1	LG31_Pit1	0	0	
HP98_Pit1	UR28_Pit1	0	0.002	
HP98_Pit1	WYG7_Pit1	0	0.0026	
HP98_Pit1	GLA4_Pit1	0.0036	0.0026	0.722222
HP98_Pit1	GP3_Pit1	0.0036	0.0026	0.722222
HP98_Pit1	GP22_Pit1	0	0.0026	
HP98_Pit1	GP51_Pit1	0.0953	0.0346	0.363064
HP98_Pit1	GP772-1_Pit1	0	0.0026	
HP98_Pit1	HP119_Pit1	0	0.0026	
HP98_Pit1	HP263_Pit1	0	0.0026	
HP98_Pit1	HP274_Pit1	0	0.0026	
HP98_Pit1	HP327_Pit1	0	0.0026	
HP98_Pit1	HP362-2_Pit1	0	0.0026	
HP98_Pit1	HP383_Pit1	0.0953	0.0346	0.363064
HP98_Pit1	HP396_Pit1	0.0036	0.0026	0.722222
HP98_Pit1	HP407_Pit1	0	0.0026	

HP98_Pit1	HP492_Pit1	0	0.0026	
HP98_Pit1	HP517-1_Pit1	0	0.0026	
HP98_Pit1	HP577_Pit1	0.0036	0.0026	0.722222
HP98_Pit1	W0128_Pit1	0.0036	0.002	0.555556
HP98_Pit1	GP104_Pit1	0.0933	0.0346	0.370847
HP98_Pit1	GP124_Pit1	0.0933	0.0346	0.370847
HP98_Pit1	GP540_Pit1	0.0953	0.0346	0.363064
HP98_Pit1	Kasalath_Pit1	0.0933	0.0346	0.370847
HP98_Pit1	W3105-1_Pit1	0.0933	0.0346	0.370847
HP98_Pit1	GP295-1_Pit1	0	0.001	
HP98_Pit1	HP486_Pit1	0.0141	0.0114	0.808511
HP98_Pit1	GP62_Pit1	0.0933	0.0346	0.370847
HP103_Pit1	HP390_Pit1	0.0036	0.001	0.277778
HP103_Pit1	IL9_Pit1	0.0036	0.0026	0.722222
HP103_Pit1	Koshihikar_Pit1	0.0036	0.0026	0.722222
HP103_Pit1	KY131_Pit1	0.0036	0.0026	0.722222
HP103_Pit1	LG31_Pit1	0.0036	0.0026	0.722222
HP103_Pit1	UR28_Pit1	0.0036	0.0005	0.138889
HP103_Pit1	WYG7_Pit1	0.0036	0.001	0.277778
HP103_Pit1	GLA4_Pit1	0	0	
HP103_Pit1	GP3_Pit1	0	0	
HP103_Pit1	GP22_Pit1	0.0036	0.001	0.277778
HP103_Pit1	GP51_Pit1	0.0991	0.0342	0.345106
HP103_Pit1	GP772-1_Pit1	0.0036	0.001	0.277778
HP103_Pit1	HP119_Pit1	0.0036	0.001	0.277778
HP103_Pit1	HP263_Pit1	0.0036	0.001	0.277778
HP103_Pit1	HP274_Pit1	0.0036	0.001	0.277778
HP103_Pit1	HP327_Pit1	0.0036	0.001	0.277778
HP103_Pit1	HP362-2_Pit1	0.0036	0.001	0.277778
HP103_Pit1	HP383_Pit1	0.0991	0.0342	0.345106
HP103_Pit1	HP396_Pit1	0	0	
HP103_Pit1	HP407_Pit1	0.0036	0.001	0.277778
HP103_Pit1	HP492_Pit1	0.0036	0.001	0.277778
HP103_Pit1	HP517-1_Pit1	0.0036	0.001	0.277778
HP103_Pit1	HP577_Pit1	0	0	
HP103_Pit1	W0128_Pit1	0.0073	0.0015	0.205479
HP103_Pit1	GP104_Pit1	0.097	0.0342	0.352577
HP103_Pit1	GP124_Pit1	0.097	0.0342	0.352577
HP103_Pit1	GP540_Pit1	0.0991	0.0342	0.345106
HP103_Pit1	Kasalath_Pit1	0.097	0.0342	0.352577
HP103_Pit1	W3105-1_Pit1	0.097	0.0342	0.352577
HP103_Pit1	GP295-1_Pit1	0.0036	0.0026	0.722222
HP103_Pit1	HP486_Pit1	0.0104	0.0088	0.846154
HP103_Pit1	GP62_Pit1	0.097	0.0342	0.352577
HP390_Pit1	IL9_Pit1	0	0.0026	
HP390_Pit1	Koshihikar_Pit1	0	0.0026	
HP390_Pit1	KY131_Pit1	0	0.0026	
HP390_Pit1	LG31_Pit1	0	0.0026	
HP390_Pit1	UR28_Pit1	0	0.0005	
HP390_Pit1	WYG7_Pit1	0	0	
HP390_Pit1	GLA4_Pit1	0.0036	0.001	0.277778
HP390_Pit1	GP3_Pit1	0.0036	0.001	0.277778
HP390_Pit1	GP22_Pit1	0	0	
HP390_Pit1	GP51_Pit1	0.095	0.0341	0.358947
HP390_Pit1	GP772-1_Pit1	0	0	
HP390_Pit1	HP119_Pit1	0	0	

HP390_Pit1	HP263_Pit1	0	0	
HP390_Pit1	HP274_Pit1	0	0	
HP390_Pit1	HP327_Pit1	0	0	
HP390_Pit1	HP362-2_Pit1	0	0	
HP390_Pit1	HP383_Pit1	0.095	0.0341	0.358947
HP390_Pit1	HP396_Pit1	0.0036	0.001	0.277778
HP390_Pit1	HP407_Pit1	0	0	
HP390_Pit1	HP492_Pit1	0	0	
HP390_Pit1	HP517-1_Pit1	0	0	
HP390_Pit1	HP577_Pit1	0.0036	0.001	0.277778
HP390_Pit1	W0128_Pit1	0.0036	0.0015	0.416667
HP390_Pit1	GP104_Pit1	0.093	0.0341	0.366667
HP390_Pit1	GP124_Pit1	0.093	0.0341	0.366667
HP390_Pit1	GP540_Pit1	0.095	0.0341	0.358947
HP390_Pit1	Kasalath_Pit1	0.093	0.0341	0.366667
HP390_Pit1	W3105-1_Pit1	0.093	0.0341	0.366667
HP390_Pit1	GP295-1_Pit1	0	0.0026	
HP390_Pit1	HP486_Pit1	0.0141	0.0099	0.702128
HP390_Pit1	GP62_Pit1	0.093	0.0341	0.366667
IL9_Pit1	Koshihikar_Pit1	0	0	
IL9_Pit1	KY131_Pit1	0	0	
IL9_Pit1	LG31_Pit1	0	0	
IL9_Pit1	UR28_Pit1	0	0.002	
IL9_Pit1	WYG7_Pit1	0	0.0026	
IL9_Pit1	GLA4_Pit1	0.0036	0.0026	0.722222
IL9_Pit1	GP3_Pit1	0.0036	0.0026	0.722222
IL9_Pit1	GP22_Pit1	0	0.0026	
IL9_Pit1	GP51_Pit1	0.0953	0.0346	0.363064
IL9_Pit1	GP772-1_Pit1	0	0.0026	
IL9_Pit1	HP119_Pit1	0	0.0026	
IL9_Pit1	HP263_Pit1	0	0.0026	
IL9_Pit1	HP274_Pit1	0	0.0026	
IL9_Pit1	HP327_Pit1	0	0.0026	
IL9_Pit1	HP362-2_Pit1	0	0.0026	
IL9_Pit1	HP383_Pit1	0.0953	0.0346	0.363064
IL9_Pit1	HP396_Pit1	0.0036	0.0026	0.722222
IL9_Pit1	HP407_Pit1	0	0.0026	
IL9_Pit1	HP492_Pit1	0	0.0026	
IL9_Pit1	HP517-1_Pit1	0	0.0026	
IL9_Pit1	HP577_Pit1	0.0036	0.0026	0.722222
IL9_Pit1	W0128_Pit1	0.0036	0.002	0.555556
IL9_Pit1	GP104_Pit1	0.0933	0.0346	0.370847
IL9_Pit1	GP124_Pit1	0.0933	0.0346	0.370847
IL9_Pit1	GP540_Pit1	0.0953	0.0346	0.363064
IL9_Pit1	Kasalath_Pit1	0.0933	0.0346	0.370847
IL9_Pit1	W3105-1_Pit1	0.0933	0.0346	0.370847
IL9_Pit1	GP295-1_Pit1	0	0.001	
IL9_Pit1	HP486_Pit1	0.0141	0.0114	0.808511
IL9_Pit1	GP62_Pit1	0.0933	0.0346	0.370847
Koshihikar_Pit1	KY131_Pit1	0	0	
Koshihikar_Pit1	LG31_Pit1	0	0	
Koshihikar_Pit1	UR28_Pit1	0	0.002	
Koshihikar_Pit1	WYG7_Pit1	0	0.0026	
Koshihikar_Pit1	GLA4_Pit1	0.0036	0.0026	0.722222
Koshihikar_Pit1	GP3_Pit1	0.0036	0.0026	0.722222
Koshihikar_Pit1	GP22_Pit1	0	0.0026	

Koshihikar_Pit1	GP51_Pit1	0.0953	0.0346	0.363064
Koshihikar_Pit1	GP772-1_Pit1	0	0.0026	
Koshihikar_Pit1	HP119_Pit1	0	0.0026	
Koshihikar_Pit1	HP263_Pit1	0	0.0026	
Koshihikar_Pit1	HP274_Pit1	0	0.0026	
Koshihikar_Pit1	HP327_Pit1	0	0.0026	
Koshihikar_Pit1	HP362-2_Pit1	0	0.0026	
Koshihikar_Pit1	HP383_Pit1	0.0953	0.0346	0.363064
Koshihikar_Pit1	HP396_Pit1	0.0036	0.0026	0.722222
Koshihikar_Pit1	HP407_Pit1	0	0.0026	
Koshihikar_Pit1	HP492_Pit1	0	0.0026	
Koshihikar_Pit1	HP517-1_Pit1	0	0.0026	
Koshihikar_Pit1	HP577_Pit1	0.0036	0.0026	0.722222
Koshihikar_Pit1	W0128_Pit1	0.0036	0.002	0.555556
Koshihikar_Pit1	GP104_Pit1	0.0933	0.0346	0.370847
Koshihikar_Pit1	GP124_Pit1	0.0933	0.0346	0.370847
Koshihikar_Pit1	GP540_Pit1	0.0953	0.0346	0.363064
Koshihikar_Pit1	Kasalath_Pit1	0.0933	0.0346	0.370847
Koshihikar_Pit1	W3105-1_Pit1	0.0933	0.0346	0.370847
Koshihikar_Pit1	GP295-1_Pit1	0	0.001	
Koshihikar_Pit1	HP486_Pit1	0.0141	0.0114	0.808511
Koshihikar_Pit1	GP62_Pit1	0.0933	0.0346	0.370847
KY131_Pit1	LG31_Pit1	0	0	
KY131_Pit1	UR28_Pit1	0	0.002	
KY131_Pit1	WYG7_Pit1	0	0.0026	
KY131_Pit1	GLA4_Pit1	0.0036	0.0026	0.722222
KY131_Pit1	GP3_Pit1	0.0036	0.0026	0.722222
KY131_Pit1	GP22_Pit1	0	0.0026	
KY131_Pit1	GP51_Pit1	0.0953	0.0346	0.363064
KY131_Pit1	GP772-1_Pit1	0	0.0026	
KY131_Pit1	HP119_Pit1	0	0.0026	
KY131_Pit1	HP263_Pit1	0	0.0026	
KY131_Pit1	HP274_Pit1	0	0.0026	
KY131_Pit1	HP327_Pit1	0	0.0026	
KY131_Pit1	HP362-2_Pit1	0	0.0026	
KY131_Pit1	HP383_Pit1	0.0953	0.0346	0.363064
KY131_Pit1	HP396_Pit1	0.0036	0.0026	0.722222
KY131_Pit1	HP407_Pit1	0	0.0026	
KY131_Pit1	HP492_Pit1	0	0.0026	
KY131_Pit1	HP517-1_Pit1	0	0.0026	
KY131_Pit1	HP577_Pit1	0.0036	0.0026	0.722222
KY131_Pit1	W0128_Pit1	0.0036	0.002	0.555556
KY131_Pit1	GP104_Pit1	0.0933	0.0346	0.370847
KY131_Pit1	GP124_Pit1	0.0933	0.0346	0.370847
KY131_Pit1	GP540_Pit1	0.0953	0.0346	0.363064
KY131_Pit1	Kasalath_Pit1	0.0933	0.0346	0.370847
KY131_Pit1	W3105-1_Pit1	0.0933	0.0346	0.370847
KY131_Pit1	GP295-1_Pit1	0	0.001	
KY131_Pit1	HP486_Pit1	0.0141	0.0114	0.808511
KY131_Pit1	GP62_Pit1	0.0933	0.0346	0.370847
LG31_Pit1	UR28_Pit1	0	0.002	
LG31_Pit1	WYG7_Pit1	0	0.0026	
LG31_Pit1	GLA4_Pit1	0.0036	0.0026	0.722222
LG31_Pit1	GP3_Pit1	0.0036	0.0026	0.722222
LG31_Pit1	GP22_Pit1	0	0.0026	
LG31_Pit1	GP51_Pit1	0.0953	0.0346	0.363064

LG31_Pit1	GP772-1_Pit1	0	0.0026	
LG31_Pit1	HP119_Pit1	0	0.0026	
LG31_Pit1	HP263_Pit1	0	0.0026	
LG31_Pit1	HP274_Pit1	0	0.0026	
LG31_Pit1	HP327_Pit1	0	0.0026	
LG31_Pit1	HP362-2_Pit1	0	0.0026	
LG31_Pit1	HP383_Pit1	0.0953	0.0346	0.363064
LG31_Pit1	HP396_Pit1	0.0036	0.0026	0.722222
LG31_Pit1	HP407_Pit1	0	0.0026	
LG31_Pit1	HP492_Pit1	0	0.0026	
LG31_Pit1	HP517-1_Pit1	0	0.0026	
LG31_Pit1	HP577_Pit1	0.0036	0.0026	0.722222
LG31_Pit1	W0128_Pit1	0.0036	0.002	0.555556
LG31_Pit1	GP104_Pit1	0.0933	0.0346	0.370847
LG31_Pit1	GP124_Pit1	0.0933	0.0346	0.370847
LG31_Pit1	GP540_Pit1	0.0953	0.0346	0.363064
LG31_Pit1	Kasalath_Pit1	0.0933	0.0346	0.370847
LG31_Pit1	W3105-1_Pit1	0.0933	0.0346	0.370847
LG31_Pit1	GP295-1_Pit1	0	0.001	
LG31_Pit1	HP486_Pit1	0.0141	0.0114	0.808511
LG31_Pit1	GP62_Pit1	0.0933	0.0346	0.370847
UR28_Pit1	WYG7_Pit1	0	0.0005	
UR28_Pit1	GLA4_Pit1	0.0036	0.0005	0.138889
UR28_Pit1	GP3_Pit1	0.0036	0.0005	0.138889
UR28_Pit1	GP22_Pit1	0	0.0005	
UR28_Pit1	GP51_Pit1	0.095	0.0336	0.353684
UR28_Pit1	GP772-1_Pit1	0	0.0005	
UR28_Pit1	HP119_Pit1	0	0.0005	
UR28_Pit1	HP263_Pit1	0	0.0005	
UR28_Pit1	HP274_Pit1	0	0.0005	
UR28_Pit1	HP327_Pit1	0	0.0005	
UR28_Pit1	HP362-2_Pit1	0	0.0005	
UR28_Pit1	HP383_Pit1	0.095	0.0336	0.353684
UR28_Pit1	HP396_Pit1	0.0036	0.0005	0.138889
UR28_Pit1	HP407_Pit1	0	0.0005	
UR28_Pit1	HP492_Pit1	0	0.0005	
UR28_Pit1	HP517-1_Pit1	0	0.0005	
UR28_Pit1	HP577_Pit1	0.0036	0.0005	0.138889
UR28_Pit1	W0128_Pit1	0.0036	0.001	0.277778
UR28_Pit1	GP104_Pit1	0.0929	0.0336	0.361679
UR28_Pit1	GP124_Pit1	0.0929	0.0336	0.361679
UR28_Pit1	GP540_Pit1	0.095	0.0336	0.353684
UR28_Pit1	Kasalath_Pit1	0.0929	0.0336	0.361679
UR28_Pit1	W3105-1_Pit1	0.0929	0.0336	0.361679
UR28_Pit1	GP295-1_Pit1	0	0.002	
UR28_Pit1	HP486_Pit1	0.0141	0.0094	0.666667
UR28_Pit1	GP62_Pit1	0.0929	0.0336	0.361679
WYG7_Pit1	GLA4_Pit1	0.0036	0.001	0.277778
WYG7_Pit1	GP3_Pit1	0.0036	0.001	0.277778
WYG7_Pit1	GP22_Pit1	0	0	
WYG7_Pit1	GP51_Pit1	0.095	0.0341	0.358947
WYG7_Pit1	GP772-1_Pit1	0	0	
WYG7_Pit1	HP119_Pit1	0	0	
WYG7_Pit1	HP263_Pit1	0	0	
WYG7_Pit1	HP274_Pit1	0	0	
WYG7_Pit1	HP327_Pit1	0	0	

WYG7_Pit1	HP362-2_Pit1	0	0	
WYG7_Pit1	HP383_Pit1	0.095	0.0341	0.358947
WYG7_Pit1	HP396_Pit1	0.0036	0.001	0.277778
WYG7_Pit1	HP407_Pit1	0	0	
WYG7_Pit1	HP492_Pit1	0	0	
WYG7_Pit1	HP517-1_Pit1	0	0	
WYG7_Pit1	HP577_Pit1	0.0036	0.001	0.277778
WYG7_Pit1	W0128_Pit1	0.0036	0.0015	0.416667
WYG7_Pit1	GP104_Pit1	0.093	0.0341	0.366667
WYG7_Pit1	GP124_Pit1	0.093	0.0341	0.366667
WYG7_Pit1	GP540_Pit1	0.095	0.0341	0.358947
WYG7_Pit1	Kasalath_Pit1	0.093	0.0341	0.366667
WYG7_Pit1	W3105-1_Pit1	0.093	0.0341	0.366667
WYG7_Pit1	GP295-1_Pit1	0	0.0026	
WYG7_Pit1	HP486_Pit1	0.0141	0.0099	0.702128
WYG7_Pit1	GP62_Pit1	0.093	0.0341	0.366667
GLA4_Pit1	GP3_Pit1	0	0	
GLA4_Pit1	GP22_Pit1	0.0036	0.001	0.277778
GLA4_Pit1	GP51_Pit1	0.0991	0.0342	0.345106
GLA4_Pit1	GP772-1_Pit1	0.0036	0.001	0.277778
GLA4_Pit1	HP119_Pit1	0.0036	0.001	0.277778
GLA4_Pit1	HP263_Pit1	0.0036	0.001	0.277778
GLA4_Pit1	HP274_Pit1	0.0036	0.001	0.277778
GLA4_Pit1	HP327_Pit1	0.0036	0.001	0.277778
GLA4_Pit1	HP362-2_Pit1	0.0036	0.001	0.277778
GLA4_Pit1	HP383_Pit1	0.0991	0.0342	0.345106
GLA4_Pit1	HP396_Pit1	0	0	
GLA4_Pit1	HP407_Pit1	0.0036	0.001	0.277778
GLA4_Pit1	HP492_Pit1	0.0036	0.001	0.277778
GLA4_Pit1	HP517-1_Pit1	0.0036	0.001	0.277778
GLA4_Pit1	HP577_Pit1	0	0	
GLA4_Pit1	W0128_Pit1	0.0073	0.0015	0.205479
GLA4_Pit1	GP104_Pit1	0.097	0.0342	0.352577
GLA4_Pit1	GP124_Pit1	0.097	0.0342	0.352577
GLA4_Pit1	GP540_Pit1	0.0991	0.0342	0.345106
GLA4_Pit1	Kasalath_Pit1	0.097	0.0342	0.352577
GLA4_Pit1	W3105-1_Pit1	0.097	0.0342	0.352577
GLA4_Pit1	GP295-1_Pit1	0.0036	0.0026	0.722222
GLA4_Pit1	HP486_Pit1	0.0104	0.0088	0.846154
GLA4_Pit1	GP62_Pit1	0.097	0.0342	0.352577
GP3_Pit1	GP22_Pit1	0.0036	0.001	0.277778
GP3_Pit1	GP51_Pit1	0.0991	0.0342	0.345106
GP3_Pit1	GP772-1_Pit1	0.0036	0.001	0.277778
GP3_Pit1	HP119_Pit1	0.0036	0.001	0.277778
GP3_Pit1	HP263_Pit1	0.0036	0.001	0.277778
GP3_Pit1	HP274_Pit1	0.0036	0.001	0.277778
GP3_Pit1	HP327_Pit1	0.0036	0.001	0.277778
GP3_Pit1	HP362-2_Pit1	0.0036	0.001	0.277778
GP3_Pit1	HP383_Pit1	0.0991	0.0342	0.345106
GP3_Pit1	HP396_Pit1	0	0	
GP3_Pit1	HP407_Pit1	0.0036	0.001	0.277778
GP3_Pit1	HP492_Pit1	0.0036	0.001	0.277778
GP3_Pit1	HP517-1_Pit1	0.0036	0.001	0.277778
GP3_Pit1	HP577_Pit1	0	0	
GP3_Pit1	W0128_Pit1	0.0073	0.0015	0.205479
GP3_Pit1	GP104_Pit1	0.097	0.0342	0.352577

GP3_Pit1	GP124_Pit1	0.097	0.0342	0.352577
GP3_Pit1	GP540_Pit1	0.0991	0.0342	0.345106
GP3_Pit1	Kasalath_Pit1	0.097	0.0342	0.352577
GP3_Pit1	W3105-1_Pit1	0.097	0.0342	0.352577
GP3_Pit1	GP295-1_Pit1	0.0036	0.0026	0.722222
GP3_Pit1	HP486_Pit1	0.0104	0.0088	0.846154
GP3_Pit1	GP62_Pit1	0.097	0.0342	0.352577
GP22_Pit1	GP51_Pit1	0.095	0.0341	0.358947
GP22_Pit1	GP772-1_Pit1	0	0	
GP22_Pit1	HP119_Pit1	0	0	
GP22_Pit1	HP263_Pit1	0	0	
GP22_Pit1	HP274_Pit1	0	0	
GP22_Pit1	HP327_Pit1	0	0	
GP22_Pit1	HP362-2_Pit1	0	0	
GP22_Pit1	HP383_Pit1	0.095	0.0341	0.358947
GP22_Pit1	HP396_Pit1	0.0036	0.001	0.277778
GP22_Pit1	HP407_Pit1	0	0	
GP22_Pit1	HP492_Pit1	0	0	
GP22_Pit1	HP517-1_Pit1	0	0	
GP22_Pit1	HP577_Pit1	0.0036	0.001	0.277778
GP22_Pit1	W0128_Pit1	0.0036	0.0015	0.416667
GP22_Pit1	GP104_Pit1	0.093	0.0341	0.366667
GP22_Pit1	GP124_Pit1	0.093	0.0341	0.366667
GP22_Pit1	GP540_Pit1	0.095	0.0341	0.358947
GP22_Pit1	Kasalath_Pit1	0.093	0.0341	0.366667
GP22_Pit1	W3105-1_Pit1	0.093	0.0341	0.366667
GP22_Pit1	GP295-1_Pit1	0	0.0026	
GP22_Pit1	HP486_Pit1	0.0141	0.0099	0.702128
GP22_Pit1	GP62_Pit1	0.093	0.0341	0.366667
GP51_Pit1	GP772-1_Pit1	0.095	0.0341	0.358947
GP51_Pit1	HP119_Pit1	0.095	0.0341	0.358947
GP51_Pit1	HP263_Pit1	0.095	0.0341	0.358947
GP51_Pit1	HP274_Pit1	0.095	0.0341	0.358947
GP51_Pit1	HP327_Pit1	0.095	0.0341	0.358947
GP51_Pit1	HP362-2_Pit1	0.095	0.0341	0.358947
GP51_Pit1	HP383_Pit1	0	0	
GP51_Pit1	HP396_Pit1	0.0991	0.0342	0.345106
GP51_Pit1	HP407_Pit1	0.095	0.0341	0.358947
GP51_Pit1	HP492_Pit1	0.095	0.0341	0.358947
GP51_Pit1	HP517-1_Pit1	0.095	0.0341	0.358947
GP51_Pit1	HP577_Pit1	0.0991	0.0342	0.345106
GP51_Pit1	W0128_Pit1	0.0992	0.0336	0.33871
GP51_Pit1	GP104_Pit1	0.0018	0.001	0.555556
GP51_Pit1	GP124_Pit1	0.0018	0.001	0.555556
GP51_Pit1	GP540_Pit1	0	0	
GP51_Pit1	Kasalath_Pit1	0.0018	0.001	0.555556
GP51_Pit1	W3105-1_Pit1	0.0018	0.001	0.555556
GP51_Pit1	GP295-1_Pit1	0.095	0.0347	0.365263
GP51_Pit1	HP486_Pit1	0.1059	0.0425	0.401322
GP51_Pit1	GP62_Pit1	0.0018	0.001	0.555556
GP772-1_Pit1	HP119_Pit1	0	0	
GP772-1_Pit1	HP263_Pit1	0	0	
GP772-1_Pit1	HP274_Pit1	0	0	
GP772-1_Pit1	HP327_Pit1	0	0	
GP772-1_Pit1	HP362-2_Pit1	0	0	
GP772-1_Pit1	HP383_Pit1	0.095	0.0341	0.358947

GP772-1_Pit1	HP396_Pit1	0.0036	0.001	0.277778
GP772-1_Pit1	HP407_Pit1	0	0	
GP772-1_Pit1	HP492_Pit1	0	0	
GP772-1_Pit1	HP517-1_Pit1	0	0	
GP772-1_Pit1	HP577_Pit1	0.0036	0.001	0.277778
GP772-1_Pit1	W0128_Pit1	0.0036	0.0015	0.416667
GP772-1_Pit1	GP104_Pit1	0.093	0.0341	0.366667
GP772-1_Pit1	GP124_Pit1	0.093	0.0341	0.366667
GP772-1_Pit1	GP540_Pit1	0.095	0.0341	0.358947
GP772-1_Pit1	Kasalath_Pit1	0.093	0.0341	0.366667
GP772-1_Pit1	W3105-1_Pit1	0.093	0.0341	0.366667
GP772-1_Pit1	GP295-1_Pit1	0	0.0026	
GP772-1_Pit1	HP486_Pit1	0.0141	0.0099	0.702128
GP772-1_Pit1	GP62_Pit1	0.093	0.0341	0.366667
HP119_Pit1	HP263_Pit1	0	0	
HP119_Pit1	HP274_Pit1	0	0	
HP119_Pit1	HP327_Pit1	0	0	
HP119_Pit1	HP362-2_Pit1	0	0	
HP119_Pit1	HP383_Pit1	0.095	0.0341	0.358947
HP119_Pit1	HP396_Pit1	0.0036	0.001	0.277778
HP119_Pit1	HP407_Pit1	0	0	
HP119_Pit1	HP492_Pit1	0	0	
HP119_Pit1	HP517-1_Pit1	0	0	
HP119_Pit1	HP577_Pit1	0.0036	0.001	0.277778
HP119_Pit1	W0128_Pit1	0.0036	0.0015	0.416667
HP119_Pit1	GP104_Pit1	0.093	0.0341	0.366667
HP119_Pit1	GP124_Pit1	0.093	0.0341	0.366667
HP119_Pit1	GP540_Pit1	0.095	0.0341	0.358947
HP119_Pit1	Kasalath_Pit1	0.093	0.0341	0.366667
HP119_Pit1	W3105-1_Pit1	0.093	0.0341	0.366667
HP119_Pit1	GP295-1_Pit1	0	0.0026	
HP119_Pit1	HP486_Pit1	0.0141	0.0099	0.702128
HP119_Pit1	GP62_Pit1	0.093	0.0341	0.366667
HP263_Pit1	HP274_Pit1	0	0	
HP263_Pit1	HP327_Pit1	0	0	
HP263_Pit1	HP362-2_Pit1	0	0	
HP263_Pit1	HP383_Pit1	0.095	0.0341	0.358947
HP263_Pit1	HP396_Pit1	0.0036	0.001	0.277778
HP263_Pit1	HP407_Pit1	0	0	
HP263_Pit1	HP492_Pit1	0	0	
HP263_Pit1	HP517-1_Pit1	0	0	
HP263_Pit1	HP577_Pit1	0.0036	0.001	0.277778
HP263_Pit1	W0128_Pit1	0.0036	0.0015	0.416667
HP263_Pit1	GP104_Pit1	0.093	0.0341	0.366667
HP263_Pit1	GP124_Pit1	0.093	0.0341	0.366667
HP263_Pit1	GP540_Pit1	0.095	0.0341	0.358947
HP263_Pit1	Kasalath_Pit1	0.093	0.0341	0.366667
HP263_Pit1	W3105-1_Pit1	0.093	0.0341	0.366667
HP263_Pit1	GP295-1_Pit1	0	0.0026	
HP263_Pit1	HP486_Pit1	0.0141	0.0099	0.702128
HP263_Pit1	GP62_Pit1	0.093	0.0341	0.366667
HP274_Pit1	HP327_Pit1	0	0	
HP274_Pit1	HP362-2_Pit1	0	0	
HP274_Pit1	HP383_Pit1	0.095	0.0341	0.358947
HP274_Pit1	HP396_Pit1	0.0036	0.001	0.277778
HP274_Pit1	HP407_Pit1	0	0	

HP274_Pit1	HP492_Pit1	0	0	
HP274_Pit1	HP517-1_Pit1	0	0	
HP274_Pit1	HP577_Pit1	0.0036	0.001	0.277778
HP274_Pit1	W0128_Pit1	0.0036	0.0015	0.416667
HP274_Pit1	GP104_Pit1	0.093	0.0341	0.366667
HP274_Pit1	GP124_Pit1	0.093	0.0341	0.366667
HP274_Pit1	GP540_Pit1	0.095	0.0341	0.358947
HP274_Pit1	Kasalath_Pit1	0.093	0.0341	0.366667
HP274_Pit1	W3105-1_Pit1	0.093	0.0341	0.366667
HP274_Pit1	GP295-1_Pit1	0	0.0026	
HP274_Pit1	HP486_Pit1	0.0141	0.0099	0.702128
HP274_Pit1	GP62_Pit1	0.093	0.0341	0.366667
HP327_Pit1	HP362-2_Pit1	0	0	
HP327_Pit1	HP383_Pit1	0.095	0.0341	0.358947
HP327_Pit1	HP396_Pit1	0.0036	0.001	0.277778
HP327_Pit1	HP407_Pit1	0	0	
HP327_Pit1	HP492_Pit1	0	0	
HP327_Pit1	HP517-1_Pit1	0	0	
HP327_Pit1	HP577_Pit1	0.0036	0.001	0.277778
HP327_Pit1	W0128_Pit1	0.0036	0.0015	0.416667
HP327_Pit1	GP104_Pit1	0.093	0.0341	0.366667
HP327_Pit1	GP124_Pit1	0.093	0.0341	0.366667
HP327_Pit1	GP540_Pit1	0.095	0.0341	0.358947
HP327_Pit1	Kasalath_Pit1	0.093	0.0341	0.366667
HP327_Pit1	W3105-1_Pit1	0.093	0.0341	0.366667
HP327_Pit1	GP295-1_Pit1	0	0.0026	
HP327_Pit1	HP486_Pit1	0.0141	0.0099	0.702128
HP327_Pit1	GP62_Pit1	0.093	0.0341	0.366667
HP362-2_Pit1	HP383_Pit1	0.095	0.0341	0.358947
HP362-2_Pit1	HP396_Pit1	0.0036	0.001	0.277778
HP362-2_Pit1	HP407_Pit1	0	0	
HP362-2_Pit1	HP492_Pit1	0	0	
HP362-2_Pit1	HP517-1_Pit1	0	0	
HP362-2_Pit1	HP577_Pit1	0.0036	0.001	0.277778
HP362-2_Pit1	W0128_Pit1	0.0036	0.0015	0.416667
HP362-2_Pit1	GP104_Pit1	0.093	0.0341	0.366667
HP362-2_Pit1	GP124_Pit1	0.093	0.0341	0.366667
HP362-2_Pit1	GP540_Pit1	0.095	0.0341	0.358947
HP362-2_Pit1	Kasalath_Pit1	0.093	0.0341	0.366667
HP362-2_Pit1	W3105-1_Pit1	0.093	0.0341	0.366667
HP362-2_Pit1	GP295-1_Pit1	0	0.0026	
HP362-2_Pit1	HP486_Pit1	0.0141	0.0099	0.702128
HP362-2_Pit1	GP62_Pit1	0.093	0.0341	0.366667
HP383_Pit1	HP396_Pit1	0.0991	0.0342	0.345106
HP383_Pit1	HP407_Pit1	0.095	0.0341	0.358947
HP383_Pit1	HP492_Pit1	0.095	0.0341	0.358947
HP383_Pit1	HP517-1_Pit1	0.095	0.0341	0.358947
HP383_Pit1	HP577_Pit1	0.0991	0.0342	0.345106
HP383_Pit1	W0128_Pit1	0.0992	0.0336	0.33871
HP383_Pit1	GP104_Pit1	0.0018	0.001	0.555556
HP383_Pit1	GP124_Pit1	0.0018	0.001	0.555556
HP383_Pit1	GP540_Pit1	0	0	
HP383_Pit1	Kasalath_Pit1	0.0018	0.001	0.555556
HP383_Pit1	W3105-1_Pit1	0.0018	0.001	0.555556
HP383_Pit1	GP295-1_Pit1	0.095	0.0347	0.365263
HP383_Pit1	HP486_Pit1	0.1059	0.0425	0.401322

HP383_Pit1	GP62_Pit1	0.0018	0.001	0.555556
HP396_Pit1	HP407_Pit1	0.0036	0.001	0.277778
HP396_Pit1	HP492_Pit1	0.0036	0.001	0.277778
HP396_Pit1	HP517-1_Pit1	0.0036	0.001	0.277778
HP396_Pit1	HP577_Pit1	0	0	
HP396_Pit1	W0128_Pit1	0.0073	0.0015	0.205479
HP396_Pit1	GP104_Pit1	0.097	0.0342	0.352577
HP396_Pit1	GP124_Pit1	0.097	0.0342	0.352577
HP396_Pit1	GP540_Pit1	0.0991	0.0342	0.345106
HP396_Pit1	Kasalath_Pit1	0.097	0.0342	0.352577
HP396_Pit1	W3105-1_Pit1	0.097	0.0342	0.352577
HP396_Pit1	GP295-1_Pit1	0.0036	0.0026	0.722222
HP396_Pit1	HP486_Pit1	0.0104	0.0088	0.846154
HP396_Pit1	GP62_Pit1	0.097	0.0342	0.352577
HP407_Pit1	HP492_Pit1	0	0	
HP407_Pit1	HP517-1_Pit1	0	0	
HP407_Pit1	HP577_Pit1	0.0036	0.001	0.277778
HP407_Pit1	W0128_Pit1	0.0036	0.0015	0.416667
HP407_Pit1	GP104_Pit1	0.093	0.0341	0.366667
HP407_Pit1	GP124_Pit1	0.093	0.0341	0.366667
HP407_Pit1	GP540_Pit1	0.095	0.0341	0.358947
HP407_Pit1	Kasalath_Pit1	0.093	0.0341	0.366667
HP407_Pit1	W3105-1_Pit1	0.093	0.0341	0.366667
HP407_Pit1	GP295-1_Pit1	0	0.0026	
HP407_Pit1	HP486_Pit1	0.0141	0.0099	0.702128
HP407_Pit1	GP62_Pit1	0.093	0.0341	0.366667
HP492_Pit1	HP517-1_Pit1	0	0	
HP492_Pit1	HP577_Pit1	0.0036	0.001	0.277778
HP492_Pit1	W0128_Pit1	0.0036	0.0015	0.416667
HP492_Pit1	GP104_Pit1	0.093	0.0341	0.366667
HP492_Pit1	GP124_Pit1	0.093	0.0341	0.366667
HP492_Pit1	GP540_Pit1	0.095	0.0341	0.358947
HP492_Pit1	Kasalath_Pit1	0.093	0.0341	0.366667
HP492_Pit1	W3105-1_Pit1	0.093	0.0341	0.366667
HP492_Pit1	GP295-1_Pit1	0	0.0026	
HP492_Pit1	HP486_Pit1	0.0141	0.0099	0.702128
HP492_Pit1	GP62_Pit1	0.093	0.0341	0.366667
HP517-1_Pit1	HP577_Pit1	0.0036	0.001	0.277778
HP517-1_Pit1	W0128_Pit1	0.0036	0.0015	0.416667
HP517-1_Pit1	GP104_Pit1	0.093	0.0341	0.366667
HP517-1_Pit1	GP124_Pit1	0.093	0.0341	0.366667
HP517-1_Pit1	GP540_Pit1	0.095	0.0341	0.358947
HP517-1_Pit1	Kasalath_Pit1	0.093	0.0341	0.366667
HP517-1_Pit1	W3105-1_Pit1	0.093	0.0341	0.366667
HP517-1_Pit1	GP295-1_Pit1	0	0.0026	
HP517-1_Pit1	HP486_Pit1	0.0141	0.0099	0.702128
HP517-1_Pit1	GP62_Pit1	0.093	0.0341	0.366667
HP577_Pit1	W0128_Pit1	0.0073	0.0015	0.205479
HP577_Pit1	GP104_Pit1	0.097	0.0342	0.352577
HP577_Pit1	GP124_Pit1	0.097	0.0342	0.352577
HP577_Pit1	GP540_Pit1	0.0991	0.0342	0.345106
HP577_Pit1	Kasalath_Pit1	0.097	0.0342	0.352577
HP577_Pit1	W3105-1_Pit1	0.097	0.0342	0.352577
HP577_Pit1	GP295-1_Pit1	0.0036	0.0026	0.722222
HP577_Pit1	HP486_Pit1	0.0104	0.0088	0.846154
HP577_Pit1	GP62_Pit1	0.097	0.0342	0.352577

W0128_Pit1	GP104_Pit1	0.0972	0.0336	0.345679
W0128_Pit1	GP124_Pit1	0.0972	0.0336	0.345679
W0128_Pit1	GP540_Pit1	0.0992	0.0336	0.33871
W0128_Pit1	Kasalath_Pit1	0.0972	0.0336	0.345679
W0128_Pit1	W3105-1_Pit1	0.0972	0.0336	0.345679
W0128_Pit1	GP295-1_Pit1	0.0036	0.002	0.555556
W0128_Pit1	HP486_Pit1	0.0178	0.0104	0.58427
W0128_Pit1	GP62_Pit1	0.0972	0.0336	0.345679
GP104_Pit1	GP124_Pit1	0	0	
GP104_Pit1	GP540_Pit1	0.0018	0.001	0.555556
GP104_Pit1	Kasalath_Pit1	0	0	
GP104_Pit1	W3105-1_Pit1	0	0	
GP104_Pit1	GP295-1_Pit1	0.0929	0.0347	0.37352
GP104_Pit1	HP486_Pit1	0.1038	0.0425	0.409441
GP104_Pit1	GP62_Pit1	0	0	
GP124_Pit1	GP540_Pit1	0.0018	0.001	0.555556
GP124_Pit1	Kasalath_Pit1	0	0	
GP124_Pit1	W3105-1_Pit1	0	0	
GP124_Pit1	GP295-1_Pit1	0.0929	0.0347	0.37352
GP124_Pit1	HP486_Pit1	0.1038	0.0425	0.409441
GP124_Pit1	GP62_Pit1	0	0	
GP540_Pit1	Kasalath_Pit1	0.0018	0.001	0.555556
GP540_Pit1	W3105-1_Pit1	0.0018	0.001	0.555556
GP540_Pit1	GP295-1_Pit1	0.095	0.0347	0.365263
GP540_Pit1	HP486_Pit1	0.1059	0.0425	0.401322
GP540_Pit1	GP62_Pit1	0.0018	0.001	0.555556
Kasalath_Pit1	W3105-1_Pit1	0	0	
Kasalath_Pit1	GP295-1_Pit1	0.0929	0.0347	0.37352
Kasalath_Pit1	HP486_Pit1	0.1038	0.0425	0.409441
Kasalath_Pit1	GP62_Pit1	0	0	
W3105-1_Pit1	GP295-1_Pit1	0.0929	0.0347	0.37352
W3105-1_Pit1	HP486_Pit1	0.1038	0.0425	0.409441
W3105-1_Pit1	GP62_Pit1	0	0	
GP295-1_Pit1	HP486_Pit1	0.0141	0.0114	0.808511
GP295-1_Pit1	GP62_Pit1	0.0929	0.0347	0.37352
HP486_Pit1	GP62_Pit1	0.1038	0.0425	0.409441

Supplementary Table 6: The Ka/Ks ratios for Pit2 genes in O.sativa.

Gene 1	Gene 2	Ks	Ka	Ka/Ks
K59_Pit2	Nipponbare_Pit2	0.0064	0.0012	0.1875
K59_Pit2	GP39_Pit2	0.0572	0.0417	0.729021
K59_Pit2	GP77_Pit2	0.0572	0.0417	0.729021
K59_Pit2	GP536_Pit2	0.0572	0.0417	0.729021
K59_Pit2	GP640_Pit2	0.0064	0.0012	0.1875
K59_Pit2	GP761-1_Pit2	0.0572	0.0417	0.729021
K59_Pit2	DHX2_Pit2	0.0064	0.0042	0.65625
K59_Pit2	GP551_Pit2	0.0064	0.0042	0.65625
K59_Pit2	GP567_Pit2	0.0572	0.0417	0.729021
K59_Pit2	GP669_Pit2	0.0064	0.0012	0.1875
K59_Pit2	GP677_Pit2	0.0064	0.0012	0.1875
K59_Pit2	HP13-2_Pit2	0.0572	0.0423	0.73951
K59_Pit2	HP14_Pit2	0.0064	0.0036	0.5625
K59_Pit2	HP38_Pit2	0.0043	0.0018	0.418605
K59_Pit2	HP44_Pit2	0.0064	0.0012	0.1875
K59_Pit2	HP45_Pit2	0.0064	0.0012	0.1875
K59_Pit2	HP48_Pit2	0.0064	0.0036	0.5625
K59_Pit2	HP91-2_Pit2	0.0572	0.0417	0.729021
K59_Pit2	HP98_Pit2	0.0064	0.0042	0.65625
K59_Pit2	HP103_Pit2	0.0572	0.0423	0.73951
K59_Pit2	HP390_Pit2	0.0594	0.0423	0.712121
K59_Pit2	IL9_Pit2	0.0064	0.0042	0.65625
K59_Pit2	Koshihikari_Pit2	0.0064	0.0042	0.65625
K59_Pit2	KY131_Pit2	0.0064	0.0042	0.65625
K59_Pit2	LG31_Pit2	0.0064	0.0042	0.65625
K59_Pit2	UR28_Pit2	0.0572	0.0417	0.729021
K59_Pit2	WYG7_Pit2	0.0594	0.0423	0.712121
K59_Pit2	GLA4_Pit2	0.0572	0.0423	0.73951
K59_Pit2	GP3_Pit2	0.0572	0.0423	0.73951
K59_Pit2	GP22_Pit2	0.063	0.042	0.666667
K59_Pit2	GP51_Pit2	0.0085	0.0006	0.070588
K59_Pit2	GP772-1_Pit2	0.0594	0.0423	0.712121
K59_Pit2	HP119_Pit2	0.0594	0.0423	0.712121
K59_Pit2	HP263_Pit2	0.0594	0.0423	0.712121
K59_Pit2	HP274_Pit2	0.063	0.042	0.666667
K59_Pit2	HP327_Pit2	0.0594	0.0423	0.712121
K59_Pit2	HP362-2_Pit2	0.0594	0.0423	0.712121
K59_Pit2	HP383_Pit2	0.0085	0.0006	0.070588
K59_Pit2	HP396_Pit2	0.0572	0.0423	0.73951
K59_Pit2	HP407_Pit2	0.0594	0.0423	0.712121
K59_Pit2	HP492_Pit2	0.0594	0.0423	0.712121
K59_Pit2	HP517-1_Pit2	0.0594	0.0423	0.712121
K59_Pit2	HP577_Pit2	0.0572	0.0423	0.73951
K59_Pit2	W0128_Pit2	0	0	
K59_Pit2	GP104_Pit2	0.0021	0.0006	0.285714
K59_Pit2	GP124_Pit2	0.0021	0.0006	0.285714
K59_Pit2	GP540_Pit2	0.0085	0.0006	0.070588
K59_Pit2	Kasalath_Pit2	0.0021	0.0006	0.285714
K59_Pit2	W3105-1_Pit2	0.0021	0.0006	0.285714
K59_Pit2	GP295-1_Pit2	0.0064	0.0012	0.1875
K59_Pit2	HP486_Pit2	0.0572	0.0423	0.73951
K59_Pit2	GP62_Pit2	0.015	0.0097	0.646667
Nipponbare_P	GP39_Pit2	0.055	0.043	0.781818
Nipponbare_P	GP77_Pit2	0.055	0.043	0.781818

Nipponbare_P	GP536_Pit2	0.055	0.043	0.781818
Nipponbare_P	GP640_Pit2	0	0	
Nipponbare_P	GP761-1_Pit2	0.055	0.043	0.781818
Nipponbare_P	DHX2_Pit2	0.0043	0.0042	0.976744
Nipponbare_P	GP551_Pit2	0.0043	0.0042	0.976744
Nipponbare_P	GP567_Pit2	0.055	0.043	0.781818
Nipponbare_P	GP669_Pit2	0	0	
Nipponbare_P	GP677_Pit2	0	0	
Nipponbare_P	HP13-2_Pit2	0.0549	0.0436	0.794171
Nipponbare_P	HP14_Pit2	0.0043	0.0036	0.837209
Nipponbare_P	HP38_Pit2	0.0021	0.003	1.428571
Nipponbare_P	HP44_Pit2	0	0	
Nipponbare_P	HP45_Pit2	0	0	
Nipponbare_P	HP48_Pit2	0.0043	0.0036	0.837209
Nipponbare_P	HP91-2_Pit2	0.055	0.043	0.781818
Nipponbare_P	HP98_Pit2	0.0043	0.0042	0.976744
Nipponbare_P	HP103_Pit2	0.0549	0.0436	0.794171
Nipponbare_P	HP390_Pit2	0.0572	0.0436	0.762238
Nipponbare_P	IL9_Pit2	0.0043	0.0042	0.976744
Nipponbare_P	Koshihikari_Pit2	0.0043	0.0042	0.976744
Nipponbare_P	KY131_Pit2	0.0043	0.0042	0.976744
Nipponbare_P	LG31_Pit2	0.0043	0.0042	0.976744
Nipponbare_P	UR28_Pit2	0.055	0.043	0.781818
Nipponbare_P	WYG7_Pit2	0.0572	0.0436	0.762238
Nipponbare_P	GLA4_Pit2	0.0549	0.0436	0.794171
Nipponbare_P	GP3_Pit2	0.0549	0.0436	0.794171
Nipponbare_P	GP22_Pit2	0.0607	0.0433	0.713344
Nipponbare_P	GP51_Pit2	0.0064	0.0006	0.09375
Nipponbare_P	GP772-1_Pit2	0.0572	0.0436	0.762238
Nipponbare_P	HP119_Pit2	0.0572	0.0436	0.762238
Nipponbare_P	HP263_Pit2	0.0572	0.0436	0.762238
Nipponbare_P	HP274_Pit2	0.0607	0.0433	0.713344
Nipponbare_P	HP327_Pit2	0.0572	0.0436	0.762238
Nipponbare_P	HP362-2_Pit2	0.0572	0.0436	0.762238
Nipponbare_P	HP383_Pit2	0.0064	0.0006	0.09375
Nipponbare_P	HP396_Pit2	0.0549	0.0436	0.794171
Nipponbare_P	HP407_Pit2	0.0572	0.0436	0.762238
Nipponbare_P	HP492_Pit2	0.0572	0.0436	0.762238
Nipponbare_P	HP517-1_Pit2	0.0572	0.0436	0.762238
Nipponbare_P	HP577_Pit2	0.0549	0.0436	0.794171
Nipponbare_P	W0128_Pit2	0.0064	0.0012	0.1875
Nipponbare_P	GP104_Pit2	0.0085	0.0018	0.211765
Nipponbare_P	GP124_Pit2	0.0085	0.0018	0.211765
Nipponbare_P	GP540_Pit2	0.0064	0.0006	0.09375
Nipponbare_P	Kasalath_Pit2	0.0085	0.0018	0.211765
Nipponbare_P	W3105-1_Pit2	0.0085	0.0018	0.211765
Nipponbare_P	GP295-1_Pit2	0	0	
Nipponbare_P	HP486_Pit2	0.0549	0.0436	0.794171
Nipponbare_P	GP62_Pit2	0.0215	0.011	0.511628
GP39_Pit2	GP77_Pit2	0	0	
GP39_Pit2	GP536_Pit2	0	0	
GP39_Pit2	GP640_Pit2	0.055	0.043	0.781818
GP39_Pit2	GP761-1_Pit2	0	0	
GP39_Pit2	DHX2_Pit2	0.0549	0.0449	0.817851
GP39_Pit2	GP551_Pit2	0.0549	0.0449	0.817851
GP39_Pit2	GP567_Pit2	0	0	

GP39_Pit2	GP669_Pit2	0.055	0.043	0.781818
GP39_Pit2	GP677_Pit2	0.055	0.043	0.781818
GP39_Pit2	HP13-2_Pit2	0	0.003	
GP39_Pit2	HP14_Pit2	0.055	0.0455	0.827273
GP39_Pit2	HP38_Pit2	0.0528	0.0397	0.751894
GP39_Pit2	HP44_Pit2	0.055	0.043	0.781818
GP39_Pit2	HP45_Pit2	0.055	0.043	0.781818
GP39_Pit2	HP48_Pit2	0.055	0.0455	0.827273
GP39_Pit2	HP91-2_Pit2	0	0	
GP39_Pit2	HP98_Pit2	0.0549	0.0449	0.817851
GP39_Pit2	HP103_Pit2	0	0.003	
GP39_Pit2	HP390_Pit2	0.0021	0.003	1.428571
GP39_Pit2	IL9_Pit2	0.0549	0.0449	0.817851
GP39_Pit2	Koshihikari_Pit2	0.0549	0.0449	0.817851
GP39_Pit2	KY131_Pit2	0.0549	0.0449	0.817851
GP39_Pit2	LG31_Pit2	0.0549	0.0449	0.817851
GP39_Pit2	UR28_Pit2	0	0	
GP39_Pit2	WYG7_Pit2	0.0021	0.003	1.428571
GP39_Pit2	GLA4_Pit2	0	0.003	
GP39_Pit2	GP3_Pit2	0	0.003	
GP39_Pit2	GP22_Pit2	0.0106	0.0104	0.981132
GP39_Pit2	GP51_Pit2	0.0572	0.0423	0.73951
GP39_Pit2	GP772-1_Pit2	0.0021	0.003	1.428571
GP39_Pit2	HP119_Pit2	0.0021	0.003	1.428571
GP39_Pit2	HP263_Pit2	0.0021	0.003	1.428571
GP39_Pit2	HP274_Pit2	0.0106	0.0104	0.981132
GP39_Pit2	HP327_Pit2	0.0021	0.003	1.428571
GP39_Pit2	HP362-2_Pit2	0.0021	0.003	1.428571
GP39_Pit2	HP383_Pit2	0.0572	0.0423	0.73951
GP39_Pit2	HP396_Pit2	0	0.003	
GP39_Pit2	HP407_Pit2	0.0021	0.003	1.428571
GP39_Pit2	HP492_Pit2	0.0021	0.003	1.428571
GP39_Pit2	HP517-1_Pit2	0.0021	0.003	1.428571
GP39_Pit2	HP577_Pit2	0	0.003	
GP39_Pit2	W0128_Pit2	0.0572	0.0417	0.729021
GP39_Pit2	GP104_Pit2	0.0595	0.0423	0.710924
GP39_Pit2	GP124_Pit2	0.0595	0.0423	0.710924
GP39_Pit2	GP540_Pit2	0.0572	0.0423	0.73951
GP39_Pit2	Kasalath_Pit2	0.0595	0.0423	0.710924
GP39_Pit2	W3105-1_Pit2	0.0595	0.0423	0.710924
GP39_Pit2	GP295-1_Pit2	0.055	0.043	0.781818
GP39_Pit2	HP486_Pit2	0	0.003	
GP39_Pit2	GP62_Pit2	0.0579	0.0466	0.804836
GP77_Pit2	GP536_Pit2	0	0	
GP77_Pit2	GP640_Pit2	0.055	0.043	0.781818
GP77_Pit2	GP761-1_Pit2	0	0	
GP77_Pit2	DHX2_Pit2	0.0549	0.0449	0.817851
GP77_Pit2	GP551_Pit2	0.0549	0.0449	0.817851
GP77_Pit2	GP567_Pit2	0	0	
GP77_Pit2	GP669_Pit2	0.055	0.043	0.781818
GP77_Pit2	GP677_Pit2	0.055	0.043	0.781818
GP77_Pit2	HP13-2_Pit2	0	0.003	
GP77_Pit2	HP14_Pit2	0.055	0.0455	0.827273
GP77_Pit2	HP38_Pit2	0.0528	0.0397	0.751894
GP77_Pit2	HP44_Pit2	0.055	0.043	0.781818
GP77_Pit2	HP45_Pit2	0.055	0.043	0.781818

GP77_Pit2	HP48_Pit2	0.055	0.0455	0.827273
GP77_Pit2	HP91-2_Pit2	0	0	
GP77_Pit2	HP98_Pit2	0.0549	0.0449	0.817851
GP77_Pit2	HP103_Pit2	0	0.003	
GP77_Pit2	HP390_Pit2	0.0021	0.003	1.428571
GP77_Pit2	IL9_Pit2	0.0549	0.0449	0.817851
GP77_Pit2	Koshihikari_Pit2	0.0549	0.0449	0.817851
GP77_Pit2	KY131_Pit2	0.0549	0.0449	0.817851
GP77_Pit2	LG31_Pit2	0.0549	0.0449	0.817851
GP77_Pit2	UR28_Pit2	0	0	
GP77_Pit2	WYG7_Pit2	0.0021	0.003	1.428571
GP77_Pit2	GLA4_Pit2	0	0.003	
GP77_Pit2	GP3_Pit2	0	0.003	
GP77_Pit2	GP22_Pit2	0.0106	0.0104	0.981132
GP77_Pit2	GP51_Pit2	0.0572	0.0423	0.73951
GP77_Pit2	GP772-1_Pit2	0.0021	0.003	1.428571
GP77_Pit2	HP119_Pit2	0.0021	0.003	1.428571
GP77_Pit2	HP263_Pit2	0.0021	0.003	1.428571
GP77_Pit2	HP274_Pit2	0.0106	0.0104	0.981132
GP77_Pit2	HP327_Pit2	0.0021	0.003	1.428571
GP77_Pit2	HP362-2_Pit2	0.0021	0.003	1.428571
GP77_Pit2	HP383_Pit2	0.0572	0.0423	0.73951
GP77_Pit2	HP396_Pit2	0	0.003	
GP77_Pit2	HP407_Pit2	0.0021	0.003	1.428571
GP77_Pit2	HP492_Pit2	0.0021	0.003	1.428571
GP77_Pit2	HP517-1_Pit2	0.0021	0.003	1.428571
GP77_Pit2	HP577_Pit2	0	0.003	
GP77_Pit2	W0128_Pit2	0.0572	0.0417	0.729021
GP77_Pit2	GP104_Pit2	0.0595	0.0423	0.710924
GP77_Pit2	GP124_Pit2	0.0595	0.0423	0.710924
GP77_Pit2	GP540_Pit2	0.0572	0.0423	0.73951
GP77_Pit2	Kasalath_Pit2	0.0595	0.0423	0.710924
GP77_Pit2	W3105-1_Pit2	0.0595	0.0423	0.710924
GP77_Pit2	GP295-1_Pit2	0.055	0.043	0.781818
GP77_Pit2	HP486_Pit2	0	0.003	
GP77_Pit2	GP62_Pit2	0.0579	0.0466	0.804836
GP536_Pit2	GP640_Pit2	0.055	0.043	0.781818
GP536_Pit2	GP761-1_Pit2	0	0	
GP536_Pit2	DHX2_Pit2	0.0549	0.0449	0.817851
GP536_Pit2	GP551_Pit2	0.0549	0.0449	0.817851
GP536_Pit2	GP567_Pit2	0	0	
GP536_Pit2	GP669_Pit2	0.055	0.043	0.781818
GP536_Pit2	GP677_Pit2	0.055	0.043	0.781818
GP536_Pit2	HP13-2_Pit2	0	0.003	
GP536_Pit2	HP14_Pit2	0.055	0.0455	0.827273
GP536_Pit2	HP38_Pit2	0.0528	0.0397	0.751894
GP536_Pit2	HP44_Pit2	0.055	0.043	0.781818
GP536_Pit2	HP45_Pit2	0.055	0.043	0.781818
GP536_Pit2	HP48_Pit2	0.055	0.0455	0.827273
GP536_Pit2	HP91-2_Pit2	0	0	
GP536_Pit2	HP98_Pit2	0.0549	0.0449	0.817851
GP536_Pit2	HP103_Pit2	0	0.003	
GP536_Pit2	HP390_Pit2	0.0021	0.003	1.428571
GP536_Pit2	IL9_Pit2	0.0549	0.0449	0.817851
GP536_Pit2	Koshihikari_Pit2	0.0549	0.0449	0.817851
GP536_Pit2	KY131_Pit2	0.0549	0.0449	0.817851

GP536_Pit2	LG31_Pit2	0.0549	0.0449	0.817851
GP536_Pit2	UR28_Pit2	0	0	
GP536_Pit2	WYG7_Pit2	0.0021	0.003	1.428571
GP536_Pit2	GLA4_Pit2	0	0.003	
GP536_Pit2	GP3_Pit2	0	0.003	
GP536_Pit2	GP22_Pit2	0.0106	0.0104	0.981132
GP536_Pit2	GP51_Pit2	0.0572	0.0423	0.73951
GP536_Pit2	GP772-1_Pit2	0.0021	0.003	1.428571
GP536_Pit2	HP119_Pit2	0.0021	0.003	1.428571
GP536_Pit2	HP263_Pit2	0.0021	0.003	1.428571
GP536_Pit2	HP274_Pit2	0.0106	0.0104	0.981132
GP536_Pit2	HP327_Pit2	0.0021	0.003	1.428571
GP536_Pit2	HP362-2_Pit2	0.0021	0.003	1.428571
GP536_Pit2	HP383_Pit2	0.0572	0.0423	0.73951
GP536_Pit2	HP396_Pit2	0	0.003	
GP536_Pit2	HP407_Pit2	0.0021	0.003	1.428571
GP536_Pit2	HP492_Pit2	0.0021	0.003	1.428571
GP536_Pit2	HP517-1_Pit2	0.0021	0.003	1.428571
GP536_Pit2	HP577_Pit2	0	0.003	
GP536_Pit2	W0128_Pit2	0.0572	0.0417	0.729021
GP536_Pit2	GP104_Pit2	0.0595	0.0423	0.710924
GP536_Pit2	GP124_Pit2	0.0595	0.0423	0.710924
GP536_Pit2	GP540_Pit2	0.0572	0.0423	0.73951
GP536_Pit2	Kasalath_Pit2	0.0595	0.0423	0.710924
GP536_Pit2	W3105-1_Pit2	0.0595	0.0423	0.710924
GP536_Pit2	GP295-1_Pit2	0.055	0.043	0.781818
GP536_Pit2	HP486_Pit2	0	0.003	
GP536_Pit2	GP62_Pit2	0.0579	0.0466	0.804836
GP640_Pit2	GP761-1_Pit2	0.055	0.043	0.781818
GP640_Pit2	DHX2_Pit2	0.0043	0.0042	0.976744
GP640_Pit2	GP551_Pit2	0.0043	0.0042	0.976744
GP640_Pit2	GP567_Pit2	0.055	0.043	0.781818
GP640_Pit2	GP669_Pit2	0	0	
GP640_Pit2	GP677_Pit2	0	0	
GP640_Pit2	HP13-2_Pit2	0.0549	0.0436	0.794171
GP640_Pit2	HP14_Pit2	0.0043	0.0036	0.837209
GP640_Pit2	HP38_Pit2	0.0021	0.003	1.428571
GP640_Pit2	HP44_Pit2	0	0	
GP640_Pit2	HP45_Pit2	0	0	
GP640_Pit2	HP48_Pit2	0.0043	0.0036	0.837209
GP640_Pit2	HP91-2_Pit2	0.055	0.043	0.781818
GP640_Pit2	HP98_Pit2	0.0043	0.0042	0.976744
GP640_Pit2	HP103_Pit2	0.0549	0.0436	0.794171
GP640_Pit2	HP390_Pit2	0.0572	0.0436	0.762238
GP640_Pit2	IL9_Pit2	0.0043	0.0042	0.976744
GP640_Pit2	Koshihikari_Pit2	0.0043	0.0042	0.976744
GP640_Pit2	KY131_Pit2	0.0043	0.0042	0.976744
GP640_Pit2	LG31_Pit2	0.0043	0.0042	0.976744
GP640_Pit2	UR28_Pit2	0.055	0.043	0.781818
GP640_Pit2	WYG7_Pit2	0.0572	0.0436	0.762238
GP640_Pit2	GLA4_Pit2	0.0549	0.0436	0.794171
GP640_Pit2	GP3_Pit2	0.0549	0.0436	0.794171
GP640_Pit2	GP22_Pit2	0.0607	0.0433	0.713344
GP640_Pit2	GP51_Pit2	0.0064	0.0006	0.09375
GP640_Pit2	GP772-1_Pit2	0.0572	0.0436	0.762238
GP640_Pit2	HP119_Pit2	0.0572	0.0436	0.762238

GP640_Pit2	HP263_Pit2	0.0572	0.0436	0.762238
GP640_Pit2	HP274_Pit2	0.0607	0.0433	0.713344
GP640_Pit2	HP327_Pit2	0.0572	0.0436	0.762238
GP640_Pit2	HP362-2_Pit2	0.0572	0.0436	0.762238
GP640_Pit2	HP383_Pit2	0.0064	0.0006	0.09375
GP640_Pit2	HP396_Pit2	0.0549	0.0436	0.794171
GP640_Pit2	HP407_Pit2	0.0572	0.0436	0.762238
GP640_Pit2	HP492_Pit2	0.0572	0.0436	0.762238
GP640_Pit2	HP517-1_Pit2	0.0572	0.0436	0.762238
GP640_Pit2	HP577_Pit2	0.0549	0.0436	0.794171
GP640_Pit2	W0128_Pit2	0.0064	0.0012	0.1875
GP640_Pit2	GP104_Pit2	0.0085	0.0018	0.211765
GP640_Pit2	GP124_Pit2	0.0085	0.0018	0.211765
GP640_Pit2	GP540_Pit2	0.0064	0.0006	0.09375
GP640_Pit2	Kasalath_Pit2	0.0085	0.0018	0.211765
GP640_Pit2	W3105-1_Pit2	0.0085	0.0018	0.211765
GP640_Pit2	GP295-1_Pit2	0	0	
GP640_Pit2	HP486_Pit2	0.0549	0.0436	0.794171
GP640_Pit2	GP62_Pit2	0.0215	0.011	0.511628
GP761-1_Pit2	DHX2_Pit2	0.0549	0.0449	0.817851
GP761-1_Pit2	GP551_Pit2	0.0549	0.0449	0.817851
GP761-1_Pit2	GP567_Pit2	0	0	
GP761-1_Pit2	GP669_Pit2	0.055	0.043	0.781818
GP761-1_Pit2	GP677_Pit2	0.055	0.043	0.781818
GP761-1_Pit2	HP13-2_Pit2	0	0.003	
GP761-1_Pit2	HP14_Pit2	0.055	0.0455	0.827273
GP761-1_Pit2	HP38_Pit2	0.0528	0.0397	0.751894
GP761-1_Pit2	HP44_Pit2	0.055	0.043	0.781818
GP761-1_Pit2	HP45_Pit2	0.055	0.043	0.781818
GP761-1_Pit2	HP48_Pit2	0.055	0.0455	0.827273
GP761-1_Pit2	HP91-2_Pit2	0	0	
GP761-1_Pit2	HP98_Pit2	0.0549	0.0449	0.817851
GP761-1_Pit2	HP103_Pit2	0	0.003	
GP761-1_Pit2	HP390_Pit2	0.0021	0.003	1.428571
GP761-1_Pit2	IL9_Pit2	0.0549	0.0449	0.817851
GP761-1_Pit2	Koshihikari_Pit2	0.0549	0.0449	0.817851
GP761-1_Pit2	KY131_Pit2	0.0549	0.0449	0.817851
GP761-1_Pit2	LG31_Pit2	0.0549	0.0449	0.817851
GP761-1_Pit2	UR28_Pit2	0	0	
GP761-1_Pit2	WYG7_Pit2	0.0021	0.003	1.428571
GP761-1_Pit2	GLA4_Pit2	0	0.003	
GP761-1_Pit2	GP3_Pit2	0	0.003	
GP761-1_Pit2	GP22_Pit2	0.0106	0.0104	0.981132
GP761-1_Pit2	GP51_Pit2	0.0572	0.0423	0.73951
GP761-1_Pit2	GP772-1_Pit2	0.0021	0.003	1.428571
GP761-1_Pit2	HP119_Pit2	0.0021	0.003	1.428571
GP761-1_Pit2	HP263_Pit2	0.0021	0.003	1.428571
GP761-1_Pit2	HP274_Pit2	0.0106	0.0104	0.981132
GP761-1_Pit2	HP327_Pit2	0.0021	0.003	1.428571
GP761-1_Pit2	HP362-2_Pit2	0.0021	0.003	1.428571
GP761-1_Pit2	HP383_Pit2	0.0572	0.0423	0.73951
GP761-1_Pit2	HP396_Pit2	0	0.003	
GP761-1_Pit2	HP407_Pit2	0.0021	0.003	1.428571
GP761-1_Pit2	HP492_Pit2	0.0021	0.003	1.428571
GP761-1_Pit2	HP517-1_Pit2	0.0021	0.003	1.428571
GP761-1_Pit2	HP577_Pit2	0	0.003	

GP761-1_Pit2	W0128_Pit2	0.0572	0.0417	0.729021
GP761-1_Pit2	GP104_Pit2	0.0595	0.0423	0.710924
GP761-1_Pit2	GP124_Pit2	0.0595	0.0423	0.710924
GP761-1_Pit2	GP540_Pit2	0.0572	0.0423	0.73951
GP761-1_Pit2	Kasalath_Pit2	0.0595	0.0423	0.710924
GP761-1_Pit2	W3105-1_Pit2	0.0595	0.0423	0.710924
GP761-1_Pit2	GP295-1_Pit2	0.055	0.043	0.781818
GP761-1_Pit2	HP486_Pit2	0	0.003	
GP761-1_Pit2	GP62_Pit2	0.0579	0.0466	0.804836
DHX2_Pit2	GP551_Pit2	0	0	
DHX2_Pit2	GP567_Pit2	0.0549	0.0449	0.817851
DHX2_Pit2	GP669_Pit2	0.0043	0.0042	0.976744
DHX2_Pit2	GP677_Pit2	0.0043	0.0042	0.976744
DHX2_Pit2	HP13-2_Pit2	0.0549	0.0456	0.830601
DHX2_Pit2	HP14_Pit2	0	0.0006	
DHX2_Pit2	HP38_Pit2	0.0021	0.0061	2.904762
DHX2_Pit2	HP44_Pit2	0.0043	0.0042	0.976744
DHX2_Pit2	HP45_Pit2	0.0043	0.0042	0.976744
DHX2_Pit2	HP48_Pit2	0	0.0006	
DHX2_Pit2	HP91-2_Pit2	0.0549	0.0449	0.817851
DHX2_Pit2	HP98_Pit2	0	0	
DHX2_Pit2	HP103_Pit2	0.0549	0.0456	0.830601
DHX2_Pit2	HP390_Pit2	0.0571	0.0456	0.798599
DHX2_Pit2	IL9_Pit2	0	0	
DHX2_Pit2	Koshihikari_Pit2	0	0	
DHX2_Pit2	KY131_Pit2	0	0	
DHX2_Pit2	LG31_Pit2	0	0	
DHX2_Pit2	UR28_Pit2	0.0549	0.0449	0.817851
DHX2_Pit2	WYG7_Pit2	0.0571	0.0456	0.798599
DHX2_Pit2	GLA4_Pit2	0.0549	0.0456	0.830601
DHX2_Pit2	GP3_Pit2	0.0549	0.0456	0.830601
DHX2_Pit2	GP22_Pit2	0.0607	0.0452	0.744646
DHX2_Pit2	GP51_Pit2	0.0064	0.0036	0.5625
DHX2_Pit2	GP772-1_Pit2	0.0571	0.0456	0.798599
DHX2_Pit2	HP119_Pit2	0.0571	0.0456	0.798599
DHX2_Pit2	HP263_Pit2	0.0571	0.0456	0.798599
DHX2_Pit2	HP274_Pit2	0.0607	0.0452	0.744646
DHX2_Pit2	HP327_Pit2	0.0571	0.0456	0.798599
DHX2_Pit2	HP362-2_Pit2	0.0571	0.0456	0.798599
DHX2_Pit2	HP383_Pit2	0.0064	0.0036	0.5625
DHX2_Pit2	HP396_Pit2	0.0549	0.0456	0.830601
DHX2_Pit2	HP407_Pit2	0.0571	0.0456	0.798599
DHX2_Pit2	HP492_Pit2	0.0571	0.0456	0.798599
DHX2_Pit2	HP517-1_Pit2	0.0571	0.0456	0.798599
DHX2_Pit2	HP577_Pit2	0.0549	0.0456	0.830601
DHX2_Pit2	W0128_Pit2	0.0064	0.0042	0.65625
DHX2_Pit2	GP104_Pit2	0.0085	0.0049	0.576471
DHX2_Pit2	GP124_Pit2	0.0085	0.0049	0.576471
DHX2_Pit2	GP540_Pit2	0.0064	0.0036	0.5625
DHX2_Pit2	Kasalath_Pit2	0.0085	0.0049	0.576471
DHX2_Pit2	W3105-1_Pit2	0.0085	0.0049	0.576471
DHX2_Pit2	GP295-1_Pit2	0.0043	0.0042	0.976744
DHX2_Pit2	HP486_Pit2	0.0549	0.0456	0.830601
DHX2_Pit2	GP62_Pit2	0.0215	0.0141	0.655814
GP551_Pit2	GP567_Pit2	0.0549	0.0449	0.817851
GP551_Pit2	GP669_Pit2	0.0043	0.0042	0.976744

GP551_Pit2	GP677_Pit2	0.0043	0.0042	0.976744
GP551_Pit2	HP13-2_Pit2	0.0549	0.0456	0.830601
GP551_Pit2	HP14_Pit2	0	0.0006	
GP551_Pit2	HP38_Pit2	0.0021	0.0061	2.904762
GP551_Pit2	HP44_Pit2	0.0043	0.0042	0.976744
GP551_Pit2	HP45_Pit2	0.0043	0.0042	0.976744
GP551_Pit2	HP48_Pit2	0	0.0006	
GP551_Pit2	HP91-2_Pit2	0.0549	0.0449	0.817851
GP551_Pit2	HP98_Pit2	0	0	
GP551_Pit2	HP103_Pit2	0.0549	0.0456	0.830601
GP551_Pit2	HP390_Pit2	0.0571	0.0456	0.798599
GP551_Pit2	IL9_Pit2	0	0	
GP551_Pit2	Koshihikari_Pit2	0	0	
GP551_Pit2	KY131_Pit2	0	0	
GP551_Pit2	LG31_Pit2	0	0	
GP551_Pit2	UR28_Pit2	0.0549	0.0449	0.817851
GP551_Pit2	WYG7_Pit2	0.0571	0.0456	0.798599
GP551_Pit2	GLA4_Pit2	0.0549	0.0456	0.830601
GP551_Pit2	GP3_Pit2	0.0549	0.0456	0.830601
GP551_Pit2	GP22_Pit2	0.0607	0.0452	0.744646
GP551_Pit2	GP51_Pit2	0.0064	0.0036	0.5625
GP551_Pit2	GP772-1_Pit2	0.0571	0.0456	0.798599
GP551_Pit2	HP119_Pit2	0.0571	0.0456	0.798599
GP551_Pit2	HP263_Pit2	0.0571	0.0456	0.798599
GP551_Pit2	HP274_Pit2	0.0607	0.0452	0.744646
GP551_Pit2	HP327_Pit2	0.0571	0.0456	0.798599
GP551_Pit2	HP362-2_Pit2	0.0571	0.0456	0.798599
GP551_Pit2	HP383_Pit2	0.0064	0.0036	0.5625
GP551_Pit2	HP396_Pit2	0.0549	0.0456	0.830601
GP551_Pit2	HP407_Pit2	0.0571	0.0456	0.798599
GP551_Pit2	HP492_Pit2	0.0571	0.0456	0.798599
GP551_Pit2	HP517-1_Pit2	0.0571	0.0456	0.798599
GP551_Pit2	HP577_Pit2	0.0549	0.0456	0.830601
GP551_Pit2	W0128_Pit2	0.0064	0.0042	0.65625
GP551_Pit2	GP104_Pit2	0.0085	0.0049	0.576471
GP551_Pit2	GP124_Pit2	0.0085	0.0049	0.576471
GP551_Pit2	GP540_Pit2	0.0064	0.0036	0.5625
GP551_Pit2	Kasalath_Pit2	0.0085	0.0049	0.576471
GP551_Pit2	W3105-1_Pit2	0.0085	0.0049	0.576471
GP551_Pit2	GP295-1_Pit2	0.0043	0.0042	0.976744
GP551_Pit2	HP486_Pit2	0.0549	0.0456	0.830601
GP551_Pit2	GP62_Pit2	0.0215	0.0141	0.655814
GP567_Pit2	GP669_Pit2	0.055	0.043	0.781818
GP567_Pit2	GP677_Pit2	0.055	0.043	0.781818
GP567_Pit2	HP13-2_Pit2	0	0.003	
GP567_Pit2	HP14_Pit2	0.055	0.0455	0.827273
GP567_Pit2	HP38_Pit2	0.0528	0.0397	0.751894
GP567_Pit2	HP44_Pit2	0.055	0.043	0.781818
GP567_Pit2	HP45_Pit2	0.055	0.043	0.781818
GP567_Pit2	HP48_Pit2	0.055	0.0455	0.827273
GP567_Pit2	HP91-2_Pit2	0	0	
GP567_Pit2	HP98_Pit2	0.0549	0.0449	0.817851
GP567_Pit2	HP103_Pit2	0	0.003	
GP567_Pit2	HP390_Pit2	0.0021	0.003	1.428571
GP567_Pit2	IL9_Pit2	0.0549	0.0449	0.817851
GP567_Pit2	Koshihikari_Pit2	0.0549	0.0449	0.817851

GP567_Pit2	KY131_Pit2	0.0549	0.0449	0.817851
GP567_Pit2	LG31_Pit2	0.0549	0.0449	0.817851
GP567_Pit2	UR28_Pit2	0	0	
GP567_Pit2	WYG7_Pit2	0.0021	0.003	1.428571
GP567_Pit2	GLA4_Pit2	0	0.003	
GP567_Pit2	GP3_Pit2	0	0.003	
GP567_Pit2	GP22_Pit2	0.0106	0.0104	0.981132
GP567_Pit2	GP51_Pit2	0.0572	0.0423	0.73951
GP567_Pit2	GP772-1_Pit2	0.0021	0.003	1.428571
GP567_Pit2	HP119_Pit2	0.0021	0.003	1.428571
GP567_Pit2	HP263_Pit2	0.0021	0.003	1.428571
GP567_Pit2	HP274_Pit2	0.0106	0.0104	0.981132
GP567_Pit2	HP327_Pit2	0.0021	0.003	1.428571
GP567_Pit2	HP362-2_Pit2	0.0021	0.003	1.428571
GP567_Pit2	HP383_Pit2	0.0572	0.0423	0.73951
GP567_Pit2	HP396_Pit2	0	0.003	
GP567_Pit2	HP407_Pit2	0.0021	0.003	1.428571
GP567_Pit2	HP492_Pit2	0.0021	0.003	1.428571
GP567_Pit2	HP517-1_Pit2	0.0021	0.003	1.428571
GP567_Pit2	HP577_Pit2	0	0.003	
GP567_Pit2	W0128_Pit2	0.0572	0.0417	0.729021
GP567_Pit2	GP104_Pit2	0.0595	0.0423	0.710924
GP567_Pit2	GP124_Pit2	0.0595	0.0423	0.710924
GP567_Pit2	GP540_Pit2	0.0572	0.0423	0.73951
GP567_Pit2	Kasalath_Pit2	0.0595	0.0423	0.710924
GP567_Pit2	W3105-1_Pit2	0.0595	0.0423	0.710924
GP567_Pit2	GP295-1_Pit2	0.055	0.043	0.781818
GP567_Pit2	HP486_Pit2	0	0.003	
GP567_Pit2	GP62_Pit2	0.0579	0.0466	0.804836
GP669_Pit2	GP677_Pit2	0	0	
GP669_Pit2	HP13-2_Pit2	0.0549	0.0436	0.794171
GP669_Pit2	HP14_Pit2	0.0043	0.0036	0.837209
GP669_Pit2	HP38_Pit2	0.0021	0.003	1.428571
GP669_Pit2	HP44_Pit2	0	0	
GP669_Pit2	HP45_Pit2	0	0	
GP669_Pit2	HP48_Pit2	0.0043	0.0036	0.837209
GP669_Pit2	HP91-2_Pit2	0.055	0.043	0.781818
GP669_Pit2	HP98_Pit2	0.0043	0.0042	0.976744
GP669_Pit2	HP103_Pit2	0.0549	0.0436	0.794171
GP669_Pit2	HP390_Pit2	0.0572	0.0436	0.762238
GP669_Pit2	IL9_Pit2	0.0043	0.0042	0.976744
GP669_Pit2	Koshihikari_Pit2	0.0043	0.0042	0.976744
GP669_Pit2	KY131_Pit2	0.0043	0.0042	0.976744
GP669_Pit2	LG31_Pit2	0.0043	0.0042	0.976744
GP669_Pit2	UR28_Pit2	0.055	0.043	0.781818
GP669_Pit2	WYG7_Pit2	0.0572	0.0436	0.762238
GP669_Pit2	GLA4_Pit2	0.0549	0.0436	0.794171
GP669_Pit2	GP3_Pit2	0.0549	0.0436	0.794171
GP669_Pit2	GP22_Pit2	0.0607	0.0433	0.713344
GP669_Pit2	GP51_Pit2	0.0064	0.0006	0.09375
GP669_Pit2	GP772-1_Pit2	0.0572	0.0436	0.762238
GP669_Pit2	HP119_Pit2	0.0572	0.0436	0.762238
GP669_Pit2	HP263_Pit2	0.0572	0.0436	0.762238
GP669_Pit2	HP274_Pit2	0.0607	0.0433	0.713344
GP669_Pit2	HP327_Pit2	0.0572	0.0436	0.762238
GP669_Pit2	HP362-2_Pit2	0.0572	0.0436	0.762238

GP669_Pit2	HP383_Pit2	0.0064	0.0006	0.09375
GP669_Pit2	HP396_Pit2	0.0549	0.0436	0.794171
GP669_Pit2	HP407_Pit2	0.0572	0.0436	0.762238
GP669_Pit2	HP492_Pit2	0.0572	0.0436	0.762238
GP669_Pit2	HP517-1_Pit2	0.0572	0.0436	0.762238
GP669_Pit2	HP577_Pit2	0.0549	0.0436	0.794171
GP669_Pit2	W0128_Pit2	0.0064	0.0012	0.1875
GP669_Pit2	GP104_Pit2	0.0085	0.0018	0.211765
GP669_Pit2	GP124_Pit2	0.0085	0.0018	0.211765
GP669_Pit2	GP540_Pit2	0.0064	0.0006	0.09375
GP669_Pit2	Kasalath_Pit2	0.0085	0.0018	0.211765
GP669_Pit2	W3105-1_Pit2	0.0085	0.0018	0.211765
GP669_Pit2	GP295-1_Pit2	0	0	
GP669_Pit2	HP486_Pit2	0.0549	0.0436	0.794171
GP669_Pit2	GP62_Pit2	0.0215	0.011	0.511628
GP677_Pit2	HP13-2_Pit2	0.0549	0.0436	0.794171
GP677_Pit2	HP14_Pit2	0.0043	0.0036	0.837209
GP677_Pit2	HP38_Pit2	0.0021	0.003	1.428571
GP677_Pit2	HP44_Pit2	0	0	
GP677_Pit2	HP45_Pit2	0	0	
GP677_Pit2	HP48_Pit2	0.0043	0.0036	0.837209
GP677_Pit2	HP91-2_Pit2	0.055	0.043	0.781818
GP677_Pit2	HP98_Pit2	0.0043	0.0042	0.976744
GP677_Pit2	HP103_Pit2	0.0549	0.0436	0.794171
GP677_Pit2	HP390_Pit2	0.0572	0.0436	0.762238
GP677_Pit2	IL9_Pit2	0.0043	0.0042	0.976744
GP677_Pit2	Koshihikari_Pit2	0.0043	0.0042	0.976744
GP677_Pit2	KY131_Pit2	0.0043	0.0042	0.976744
GP677_Pit2	LG31_Pit2	0.0043	0.0042	0.976744
GP677_Pit2	UR28_Pit2	0.055	0.043	0.781818
GP677_Pit2	WYG7_Pit2	0.0572	0.0436	0.762238
GP677_Pit2	GLA4_Pit2	0.0549	0.0436	0.794171
GP677_Pit2	GP3_Pit2	0.0549	0.0436	0.794171
GP677_Pit2	GP22_Pit2	0.0607	0.0433	0.713344
GP677_Pit2	GP51_Pit2	0.0064	0.0006	0.09375
GP677_Pit2	GP772-1_Pit2	0.0572	0.0436	0.762238
GP677_Pit2	HP119_Pit2	0.0572	0.0436	0.762238
GP677_Pit2	HP263_Pit2	0.0572	0.0436	0.762238
GP677_Pit2	HP274_Pit2	0.0607	0.0433	0.713344
GP677_Pit2	HP327_Pit2	0.0572	0.0436	0.762238
GP677_Pit2	HP362-2_Pit2	0.0572	0.0436	0.762238
GP677_Pit2	HP383_Pit2	0.0064	0.0006	0.09375
GP677_Pit2	HP396_Pit2	0.0549	0.0436	0.794171
GP677_Pit2	HP407_Pit2	0.0572	0.0436	0.762238
GP677_Pit2	HP492_Pit2	0.0572	0.0436	0.762238
GP677_Pit2	HP517-1_Pit2	0.0572	0.0436	0.762238
GP677_Pit2	HP577_Pit2	0.0549	0.0436	0.794171
GP677_Pit2	W0128_Pit2	0.0064	0.0012	0.1875
GP677_Pit2	GP104_Pit2	0.0085	0.0018	0.211765
GP677_Pit2	GP124_Pit2	0.0085	0.0018	0.211765
GP677_Pit2	GP540_Pit2	0.0064	0.0006	0.09375
GP677_Pit2	Kasalath_Pit2	0.0085	0.0018	0.211765
GP677_Pit2	W3105-1_Pit2	0.0085	0.0018	0.211765
GP677_Pit2	GP295-1_Pit2	0	0	
GP677_Pit2	HP486_Pit2	0.0549	0.0436	0.794171
GP677_Pit2	GP62_Pit2	0.0215	0.011	0.511628

HP13-2_Pit2	HP14_Pit2	0.0549	0.0462	0.84153
HP13-2_Pit2	HP38_Pit2	0.0527	0.043	0.815939
HP13-2_Pit2	HP44_Pit2	0.0549	0.0436	0.794171
HP13-2_Pit2	HP45_Pit2	0.0549	0.0436	0.794171
HP13-2_Pit2	HP48_Pit2	0.0549	0.0462	0.84153
HP13-2_Pit2	HP91-2_Pit2	0	0.003	
HP13-2_Pit2	HP98_Pit2	0.0549	0.0456	0.830601
HP13-2_Pit2	HP103_Pit2	0	0	
HP13-2_Pit2	HP390_Pit2	0.0021	0.0036	1.714286
HP13-2_Pit2	IL9_Pit2	0.0549	0.0456	0.830601
HP13-2_Pit2	Koshihikari_Pit2	0.0549	0.0456	0.830601
HP13-2_Pit2	KY131_Pit2	0.0549	0.0456	0.830601
HP13-2_Pit2	LG31_Pit2	0.0549	0.0456	0.830601
HP13-2_Pit2	UR28_Pit2	0	0.003	
HP13-2_Pit2	WYG7_Pit2	0.0021	0.0036	1.714286
HP13-2_Pit2	GLA4_Pit2	0	0	
HP13-2_Pit2	GP3_Pit2	0	0	
HP13-2_Pit2	GP22_Pit2	0.0106	0.011	1.037736
HP13-2_Pit2	GP51_Pit2	0.0572	0.043	0.751748
HP13-2_Pit2	GP772-1_Pit2	0.0021	0.0036	1.714286
HP13-2_Pit2	HP119_Pit2	0.0021	0.0036	1.714286
HP13-2_Pit2	HP263_Pit2	0.0021	0.0036	1.714286
HP13-2_Pit2	HP274_Pit2	0.0106	0.011	1.037736
HP13-2_Pit2	HP327_Pit2	0.0021	0.0036	1.714286
HP13-2_Pit2	HP362-2_Pit2	0.0021	0.0036	1.714286
HP13-2_Pit2	HP383_Pit2	0.0572	0.043	0.751748
HP13-2_Pit2	HP396_Pit2	0	0	
HP13-2_Pit2	HP407_Pit2	0.0021	0.0036	1.714286
HP13-2_Pit2	HP492_Pit2	0.0021	0.0036	1.714286
HP13-2_Pit2	HP517-1_Pit2	0.0021	0.0036	1.714286
HP13-2_Pit2	HP577_Pit2	0	0	
HP13-2_Pit2	W0128_Pit2	0.0572	0.0423	0.73951
HP13-2_Pit2	GP104_Pit2	0.0594	0.043	0.723906
HP13-2_Pit2	GP124_Pit2	0.0594	0.043	0.723906
HP13-2_Pit2	GP540_Pit2	0.0572	0.043	0.751748
HP13-2_Pit2	Kasalath_Pit2	0.0594	0.043	0.723906
HP13-2_Pit2	W3105-1_Pit2	0.0594	0.043	0.723906
HP13-2_Pit2	GP295-1_Pit2	0.0549	0.0436	0.794171
HP13-2_Pit2	HP486_Pit2	0	0	
HP13-2_Pit2	GP62_Pit2	0.0578	0.0473	0.818339
HP14_Pit2	HP38_Pit2	0.0021	0.0055	2.619048
HP14_Pit2	HP44_Pit2	0.0043	0.0036	0.837209
HP14_Pit2	HP45_Pit2	0.0043	0.0036	0.837209
HP14_Pit2	HP48_Pit2	0	0	
HP14_Pit2	HP91-2_Pit2	0.055	0.0455	0.827273
HP14_Pit2	HP98_Pit2	0	0.0006	
HP14_Pit2	HP103_Pit2	0.0549	0.0462	0.84153
HP14_Pit2	HP390_Pit2	0.0572	0.0462	0.807692
HP14_Pit2	IL9_Pit2	0	0.0006	
HP14_Pit2	Koshihikari_Pit2	0	0.0006	
HP14_Pit2	KY131_Pit2	0	0.0006	
HP14_Pit2	LG31_Pit2	0	0.0006	
HP14_Pit2	UR28_Pit2	0.055	0.0455	0.827273
HP14_Pit2	WYG7_Pit2	0.0572	0.0462	0.807692
HP14_Pit2	GLA4_Pit2	0.0549	0.0462	0.84153
HP14_Pit2	GP3_Pit2	0.0549	0.0462	0.84153

HP14_Pit2	GP22_Pit2	0.0607	0.0458	0.75453
HP14_Pit2	GP51_Pit2	0.0064	0.003	0.46875
HP14_Pit2	GP772-1_Pit2	0.0572	0.0462	0.807692
HP14_Pit2	HP119_Pit2	0.0572	0.0462	0.807692
HP14_Pit2	HP263_Pit2	0.0572	0.0462	0.807692
HP14_Pit2	HP274_Pit2	0.0607	0.0458	0.75453
HP14_Pit2	HP327_Pit2	0.0572	0.0462	0.807692
HP14_Pit2	HP362-2_Pit2	0.0572	0.0462	0.807692
HP14_Pit2	HP383_Pit2	0.0064	0.003	0.46875
HP14_Pit2	HP396_Pit2	0.0549	0.0462	0.84153
HP14_Pit2	HP407_Pit2	0.0572	0.0462	0.807692
HP14_Pit2	HP492_Pit2	0.0572	0.0462	0.807692
HP14_Pit2	HP517-1_Pit2	0.0572	0.0462	0.807692
HP14_Pit2	HP577_Pit2	0.0549	0.0462	0.84153
HP14_Pit2	W0128_Pit2	0.0064	0.0036	0.5625
HP14_Pit2	GP104_Pit2	0.0085	0.0042	0.494118
HP14_Pit2	GP124_Pit2	0.0085	0.0042	0.494118
HP14_Pit2	GP540_Pit2	0.0064	0.003	0.46875
HP14_Pit2	Kasalath_Pit2	0.0085	0.0042	0.494118
HP14_Pit2	W3105-1_Pit2	0.0085	0.0042	0.494118
HP14_Pit2	GP295-1_Pit2	0.0043	0.0036	0.837209
HP14_Pit2	HP486_Pit2	0.0549	0.0462	0.84153
HP14_Pit2	GP62_Pit2	0.0215	0.0134	0.623256
HP38_Pit2	HP44_Pit2	0.0021	0.003	1.428571
HP38_Pit2	HP45_Pit2	0.0021	0.003	1.428571
HP38_Pit2	HP48_Pit2	0.0021	0.0055	2.619048
HP38_Pit2	HP91-2_Pit2	0.0528	0.0397	0.751894
HP38_Pit2	HP98_Pit2	0.0021	0.0061	2.904762
HP38_Pit2	HP103_Pit2	0.0527	0.043	0.815939
HP38_Pit2	HP390_Pit2	0.055	0.043	0.781818
HP38_Pit2	IL9_Pit2	0.0021	0.0061	2.904762
HP38_Pit2	Koshihikari_Pit2	0.0021	0.0061	2.904762
HP38_Pit2	KY131_Pit2	0.0021	0.0061	2.904762
HP38_Pit2	LG31_Pit2	0.0021	0.0061	2.904762
HP38_Pit2	UR28_Pit2	0.0528	0.0397	0.751894
HP38_Pit2	WYG7_Pit2	0.055	0.043	0.781818
HP38_Pit2	GLA4_Pit2	0.0527	0.043	0.815939
HP38_Pit2	GP3_Pit2	0.0527	0.043	0.815939
HP38_Pit2	GP22_Pit2	0.0586	0.0426	0.726962
HP38_Pit2	GP51_Pit2	0.0043	0.0024	0.55814
HP38_Pit2	GP772-1_Pit2	0.055	0.043	0.781818
HP38_Pit2	HP119_Pit2	0.055	0.043	0.781818
HP38_Pit2	HP263_Pit2	0.055	0.043	0.781818
HP38_Pit2	HP274_Pit2	0.0586	0.0426	0.726962
HP38_Pit2	HP327_Pit2	0.055	0.043	0.781818
HP38_Pit2	HP362-2_Pit2	0.055	0.043	0.781818
HP38_Pit2	HP383_Pit2	0.0043	0.0024	0.55814
HP38_Pit2	HP396_Pit2	0.0527	0.043	0.815939
HP38_Pit2	HP407_Pit2	0.055	0.043	0.781818
HP38_Pit2	HP492_Pit2	0.055	0.043	0.781818
HP38_Pit2	HP517-1_Pit2	0.055	0.043	0.781818
HP38_Pit2	HP577_Pit2	0.0527	0.043	0.815939
HP38_Pit2	W0128_Pit2	0.0043	0.0018	0.418605
HP38_Pit2	GP104_Pit2	0.0064	0.0024	0.375
HP38_Pit2	GP124_Pit2	0.0064	0.0024	0.375
HP38_Pit2	GP540_Pit2	0.0043	0.0024	0.55814

HP38_Pit2	Kasalath_Pit2	0.0064	0.0024	0.375
HP38_Pit2	W3105-1_Pit2	0.0064	0.0024	0.375
HP38_Pit2	GP295-1_Pit2	0.0021	0.003	1.428571
HP38_Pit2	HP486_Pit2	0.0527	0.043	0.815939
HP38_Pit2	GP62_Pit2	0.0194	0.0116	0.597938
HP44_Pit2	HP45_Pit2	0	0	
HP44_Pit2	HP48_Pit2	0.0043	0.0036	0.837209
HP44_Pit2	HP91-2_Pit2	0.055	0.043	0.781818
HP44_Pit2	HP98_Pit2	0.0043	0.0042	0.976744
HP44_Pit2	HP103_Pit2	0.0549	0.0436	0.794171
HP44_Pit2	HP390_Pit2	0.0572	0.0436	0.762238
HP44_Pit2	IL9_Pit2	0.0043	0.0042	0.976744
HP44_Pit2	Koshihikari_Pit2	0.0043	0.0042	0.976744
HP44_Pit2	KY131_Pit2	0.0043	0.0042	0.976744
HP44_Pit2	LG31_Pit2	0.0043	0.0042	0.976744
HP44_Pit2	UR28_Pit2	0.055	0.043	0.781818
HP44_Pit2	WYG7_Pit2	0.0572	0.0436	0.762238
HP44_Pit2	GLA4_Pit2	0.0549	0.0436	0.794171
HP44_Pit2	GP3_Pit2	0.0549	0.0436	0.794171
HP44_Pit2	GP22_Pit2	0.0607	0.0433	0.713344
HP44_Pit2	GP51_Pit2	0.0064	0.0006	0.09375
HP44_Pit2	GP772-1_Pit2	0.0572	0.0436	0.762238
HP44_Pit2	HP119_Pit2	0.0572	0.0436	0.762238
HP44_Pit2	HP263_Pit2	0.0572	0.0436	0.762238
HP44_Pit2	HP274_Pit2	0.0607	0.0433	0.713344
HP44_Pit2	HP327_Pit2	0.0572	0.0436	0.762238
HP44_Pit2	HP362-2_Pit2	0.0572	0.0436	0.762238
HP44_Pit2	HP383_Pit2	0.0064	0.0006	0.09375
HP44_Pit2	HP396_Pit2	0.0549	0.0436	0.794171
HP44_Pit2	HP407_Pit2	0.0572	0.0436	0.762238
HP44_Pit2	HP492_Pit2	0.0572	0.0436	0.762238
HP44_Pit2	HP517-1_Pit2	0.0572	0.0436	0.762238
HP44_Pit2	HP577_Pit2	0.0549	0.0436	0.794171
HP44_Pit2	W0128_Pit2	0.0064	0.0012	0.1875
HP44_Pit2	GP104_Pit2	0.0085	0.0018	0.211765
HP44_Pit2	GP124_Pit2	0.0085	0.0018	0.211765
HP44_Pit2	GP540_Pit2	0.0064	0.0006	0.09375
HP44_Pit2	Kasalath_Pit2	0.0085	0.0018	0.211765
HP44_Pit2	W3105-1_Pit2	0.0085	0.0018	0.211765
HP44_Pit2	GP295-1_Pit2	0	0	
HP44_Pit2	HP486_Pit2	0.0549	0.0436	0.794171
HP44_Pit2	GP62_Pit2	0.0215	0.011	0.511628
HP45_Pit2	HP48_Pit2	0.0043	0.0036	0.837209
HP45_Pit2	HP91-2_Pit2	0.055	0.043	0.781818
HP45_Pit2	HP98_Pit2	0.0043	0.0042	0.976744
HP45_Pit2	HP103_Pit2	0.0549	0.0436	0.794171
HP45_Pit2	HP390_Pit2	0.0572	0.0436	0.762238
HP45_Pit2	IL9_Pit2	0.0043	0.0042	0.976744
HP45_Pit2	Koshihikari_Pit2	0.0043	0.0042	0.976744
HP45_Pit2	KY131_Pit2	0.0043	0.0042	0.976744
HP45_Pit2	LG31_Pit2	0.0043	0.0042	0.976744
HP45_Pit2	UR28_Pit2	0.055	0.043	0.781818
HP45_Pit2	WYG7_Pit2	0.0572	0.0436	0.762238
HP45_Pit2	GLA4_Pit2	0.0549	0.0436	0.794171
HP45_Pit2	GP3_Pit2	0.0549	0.0436	0.794171
HP45_Pit2	GP22_Pit2	0.0607	0.0433	0.713344

HP45_Pit2	GP51_Pit2	0.0064	0.0006	0.09375
HP45_Pit2	GP772-1_Pit2	0.0572	0.0436	0.762238
HP45_Pit2	HP119_Pit2	0.0572	0.0436	0.762238
HP45_Pit2	HP263_Pit2	0.0572	0.0436	0.762238
HP45_Pit2	HP274_Pit2	0.0607	0.0433	0.713344
HP45_Pit2	HP327_Pit2	0.0572	0.0436	0.762238
HP45_Pit2	HP362-2_Pit2	0.0572	0.0436	0.762238
HP45_Pit2	HP383_Pit2	0.0064	0.0006	0.09375
HP45_Pit2	HP396_Pit2	0.0549	0.0436	0.794171
HP45_Pit2	HP407_Pit2	0.0572	0.0436	0.762238
HP45_Pit2	HP492_Pit2	0.0572	0.0436	0.762238
HP45_Pit2	HP517-1_Pit2	0.0572	0.0436	0.762238
HP45_Pit2	HP577_Pit2	0.0549	0.0436	0.794171
HP45_Pit2	W0128_Pit2	0.0064	0.0012	0.1875
HP45_Pit2	GP104_Pit2	0.0085	0.0018	0.211765
HP45_Pit2	GP124_Pit2	0.0085	0.0018	0.211765
HP45_Pit2	GP540_Pit2	0.0064	0.0006	0.09375
HP45_Pit2	Kasalath_Pit2	0.0085	0.0018	0.211765
HP45_Pit2	W3105-1_Pit2	0.0085	0.0018	0.211765
HP45_Pit2	GP295-1_Pit2	0	0	
HP45_Pit2	HP486_Pit2	0.0549	0.0436	0.794171
HP45_Pit2	GP62_Pit2	0.0215	0.011	0.511628
HP48_Pit2	HP91-2_Pit2	0.055	0.0455	0.827273
HP48_Pit2	HP98_Pit2	0	0.0006	
HP48_Pit2	HP103_Pit2	0.0549	0.0462	0.84153
HP48_Pit2	HP390_Pit2	0.0572	0.0462	0.807692
HP48_Pit2	IL9_Pit2	0	0.0006	
HP48_Pit2	Koshihikari_Pit2	0	0.0006	
HP48_Pit2	KY131_Pit2	0	0.0006	
HP48_Pit2	LG31_Pit2	0	0.0006	
HP48_Pit2	UR28_Pit2	0.055	0.0455	0.827273
HP48_Pit2	WYG7_Pit2	0.0572	0.0462	0.807692
HP48_Pit2	GLA4_Pit2	0.0549	0.0462	0.84153
HP48_Pit2	GP3_Pit2	0.0549	0.0462	0.84153
HP48_Pit2	GP22_Pit2	0.0607	0.0458	0.75453
HP48_Pit2	GP51_Pit2	0.0064	0.003	0.46875
HP48_Pit2	GP772-1_Pit2	0.0572	0.0462	0.807692
HP48_Pit2	HP119_Pit2	0.0572	0.0462	0.807692
HP48_Pit2	HP263_Pit2	0.0572	0.0462	0.807692
HP48_Pit2	HP274_Pit2	0.0607	0.0458	0.75453
HP48_Pit2	HP327_Pit2	0.0572	0.0462	0.807692
HP48_Pit2	HP362-2_Pit2	0.0572	0.0462	0.807692
HP48_Pit2	HP383_Pit2	0.0064	0.003	0.46875
HP48_Pit2	HP396_Pit2	0.0549	0.0462	0.84153
HP48_Pit2	HP407_Pit2	0.0572	0.0462	0.807692
HP48_Pit2	HP492_Pit2	0.0572	0.0462	0.807692
HP48_Pit2	HP517-1_Pit2	0.0572	0.0462	0.807692
HP48_Pit2	HP577_Pit2	0.0549	0.0462	0.84153
HP48_Pit2	W0128_Pit2	0.0064	0.0036	0.5625
HP48_Pit2	GP104_Pit2	0.0085	0.0042	0.494118
HP48_Pit2	GP124_Pit2	0.0085	0.0042	0.494118
HP48_Pit2	GP540_Pit2	0.0064	0.003	0.46875
HP48_Pit2	Kasalath_Pit2	0.0085	0.0042	0.494118
HP48_Pit2	W3105-1_Pit2	0.0085	0.0042	0.494118
HP48_Pit2	GP295-1_Pit2	0.0043	0.0036	0.837209
HP48_Pit2	HP486_Pit2	0.0549	0.0462	0.84153

HP48_Pit2	GP62_Pit2	0.0215	0.0134	0.623256
HP91-2_Pit2	HP98_Pit2	0.0549	0.0449	0.817851
HP91-2_Pit2	HP103_Pit2	0	0.003	
HP91-2_Pit2	HP390_Pit2	0.0021	0.003	1.428571
HP91-2_Pit2	IL9_Pit2	0.0549	0.0449	0.817851
HP91-2_Pit2	Koshihikari_Pit2	0.0549	0.0449	0.817851
HP91-2_Pit2	KY131_Pit2	0.0549	0.0449	0.817851
HP91-2_Pit2	LG31_Pit2	0.0549	0.0449	0.817851
HP91-2_Pit2	UR28_Pit2	0	0	
HP91-2_Pit2	WYG7_Pit2	0.0021	0.003	1.428571
HP91-2_Pit2	GLA4_Pit2	0	0.003	
HP91-2_Pit2	GP3_Pit2	0	0.003	
HP91-2_Pit2	GP22_Pit2	0.0106	0.0104	0.981132
HP91-2_Pit2	GP51_Pit2	0.0572	0.0423	0.73951
HP91-2_Pit2	GP772-1_Pit2	0.0021	0.003	1.428571
HP91-2_Pit2	HP119_Pit2	0.0021	0.003	1.428571
HP91-2_Pit2	HP263_Pit2	0.0021	0.003	1.428571
HP91-2_Pit2	HP274_Pit2	0.0106	0.0104	0.981132
HP91-2_Pit2	HP327_Pit2	0.0021	0.003	1.428571
HP91-2_Pit2	HP362-2_Pit2	0.0021	0.003	1.428571
HP91-2_Pit2	HP383_Pit2	0.0572	0.0423	0.73951
HP91-2_Pit2	HP396_Pit2	0	0.003	
HP91-2_Pit2	HP407_Pit2	0.0021	0.003	1.428571
HP91-2_Pit2	HP492_Pit2	0.0021	0.003	1.428571
HP91-2_Pit2	HP517-1_Pit2	0.0021	0.003	1.428571
HP91-2_Pit2	HP577_Pit2	0	0.003	
HP91-2_Pit2	W0128_Pit2	0.0572	0.0417	0.729021
HP91-2_Pit2	GP104_Pit2	0.0595	0.0423	0.710924
HP91-2_Pit2	GP124_Pit2	0.0595	0.0423	0.710924
HP91-2_Pit2	GP540_Pit2	0.0572	0.0423	0.73951
HP91-2_Pit2	Kasalath_Pit2	0.0595	0.0423	0.710924
HP91-2_Pit2	W3105-1_Pit2	0.0595	0.0423	0.710924
HP91-2_Pit2	GP295-1_Pit2	0.055	0.043	0.781818
HP91-2_Pit2	HP486_Pit2	0	0.003	
HP91-2_Pit2	GP62_Pit2	0.0579	0.0466	0.804836
HP98_Pit2	HP103_Pit2	0.0549	0.0456	0.830601
HP98_Pit2	HP390_Pit2	0.0571	0.0456	0.798599
HP98_Pit2	IL9_Pit2	0	0	
HP98_Pit2	Koshihikari_Pit2	0	0	
HP98_Pit2	KY131_Pit2	0	0	
HP98_Pit2	LG31_Pit2	0	0	
HP98_Pit2	UR28_Pit2	0.0549	0.0449	0.817851
HP98_Pit2	WYG7_Pit2	0.0571	0.0456	0.798599
HP98_Pit2	GLA4_Pit2	0.0549	0.0456	0.830601
HP98_Pit2	GP3_Pit2	0.0549	0.0456	0.830601
HP98_Pit2	GP22_Pit2	0.0607	0.0452	0.744646
HP98_Pit2	GP51_Pit2	0.0064	0.0036	0.5625
HP98_Pit2	GP772-1_Pit2	0.0571	0.0456	0.798599
HP98_Pit2	HP119_Pit2	0.0571	0.0456	0.798599
HP98_Pit2	HP263_Pit2	0.0571	0.0456	0.798599
HP98_Pit2	HP274_Pit2	0.0607	0.0452	0.744646
HP98_Pit2	HP327_Pit2	0.0571	0.0456	0.798599
HP98_Pit2	HP362-2_Pit2	0.0571	0.0456	0.798599
HP98_Pit2	HP383_Pit2	0.0064	0.0036	0.5625
HP98_Pit2	HP396_Pit2	0.0549	0.0456	0.830601
HP98_Pit2	HP407_Pit2	0.0571	0.0456	0.798599

HP98_Pit2	HP492_Pit2	0.0571	0.0456	0.798599
HP98_Pit2	HP517-1_Pit2	0.0571	0.0456	0.798599
HP98_Pit2	HP577_Pit2	0.0549	0.0456	0.830601
HP98_Pit2	W0128_Pit2	0.0064	0.0042	0.65625
HP98_Pit2	GP104_Pit2	0.0085	0.0049	0.576471
HP98_Pit2	GP124_Pit2	0.0085	0.0049	0.576471
HP98_Pit2	GP540_Pit2	0.0064	0.0036	0.5625
HP98_Pit2	Kasalath_Pit2	0.0085	0.0049	0.576471
HP98_Pit2	W3105-1_Pit2	0.0085	0.0049	0.576471
HP98_Pit2	GP295-1_Pit2	0.0043	0.0042	0.976744
HP98_Pit2	HP486_Pit2	0.0549	0.0456	0.830601
HP98_Pit2	GP62_Pit2	0.0215	0.0141	0.655814
HP103_Pit2	HP390_Pit2	0.0021	0.0036	1.714286
HP103_Pit2	IL9_Pit2	0.0549	0.0456	0.830601
HP103_Pit2	Koshihikari_Pit2	0.0549	0.0456	0.830601
HP103_Pit2	KY131_Pit2	0.0549	0.0456	0.830601
HP103_Pit2	LG31_Pit2	0.0549	0.0456	0.830601
HP103_Pit2	UR28_Pit2	0	0.003	
HP103_Pit2	WYG7_Pit2	0.0021	0.0036	1.714286
HP103_Pit2	GLA4_Pit2	0	0	
HP103_Pit2	GP3_Pit2	0	0	
HP103_Pit2	GP22_Pit2	0.0106	0.011	1.037736
HP103_Pit2	GP51_Pit2	0.0572	0.043	0.751748
HP103_Pit2	GP772-1_Pit2	0.0021	0.0036	1.714286
HP103_Pit2	HP119_Pit2	0.0021	0.0036	1.714286
HP103_Pit2	HP263_Pit2	0.0021	0.0036	1.714286
HP103_Pit2	HP274_Pit2	0.0106	0.011	1.037736
HP103_Pit2	HP327_Pit2	0.0021	0.0036	1.714286
HP103_Pit2	HP362-2_Pit2	0.0021	0.0036	1.714286
HP103_Pit2	HP383_Pit2	0.0572	0.043	0.751748
HP103_Pit2	HP396_Pit2	0	0	
HP103_Pit2	HP407_Pit2	0.0021	0.0036	1.714286
HP103_Pit2	HP492_Pit2	0.0021	0.0036	1.714286
HP103_Pit2	HP517-1_Pit2	0.0021	0.0036	1.714286
HP103_Pit2	HP577_Pit2	0	0	
HP103_Pit2	W0128_Pit2	0.0572	0.0423	0.73951
HP103_Pit2	GP104_Pit2	0.0594	0.043	0.723906
HP103_Pit2	GP124_Pit2	0.0594	0.043	0.723906
HP103_Pit2	GP540_Pit2	0.0572	0.043	0.751748
HP103_Pit2	Kasalath_Pit2	0.0594	0.043	0.723906
HP103_Pit2	W3105-1_Pit2	0.0594	0.043	0.723906
HP103_Pit2	GP295-1_Pit2	0.0549	0.0436	0.794171
HP103_Pit2	HP486_Pit2	0	0	
HP103_Pit2	GP62_Pit2	0.0578	0.0473	0.818339
HP390_Pit2	IL9_Pit2	0.0571	0.0456	0.798599
HP390_Pit2	Koshihikari_Pit2	0.0571	0.0456	0.798599
HP390_Pit2	KY131_Pit2	0.0571	0.0456	0.798599
HP390_Pit2	LG31_Pit2	0.0571	0.0456	0.798599
HP390_Pit2	UR28_Pit2	0.0021	0.003	1.428571
HP390_Pit2	WYG7_Pit2	0	0	
HP390_Pit2	GLA4_Pit2	0.0021	0.0036	1.714286
HP390_Pit2	GP3_Pit2	0.0021	0.0036	1.714286
HP390_Pit2	GP22_Pit2	0.0085	0.0073	0.858824
HP390_Pit2	GP51_Pit2	0.0594	0.043	0.723906
HP390_Pit2	GP772-1_Pit2	0	0	
HP390_Pit2	HP119_Pit2	0	0	

HP390_Pit2	HP263_Pit2	0	0	
HP390_Pit2	HP274_Pit2	0.0085	0.0073	0.858824
HP390_Pit2	HP327_Pit2	0	0	
HP390_Pit2	HP362-2_Pit2	0	0	
HP390_Pit2	HP383_Pit2	0.0594	0.043	0.723906
HP390_Pit2	HP396_Pit2	0.0021	0.0036	1.714286
HP390_Pit2	HP407_Pit2	0	0	
HP390_Pit2	HP492_Pit2	0	0	
HP390_Pit2	HP517-1_Pit2	0	0	
HP390_Pit2	HP577_Pit2	0.0021	0.0036	1.714286
HP390_Pit2	W0128_Pit2	0.0594	0.0423	0.712121
HP390_Pit2	GP104_Pit2	0.0617	0.043	0.696921
HP390_Pit2	GP124_Pit2	0.0617	0.043	0.696921
HP390_Pit2	GP540_Pit2	0.0594	0.043	0.723906
HP390_Pit2	Kasalath_Pit2	0.0617	0.043	0.696921
HP390_Pit2	W3105-1_Pit2	0.0617	0.043	0.696921
HP390_Pit2	GP295-1_Pit2	0.0572	0.0436	0.762238
HP390_Pit2	HP486_Pit2	0.0021	0.0036	1.714286
HP390_Pit2	GP62_Pit2	0.0601	0.0473	0.787022
IL9_Pit2	Koshihikari_Pit2	0	0	
IL9_Pit2	KY131_Pit2	0	0	
IL9_Pit2	LG31_Pit2	0	0	
IL9_Pit2	UR28_Pit2	0.0549	0.0449	0.817851
IL9_Pit2	WYG7_Pit2	0.0571	0.0456	0.798599
IL9_Pit2	GLA4_Pit2	0.0549	0.0456	0.830601
IL9_Pit2	GP3_Pit2	0.0549	0.0456	0.830601
IL9_Pit2	GP22_Pit2	0.0607	0.0452	0.744646
IL9_Pit2	GP51_Pit2	0.0064	0.0036	0.5625
IL9_Pit2	GP772-1_Pit2	0.0571	0.0456	0.798599
IL9_Pit2	HP119_Pit2	0.0571	0.0456	0.798599
IL9_Pit2	HP263_Pit2	0.0571	0.0456	0.798599
IL9_Pit2	HP274_Pit2	0.0607	0.0452	0.744646
IL9_Pit2	HP327_Pit2	0.0571	0.0456	0.798599
IL9_Pit2	HP362-2_Pit2	0.0571	0.0456	0.798599
IL9_Pit2	HP383_Pit2	0.0064	0.0036	0.5625
IL9_Pit2	HP396_Pit2	0.0549	0.0456	0.830601
IL9_Pit2	HP407_Pit2	0.0571	0.0456	0.798599
IL9_Pit2	HP492_Pit2	0.0571	0.0456	0.798599
IL9_Pit2	HP517-1_Pit2	0.0571	0.0456	0.798599
IL9_Pit2	HP577_Pit2	0.0549	0.0456	0.830601
IL9_Pit2	W0128_Pit2	0.0064	0.0042	0.65625
IL9_Pit2	GP104_Pit2	0.0085	0.0049	0.576471
IL9_Pit2	GP124_Pit2	0.0085	0.0049	0.576471
IL9_Pit2	GP540_Pit2	0.0064	0.0036	0.5625
IL9_Pit2	Kasalath_Pit2	0.0085	0.0049	0.576471
IL9_Pit2	W3105-1_Pit2	0.0085	0.0049	0.576471
IL9_Pit2	GP295-1_Pit2	0.0043	0.0042	0.976744
IL9_Pit2	HP486_Pit2	0.0549	0.0456	0.830601
IL9_Pit2	GP62_Pit2	0.0215	0.0141	0.655814
Koshihikari_Pi	KY131_Pit2	0	0	
Koshihikari_Pi	LG31_Pit2	0	0	
Koshihikari_Pi	UR28_Pit2	0.0549	0.0449	0.817851
Koshihikari_Pi	WYG7_Pit2	0.0571	0.0456	0.798599
Koshihikari_Pi	GLA4_Pit2	0.0549	0.0456	0.830601
Koshihikari_Pi	GP3_Pit2	0.0549	0.0456	0.830601
Koshihikari_Pi	GP22_Pit2	0.0607	0.0452	0.744646

Koshihikari_Pi	GP51_Pit2	0.0064	0.0036	0.5625
Koshihikari_Pi	GP772-1_Pit2	0.0571	0.0456	0.798599
Koshihikari_Pi	HP119_Pit2	0.0571	0.0456	0.798599
Koshihikari_Pi	HP263_Pit2	0.0571	0.0456	0.798599
Koshihikari_Pi	HP274_Pit2	0.0607	0.0452	0.744646
Koshihikari_Pi	HP327_Pit2	0.0571	0.0456	0.798599
Koshihikari_Pi	HP362-2_Pit2	0.0571	0.0456	0.798599
Koshihikari_Pi	HP383_Pit2	0.0064	0.0036	0.5625
Koshihikari_Pi	HP396_Pit2	0.0549	0.0456	0.830601
Koshihikari_Pi	HP407_Pit2	0.0571	0.0456	0.798599
Koshihikari_Pi	HP492_Pit2	0.0571	0.0456	0.798599
Koshihikari_Pi	HP517-1_Pit2	0.0571	0.0456	0.798599
Koshihikari_Pi	HP577_Pit2	0.0549	0.0456	0.830601
Koshihikari_Pi	W0128_Pit2	0.0064	0.0042	0.65625
Koshihikari_Pi	GP104_Pit2	0.0085	0.0049	0.576471
Koshihikari_Pi	GP124_Pit2	0.0085	0.0049	0.576471
Koshihikari_Pi	GP540_Pit2	0.0064	0.0036	0.5625
Koshihikari_Pi	Kasalath_Pit2	0.0085	0.0049	0.576471
Koshihikari_Pi	W3105-1_Pit2	0.0085	0.0049	0.576471
Koshihikari_Pi	GP295-1_Pit2	0.0043	0.0042	0.976744
Koshihikari_Pi	HP486_Pit2	0.0549	0.0456	0.830601
Koshihikari_Pi	GP62_Pit2	0.0215	0.0141	0.655814
KY131_Pit2	LG31_Pit2	0	0	
KY131_Pit2	UR28_Pit2	0.0549	0.0449	0.817851
KY131_Pit2	WYG7_Pit2	0.0571	0.0456	0.798599
KY131_Pit2	GLA4_Pit2	0.0549	0.0456	0.830601
KY131_Pit2	GP3_Pit2	0.0549	0.0456	0.830601
KY131_Pit2	GP22_Pit2	0.0607	0.0452	0.744646
KY131_Pit2	GP51_Pit2	0.0064	0.0036	0.5625
KY131_Pit2	GP772-1_Pit2	0.0571	0.0456	0.798599
KY131_Pit2	HP119_Pit2	0.0571	0.0456	0.798599
KY131_Pit2	HP263_Pit2	0.0571	0.0456	0.798599
KY131_Pit2	HP274_Pit2	0.0607	0.0452	0.744646
KY131_Pit2	HP327_Pit2	0.0571	0.0456	0.798599
KY131_Pit2	HP362-2_Pit2	0.0571	0.0456	0.798599
KY131_Pit2	HP383_Pit2	0.0064	0.0036	0.5625
KY131_Pit2	HP396_Pit2	0.0549	0.0456	0.830601
KY131_Pit2	HP407_Pit2	0.0571	0.0456	0.798599
KY131_Pit2	HP492_Pit2	0.0571	0.0456	0.798599
KY131_Pit2	HP517-1_Pit2	0.0571	0.0456	0.798599
KY131_Pit2	HP577_Pit2	0.0549	0.0456	0.830601
KY131_Pit2	W0128_Pit2	0.0064	0.0042	0.65625
KY131_Pit2	GP104_Pit2	0.0085	0.0049	0.576471
KY131_Pit2	GP124_Pit2	0.0085	0.0049	0.576471
KY131_Pit2	GP540_Pit2	0.0064	0.0036	0.5625
KY131_Pit2	Kasalath_Pit2	0.0085	0.0049	0.576471
KY131_Pit2	W3105-1_Pit2	0.0085	0.0049	0.576471
KY131_Pit2	GP295-1_Pit2	0.0043	0.0042	0.976744
KY131_Pit2	HP486_Pit2	0.0549	0.0456	0.830601
KY131_Pit2	GP62_Pit2	0.0215	0.0141	0.655814
LG31_Pit2	UR28_Pit2	0.0549	0.0449	0.817851
LG31_Pit2	WYG7_Pit2	0.0571	0.0456	0.798599
LG31_Pit2	GLA4_Pit2	0.0549	0.0456	0.830601
LG31_Pit2	GP3_Pit2	0.0549	0.0456	0.830601
LG31_Pit2	GP22_Pit2	0.0607	0.0452	0.744646
LG31_Pit2	GP51_Pit2	0.0064	0.0036	0.5625

LG31_Pit2	GP772-1_Pit2	0.0571	0.0456	0.798599
LG31_Pit2	HP119_Pit2	0.0571	0.0456	0.798599
LG31_Pit2	HP263_Pit2	0.0571	0.0456	0.798599
LG31_Pit2	HP274_Pit2	0.0607	0.0452	0.744646
LG31_Pit2	HP327_Pit2	0.0571	0.0456	0.798599
LG31_Pit2	HP362-2_Pit2	0.0571	0.0456	0.798599
LG31_Pit2	HP383_Pit2	0.0064	0.0036	0.5625
LG31_Pit2	HP396_Pit2	0.0549	0.0456	0.830601
LG31_Pit2	HP407_Pit2	0.0571	0.0456	0.798599
LG31_Pit2	HP492_Pit2	0.0571	0.0456	0.798599
LG31_Pit2	HP517-1_Pit2	0.0571	0.0456	0.798599
LG31_Pit2	HP577_Pit2	0.0549	0.0456	0.830601
LG31_Pit2	W0128_Pit2	0.0064	0.0042	0.65625
LG31_Pit2	GP104_Pit2	0.0085	0.0049	0.576471
LG31_Pit2	GP124_Pit2	0.0085	0.0049	0.576471
LG31_Pit2	GP540_Pit2	0.0064	0.0036	0.5625
LG31_Pit2	Kasalath_Pit2	0.0085	0.0049	0.576471
LG31_Pit2	W3105-1_Pit2	0.0085	0.0049	0.576471
LG31_Pit2	GP295-1_Pit2	0.0043	0.0042	0.976744
LG31_Pit2	HP486_Pit2	0.0549	0.0456	0.830601
LG31_Pit2	GP62_Pit2	0.0215	0.0141	0.655814
UR28_Pit2	WYG7_Pit2	0.0021	0.003	1.428571
UR28_Pit2	GLA4_Pit2	0	0.003	
UR28_Pit2	GP3_Pit2	0	0.003	
UR28_Pit2	GP22_Pit2	0.0106	0.0104	0.981132
UR28_Pit2	GP51_Pit2	0.0572	0.0423	0.73951
UR28_Pit2	GP772-1_Pit2	0.0021	0.003	1.428571
UR28_Pit2	HP119_Pit2	0.0021	0.003	1.428571
UR28_Pit2	HP263_Pit2	0.0021	0.003	1.428571
UR28_Pit2	HP274_Pit2	0.0106	0.0104	0.981132
UR28_Pit2	HP327_Pit2	0.0021	0.003	1.428571
UR28_Pit2	HP362-2_Pit2	0.0021	0.003	1.428571
UR28_Pit2	HP383_Pit2	0.0572	0.0423	0.73951
UR28_Pit2	HP396_Pit2	0	0.003	
UR28_Pit2	HP407_Pit2	0.0021	0.003	1.428571
UR28_Pit2	HP492_Pit2	0.0021	0.003	1.428571
UR28_Pit2	HP517-1_Pit2	0.0021	0.003	1.428571
UR28_Pit2	HP577_Pit2	0	0.003	
UR28_Pit2	W0128_Pit2	0.0572	0.0417	0.729021
UR28_Pit2	GP104_Pit2	0.0595	0.0423	0.710924
UR28_Pit2	GP124_Pit2	0.0595	0.0423	0.710924
UR28_Pit2	GP540_Pit2	0.0572	0.0423	0.73951
UR28_Pit2	Kasalath_Pit2	0.0595	0.0423	0.710924
UR28_Pit2	W3105-1_Pit2	0.0595	0.0423	0.710924
UR28_Pit2	GP295-1_Pit2	0.055	0.043	0.781818
UR28_Pit2	HP486_Pit2	0	0.003	
UR28_Pit2	GP62_Pit2	0.0579	0.0466	0.804836
WYG7_Pit2	GLA4_Pit2	0.0021	0.0036	1.714286
WYG7_Pit2	GP3_Pit2	0.0021	0.0036	1.714286
WYG7_Pit2	GP22_Pit2	0.0085	0.0073	0.858824
WYG7_Pit2	GP51_Pit2	0.0594	0.043	0.723906
WYG7_Pit2	GP772-1_Pit2	0	0	
WYG7_Pit2	HP119_Pit2	0	0	
WYG7_Pit2	HP263_Pit2	0	0	
WYG7_Pit2	HP274_Pit2	0.0085	0.0073	0.858824
WYG7_Pit2	HP327_Pit2	0	0	

WYG7_Pit2	HP362-2_Pit2	0	0	
WYG7_Pit2	HP383_Pit2	0.0594	0.043	0.723906
WYG7_Pit2	HP396_Pit2	0.0021	0.0036	1.714286
WYG7_Pit2	HP407_Pit2	0	0	
WYG7_Pit2	HP492_Pit2	0	0	
WYG7_Pit2	HP517-1_Pit2	0	0	
WYG7_Pit2	HP577_Pit2	0.0021	0.0036	1.714286
WYG7_Pit2	W0128_Pit2	0.0594	0.0423	0.712121
WYG7_Pit2	GP104_Pit2	0.0617	0.043	0.696921
WYG7_Pit2	GP124_Pit2	0.0617	0.043	0.696921
WYG7_Pit2	GP540_Pit2	0.0594	0.043	0.723906
WYG7_Pit2	Kasalath_Pit2	0.0617	0.043	0.696921
WYG7_Pit2	W3105-1_Pit2	0.0617	0.043	0.696921
WYG7_Pit2	GP295-1_Pit2	0.0572	0.0436	0.762238
WYG7_Pit2	HP486_Pit2	0.0021	0.0036	1.714286
WYG7_Pit2	GP62_Pit2	0.0601	0.0473	0.787022
GLA4_Pit2	GP3_Pit2	0	0	
GLA4_Pit2	GP22_Pit2	0.0106	0.011	1.037736
GLA4_Pit2	GP51_Pit2	0.0572	0.043	0.751748
GLA4_Pit2	GP772-1_Pit2	0.0021	0.0036	1.714286
GLA4_Pit2	HP119_Pit2	0.0021	0.0036	1.714286
GLA4_Pit2	HP263_Pit2	0.0021	0.0036	1.714286
GLA4_Pit2	HP274_Pit2	0.0106	0.011	1.037736
GLA4_Pit2	HP327_Pit2	0.0021	0.0036	1.714286
GLA4_Pit2	HP362-2_Pit2	0.0021	0.0036	1.714286
GLA4_Pit2	HP383_Pit2	0.0572	0.043	0.751748
GLA4_Pit2	HP396_Pit2	0	0	
GLA4_Pit2	HP407_Pit2	0.0021	0.0036	1.714286
GLA4_Pit2	HP492_Pit2	0.0021	0.0036	1.714286
GLA4_Pit2	HP517-1_Pit2	0.0021	0.0036	1.714286
GLA4_Pit2	HP577_Pit2	0	0	
GLA4_Pit2	W0128_Pit2	0.0572	0.0423	0.73951
GLA4_Pit2	GP104_Pit2	0.0594	0.043	0.723906
GLA4_Pit2	GP124_Pit2	0.0594	0.043	0.723906
GLA4_Pit2	GP540_Pit2	0.0572	0.043	0.751748
GLA4_Pit2	Kasalath_Pit2	0.0594	0.043	0.723906
GLA4_Pit2	W3105-1_Pit2	0.0594	0.043	0.723906
GLA4_Pit2	GP295-1_Pit2	0.0549	0.0436	0.794171
GLA4_Pit2	HP486_Pit2	0	0	
GLA4_Pit2	GP62_Pit2	0.0578	0.0473	0.818339
GP3_Pit2	GP22_Pit2	0.0106	0.011	1.037736
GP3_Pit2	GP51_Pit2	0.0572	0.043	0.751748
GP3_Pit2	GP772-1_Pit2	0.0021	0.0036	1.714286
GP3_Pit2	HP119_Pit2	0.0021	0.0036	1.714286
GP3_Pit2	HP263_Pit2	0.0021	0.0036	1.714286
GP3_Pit2	HP274_Pit2	0.0106	0.011	1.037736
GP3_Pit2	HP327_Pit2	0.0021	0.0036	1.714286
GP3_Pit2	HP362-2_Pit2	0.0021	0.0036	1.714286
GP3_Pit2	HP383_Pit2	0.0572	0.043	0.751748
GP3_Pit2	HP396_Pit2	0	0	
GP3_Pit2	HP407_Pit2	0.0021	0.0036	1.714286
GP3_Pit2	HP492_Pit2	0.0021	0.0036	1.714286
GP3_Pit2	HP517-1_Pit2	0.0021	0.0036	1.714286
GP3_Pit2	HP577_Pit2	0	0	
GP3_Pit2	W0128_Pit2	0.0572	0.0423	0.73951
GP3_Pit2	GP104_Pit2	0.0594	0.043	0.723906

GP3_Pit2	GP124_Pit2	0.0594	0.043	0.723906
GP3_Pit2	GP540_Pit2	0.0572	0.043	0.751748
GP3_Pit2	Kasalath_Pit2	0.0594	0.043	0.723906
GP3_Pit2	W3105-1_Pit2	0.0594	0.043	0.723906
GP3_Pit2	GP295-1_Pit2	0.0549	0.0436	0.794171
GP3_Pit2	HP486_Pit2	0	0	
GP3_Pit2	GP62_Pit2	0.0578	0.0473	0.818339
GP22_Pit2	GP51_Pit2	0.063	0.0426	0.67619
GP22_Pit2	GP772-1_Pit2	0.0085	0.0073	0.858824
GP22_Pit2	HP119_Pit2	0.0085	0.0073	0.858824
GP22_Pit2	HP263_Pit2	0.0085	0.0073	0.858824
GP22_Pit2	HP274_Pit2	0	0	
GP22_Pit2	HP327_Pit2	0.0085	0.0073	0.858824
GP22_Pit2	HP362-2_Pit2	0.0085	0.0073	0.858824
GP22_Pit2	HP383_Pit2	0.063	0.0426	0.67619
GP22_Pit2	HP396_Pit2	0.0106	0.011	1.037736
GP22_Pit2	HP407_Pit2	0.0085	0.0073	0.858824
GP22_Pit2	HP492_Pit2	0.0085	0.0073	0.858824
GP22_Pit2	HP517-1_Pit2	0.0085	0.0073	0.858824
GP22_Pit2	HP577_Pit2	0.0106	0.011	1.037736
GP22_Pit2	W0128_Pit2	0.063	0.042	0.666667
GP22_Pit2	GP104_Pit2	0.0652	0.0426	0.653374
GP22_Pit2	GP124_Pit2	0.0652	0.0426	0.653374
GP22_Pit2	GP540_Pit2	0.063	0.0426	0.67619
GP22_Pit2	Kasalath_Pit2	0.0652	0.0426	0.653374
GP22_Pit2	W3105-1_Pit2	0.0652	0.0426	0.653374
GP22_Pit2	GP295-1_Pit2	0.0607	0.0433	0.713344
GP22_Pit2	HP486_Pit2	0.0106	0.011	1.037736
GP22_Pit2	GP62_Pit2	0.0593	0.0475	0.801012
GP51_Pit2	GP772-1_Pit2	0.0594	0.043	0.723906
GP51_Pit2	HP119_Pit2	0.0594	0.043	0.723906
GP51_Pit2	HP263_Pit2	0.0594	0.043	0.723906
GP51_Pit2	HP274_Pit2	0.063	0.0426	0.67619
GP51_Pit2	HP327_Pit2	0.0594	0.043	0.723906
GP51_Pit2	HP362-2_Pit2	0.0594	0.043	0.723906
GP51_Pit2	HP383_Pit2	0	0	
GP51_Pit2	HP396_Pit2	0.0572	0.043	0.751748
GP51_Pit2	HP407_Pit2	0.0594	0.043	0.723906
GP51_Pit2	HP492_Pit2	0.0594	0.043	0.723906
GP51_Pit2	HP517-1_Pit2	0.0594	0.043	0.723906
GP51_Pit2	HP577_Pit2	0.0572	0.043	0.751748
GP51_Pit2	W0128_Pit2	0.0085	0.0006	0.070588
GP51_Pit2	GP104_Pit2	0.0107	0.0012	0.11215
GP51_Pit2	GP124_Pit2	0.0107	0.0012	0.11215
GP51_Pit2	GP540_Pit2	0	0	
GP51_Pit2	Kasalath_Pit2	0.0107	0.0012	0.11215
GP51_Pit2	W3105-1_Pit2	0.0107	0.0012	0.11215
GP51_Pit2	GP295-1_Pit2	0.0064	0.0006	0.09375
GP51_Pit2	HP486_Pit2	0.0572	0.043	0.751748
GP51_Pit2	GP62_Pit2	0.0237	0.0104	0.438819
GP772-1_Pit2	HP119_Pit2	0	0	
GP772-1_Pit2	HP263_Pit2	0	0	
GP772-1_Pit2	HP274_Pit2	0.0085	0.0073	0.858824
GP772-1_Pit2	HP327_Pit2	0	0	
GP772-1_Pit2	HP362-2_Pit2	0	0	
GP772-1_Pit2	HP383_Pit2	0.0594	0.043	0.723906

GP772-1_Pit2	HP396_Pit2	0.0021	0.0036	1.714286
GP772-1_Pit2	HP407_Pit2	0	0	
GP772-1_Pit2	HP492_Pit2	0	0	
GP772-1_Pit2	HP517-1_Pit2	0	0	
GP772-1_Pit2	HP577_Pit2	0.0021	0.0036	1.714286
GP772-1_Pit2	W0128_Pit2	0.0594	0.0423	0.712121
GP772-1_Pit2	GP104_Pit2	0.0617	0.043	0.696921
GP772-1_Pit2	GP124_Pit2	0.0617	0.043	0.696921
GP772-1_Pit2	GP540_Pit2	0.0594	0.043	0.723906
GP772-1_Pit2	Kasalath_Pit2	0.0617	0.043	0.696921
GP772-1_Pit2	W3105-1_Pit2	0.0617	0.043	0.696921
GP772-1_Pit2	GP295-1_Pit2	0.0572	0.0436	0.762238
GP772-1_Pit2	HP486_Pit2	0.0021	0.0036	1.714286
GP772-1_Pit2	GP62_Pit2	0.0601	0.0473	0.787022
HP119_Pit2	HP263_Pit2	0	0	
HP119_Pit2	HP274_Pit2	0.0085	0.0073	0.858824
HP119_Pit2	HP327_Pit2	0	0	
HP119_Pit2	HP362-2_Pit2	0	0	
HP119_Pit2	HP383_Pit2	0.0594	0.043	0.723906
HP119_Pit2	HP396_Pit2	0.0021	0.0036	1.714286
HP119_Pit2	HP407_Pit2	0	0	
HP119_Pit2	HP492_Pit2	0	0	
HP119_Pit2	HP517-1_Pit2	0	0	
HP119_Pit2	HP577_Pit2	0.0021	0.0036	1.714286
HP119_Pit2	W0128_Pit2	0.0594	0.0423	0.712121
HP119_Pit2	GP104_Pit2	0.0617	0.043	0.696921
HP119_Pit2	GP124_Pit2	0.0617	0.043	0.696921
HP119_Pit2	GP540_Pit2	0.0594	0.043	0.723906
HP119_Pit2	Kasalath_Pit2	0.0617	0.043	0.696921
HP119_Pit2	W3105-1_Pit2	0.0617	0.043	0.696921
HP119_Pit2	GP295-1_Pit2	0.0572	0.0436	0.762238
HP119_Pit2	HP486_Pit2	0.0021	0.0036	1.714286
HP119_Pit2	GP62_Pit2	0.0601	0.0473	0.787022
HP263_Pit2	HP274_Pit2	0.0085	0.0073	0.858824
HP263_Pit2	HP327_Pit2	0	0	
HP263_Pit2	HP362-2_Pit2	0	0	
HP263_Pit2	HP383_Pit2	0.0594	0.043	0.723906
HP263_Pit2	HP396_Pit2	0.0021	0.0036	1.714286
HP263_Pit2	HP407_Pit2	0	0	
HP263_Pit2	HP492_Pit2	0	0	
HP263_Pit2	HP517-1_Pit2	0	0	
HP263_Pit2	HP577_Pit2	0.0021	0.0036	1.714286
HP263_Pit2	W0128_Pit2	0.0594	0.0423	0.712121
HP263_Pit2	GP104_Pit2	0.0617	0.043	0.696921
HP263_Pit2	GP124_Pit2	0.0617	0.043	0.696921
HP263_Pit2	GP540_Pit2	0.0594	0.043	0.723906
HP263_Pit2	Kasalath_Pit2	0.0617	0.043	0.696921
HP263_Pit2	W3105-1_Pit2	0.0617	0.043	0.696921
HP263_Pit2	GP295-1_Pit2	0.0572	0.0436	0.762238
HP263_Pit2	HP486_Pit2	0.0021	0.0036	1.714286
HP263_Pit2	GP62_Pit2	0.0601	0.0473	0.787022
HP274_Pit2	HP327_Pit2	0.0085	0.0073	0.858824
HP274_Pit2	HP362-2_Pit2	0.0085	0.0073	0.858824
HP274_Pit2	HP383_Pit2	0.063	0.0426	0.67619
HP274_Pit2	HP396_Pit2	0.0106	0.011	1.037736
HP274_Pit2	HP407_Pit2	0.0085	0.0073	0.858824

HP274_Pit2	HP492_Pit2	0.0085	0.0073	0.858824
HP274_Pit2	HP517-1_Pit2	0.0085	0.0073	0.858824
HP274_Pit2	HP577_Pit2	0.0106	0.011	1.037736
HP274_Pit2	W0128_Pit2	0.063	0.042	0.666667
HP274_Pit2	GP104_Pit2	0.0652	0.0426	0.653374
HP274_Pit2	GP124_Pit2	0.0652	0.0426	0.653374
HP274_Pit2	GP540_Pit2	0.063	0.0426	0.67619
HP274_Pit2	Kasalath_Pit2	0.0652	0.0426	0.653374
HP274_Pit2	W3105-1_Pit2	0.0652	0.0426	0.653374
HP274_Pit2	GP295-1_Pit2	0.0607	0.0433	0.713344
HP274_Pit2	HP486_Pit2	0.0106	0.011	1.037736
HP274_Pit2	GP62_Pit2	0.0593	0.0475	0.801012
HP327_Pit2	HP362-2_Pit2	0	0	
HP327_Pit2	HP383_Pit2	0.0594	0.043	0.723906
HP327_Pit2	HP396_Pit2	0.0021	0.0036	1.714286
HP327_Pit2	HP407_Pit2	0	0	
HP327_Pit2	HP492_Pit2	0	0	
HP327_Pit2	HP517-1_Pit2	0	0	
HP327_Pit2	HP577_Pit2	0.0021	0.0036	1.714286
HP327_Pit2	W0128_Pit2	0.0594	0.0423	0.712121
HP327_Pit2	GP104_Pit2	0.0617	0.043	0.696921
HP327_Pit2	GP124_Pit2	0.0617	0.043	0.696921
HP327_Pit2	GP540_Pit2	0.0594	0.043	0.723906
HP327_Pit2	Kasalath_Pit2	0.0617	0.043	0.696921
HP327_Pit2	W3105-1_Pit2	0.0617	0.043	0.696921
HP327_Pit2	GP295-1_Pit2	0.0572	0.0436	0.762238
HP327_Pit2	HP486_Pit2	0.0021	0.0036	1.714286
HP327_Pit2	GP62_Pit2	0.0601	0.0473	0.787022
HP362-2_Pit2	HP383_Pit2	0.0594	0.043	0.723906
HP362-2_Pit2	HP396_Pit2	0.0021	0.0036	1.714286
HP362-2_Pit2	HP407_Pit2	0	0	
HP362-2_Pit2	HP492_Pit2	0	0	
HP362-2_Pit2	HP517-1_Pit2	0	0	
HP362-2_Pit2	HP577_Pit2	0.0021	0.0036	1.714286
HP362-2_Pit2	W0128_Pit2	0.0594	0.0423	0.712121
HP362-2_Pit2	GP104_Pit2	0.0617	0.043	0.696921
HP362-2_Pit2	GP124_Pit2	0.0617	0.043	0.696921
HP362-2_Pit2	GP540_Pit2	0.0594	0.043	0.723906
HP362-2_Pit2	Kasalath_Pit2	0.0617	0.043	0.696921
HP362-2_Pit2	W3105-1_Pit2	0.0617	0.043	0.696921
HP362-2_Pit2	GP295-1_Pit2	0.0572	0.0436	0.762238
HP362-2_Pit2	HP486_Pit2	0.0021	0.0036	1.714286
HP362-2_Pit2	GP62_Pit2	0.0601	0.0473	0.787022
HP383_Pit2	HP396_Pit2	0.0572	0.043	0.751748
HP383_Pit2	HP407_Pit2	0.0594	0.043	0.723906
HP383_Pit2	HP492_Pit2	0.0594	0.043	0.723906
HP383_Pit2	HP517-1_Pit2	0.0594	0.043	0.723906
HP383_Pit2	HP577_Pit2	0.0572	0.043	0.751748
HP383_Pit2	W0128_Pit2	0.0085	0.0006	0.070588
HP383_Pit2	GP104_Pit2	0.0107	0.0012	0.11215
HP383_Pit2	GP124_Pit2	0.0107	0.0012	0.11215
HP383_Pit2	GP540_Pit2	0	0	
HP383_Pit2	Kasalath_Pit2	0.0107	0.0012	0.11215
HP383_Pit2	W3105-1_Pit2	0.0107	0.0012	0.11215
HP383_Pit2	GP295-1_Pit2	0.0064	0.0006	0.09375
HP383_Pit2	HP486_Pit2	0.0572	0.043	0.751748

HP383_Pit2	GP62_Pit2	0.0237	0.0104	0.438819
HP396_Pit2	HP407_Pit2	0.0021	0.0036	1.714286
HP396_Pit2	HP492_Pit2	0.0021	0.0036	1.714286
HP396_Pit2	HP517-1_Pit2	0.0021	0.0036	1.714286
HP396_Pit2	HP577_Pit2	0	0	
HP396_Pit2	W0128_Pit2	0.0572	0.0423	0.73951
HP396_Pit2	GP104_Pit2	0.0594	0.043	0.723906
HP396_Pit2	GP124_Pit2	0.0594	0.043	0.723906
HP396_Pit2	GP540_Pit2	0.0572	0.043	0.751748
HP396_Pit2	Kasalath_Pit2	0.0594	0.043	0.723906
HP396_Pit2	W3105-1_Pit2	0.0594	0.043	0.723906
HP396_Pit2	GP295-1_Pit2	0.0549	0.0436	0.794171
HP396_Pit2	HP486_Pit2	0	0	
HP396_Pit2	GP62_Pit2	0.0578	0.0473	0.818339
HP407_Pit2	HP492_Pit2	0	0	
HP407_Pit2	HP517-1_Pit2	0	0	
HP407_Pit2	HP577_Pit2	0.0021	0.0036	1.714286
HP407_Pit2	W0128_Pit2	0.0594	0.0423	0.712121
HP407_Pit2	GP104_Pit2	0.0617	0.043	0.696921
HP407_Pit2	GP124_Pit2	0.0617	0.043	0.696921
HP407_Pit2	GP540_Pit2	0.0594	0.043	0.723906
HP407_Pit2	Kasalath_Pit2	0.0617	0.043	0.696921
HP407_Pit2	W3105-1_Pit2	0.0617	0.043	0.696921
HP407_Pit2	GP295-1_Pit2	0.0572	0.0436	0.762238
HP407_Pit2	HP486_Pit2	0.0021	0.0036	1.714286
HP407_Pit2	GP62_Pit2	0.0601	0.0473	0.787022
HP492_Pit2	HP517-1_Pit2	0	0	
HP492_Pit2	HP577_Pit2	0.0021	0.0036	1.714286
HP492_Pit2	W0128_Pit2	0.0594	0.0423	0.712121
HP492_Pit2	GP104_Pit2	0.0617	0.043	0.696921
HP492_Pit2	GP124_Pit2	0.0617	0.043	0.696921
HP492_Pit2	GP540_Pit2	0.0594	0.043	0.723906
HP492_Pit2	Kasalath_Pit2	0.0617	0.043	0.696921
HP492_Pit2	W3105-1_Pit2	0.0617	0.043	0.696921
HP492_Pit2	GP295-1_Pit2	0.0572	0.0436	0.762238
HP492_Pit2	HP486_Pit2	0.0021	0.0036	1.714286
HP492_Pit2	GP62_Pit2	0.0601	0.0473	0.787022
HP517-1_Pit2	HP577_Pit2	0.0021	0.0036	1.714286
HP517-1_Pit2	W0128_Pit2	0.0594	0.0423	0.712121
HP517-1_Pit2	GP104_Pit2	0.0617	0.043	0.696921
HP517-1_Pit2	GP124_Pit2	0.0617	0.043	0.696921
HP517-1_Pit2	GP540_Pit2	0.0594	0.043	0.723906
HP517-1_Pit2	Kasalath_Pit2	0.0617	0.043	0.696921
HP517-1_Pit2	W3105-1_Pit2	0.0617	0.043	0.696921
HP517-1_Pit2	GP295-1_Pit2	0.0572	0.0436	0.762238
HP517-1_Pit2	HP486_Pit2	0.0021	0.0036	1.714286
HP517-1_Pit2	GP62_Pit2	0.0601	0.0473	0.787022
HP577_Pit2	W0128_Pit2	0.0572	0.0423	0.73951
HP577_Pit2	GP104_Pit2	0.0594	0.043	0.723906
HP577_Pit2	GP124_Pit2	0.0594	0.043	0.723906
HP577_Pit2	GP540_Pit2	0.0572	0.043	0.751748
HP577_Pit2	Kasalath_Pit2	0.0594	0.043	0.723906
HP577_Pit2	W3105-1_Pit2	0.0594	0.043	0.723906
HP577_Pit2	GP295-1_Pit2	0.0549	0.0436	0.794171
HP577_Pit2	HP486_Pit2	0	0	
HP577_Pit2	GP62_Pit2	0.0578	0.0473	0.818339

W0128_Pit2	GP104_Pit2	0.0021	0.0006	0.285714
W0128_Pit2	GP124_Pit2	0.0021	0.0006	0.285714
W0128_Pit2	GP540_Pit2	0.0085	0.0006	0.070588
W0128_Pit2	Kasalath_Pit2	0.0021	0.0006	0.285714
W0128_Pit2	W3105-1_Pit2	0.0021	0.0006	0.285714
W0128_Pit2	GP295-1_Pit2	0.0064	0.0012	0.1875
W0128_Pit2	HP486_Pit2	0.0572	0.0423	0.73951
W0128_Pit2	GP62_Pit2	0.015	0.0097	0.646667
GP104_Pit2	GP124_Pit2	0	0	
GP104_Pit2	GP540_Pit2	0.0107	0.0012	0.11215
GP104_Pit2	Kasalath_Pit2	0	0	
GP104_Pit2	W3105-1_Pit2	0	0	
GP104_Pit2	GP295-1_Pit2	0.0085	0.0018	0.211765
GP104_Pit2	HP486_Pit2	0.0594	0.043	0.723906
GP104_Pit2	GP62_Pit2	0.0128	0.0091	0.710938
GP124_Pit2	GP540_Pit2	0.0107	0.0012	0.11215
GP124_Pit2	Kasalath_Pit2	0	0	
GP124_Pit2	W3105-1_Pit2	0	0	
GP124_Pit2	GP295-1_Pit2	0.0085	0.0018	0.211765
GP124_Pit2	HP486_Pit2	0.0594	0.043	0.723906
GP124_Pit2	GP62_Pit2	0.0128	0.0091	0.710938
GP540_Pit2	Kasalath_Pit2	0.0107	0.0012	0.11215
GP540_Pit2	W3105-1_Pit2	0.0107	0.0012	0.11215
GP540_Pit2	GP295-1_Pit2	0.0064	0.0006	0.09375
GP540_Pit2	HP486_Pit2	0.0572	0.043	0.751748
GP540_Pit2	GP62_Pit2	0.0237	0.0104	0.438819
Kasalath_Pit2	W3105-1_Pit2	0	0	
Kasalath_Pit2	GP295-1_Pit2	0.0085	0.0018	0.211765
Kasalath_Pit2	HP486_Pit2	0.0594	0.043	0.723906
Kasalath_Pit2	GP62_Pit2	0.0128	0.0091	0.710938
W3105-1_Pit2	GP295-1_Pit2	0.0085	0.0018	0.211765
W3105-1_Pit2	HP486_Pit2	0.0594	0.043	0.723906
W3105-1_Pit2	GP62_Pit2	0.0128	0.0091	0.710938
GP295-1_Pit2	HP486_Pit2	0.0549	0.0436	0.794171
GP295-1_Pit2	GP62_Pit2	0.0215	0.011	0.511628
HP486_Pit2	GP62_Pit2	0.0578	0.0473	0.818339
